# Unexpected Intrinsic Lability of Thiol-Functionalized Carboxylate Imidazolium Ionic Liquids

**DOI:** 10.3390/molecules24193571

**Published:** 2019-10-03

**Authors:** Andrea Mezzetta, Lorenzo Poderelli, Felicia D’Andrea, Christian Silvio Pomelli, Cinzia Chiappe, Lorenzo Guazzelli

**Affiliations:** Department of Pharmacy, University of Pisa, Via Bonanno 33, 56126 Pisa, Italy; andrea.mezzetta@for.unipi.it (A.M.); l.poderelli@gmail.com (L.P.); christian.pomelli@unipi.it (C.S.P.); cinzia.chiappe@unipi.it (C.C.)

**Keywords:** thiol-functionalized ionic liquids, redox systems, thermal-promoted decomposition, imidazole 2-thione

## Abstract

New thiol-functionalized carboxylate ionic liquids (ILs), varying both for the cation and for the anion structures, have been prepared as new potential redox switching systems by reacting either 3-mercapto propionic acid (3-MPA) or *N*-acetyl-cysteine (NAC) with commercially available methyl carbonate ILs. Different ratios of thiol/disulfide ILs were obtained depending both on the acid employed in the neutralization reaction and on the reaction conditions used. Surprisingly, the imidazolium ILs displayed limited thermal stability which resulted in the formation of an imidazole 2-thione and a new sulfide ionic liquid. Conversely, the formation of the imidazole 2-thione was not observed when phosphonium disulfide ILs were heated, thus confirming the involvement of the imidazolium ring in an unexpected side reaction. An insight into the mechanism of the decomposition has been provided by means of DFT calculations.

## 1. Introduction

Ionic liquids (ILs) are organic salts, composed by an organic cation and an organic or an inorganic anion, with a melting point below 100 °C [1]. ILs are usually praised for some peculiar physico-chemical properties such as practically null vapor pressure, high thermal stability and low flammability. However, caution should be used as these properties can vary substantially between different types of ILs. For instance, some ILs are actually distillable [2] and others have been proven to generate flammable gases during their thermal decomposition [3]. The undeniable most attractive feature of ILs lies in the possibility to select or design suitable matching ions for a given application [4]. Therefore, the potential of ILs has been explored in several different areas such as biomass modification [5,6,7], electrochemistry [8,9], material science [10] and pharmaceutically-related applications [11].

ILs presenting additional functional groups either on the cation or on the anion are known as ‘’task-specific’’ ionic liquids [12]. Thiol-functionalized ionic liquids, which have been less studied than the corresponding hydroxyl functionalized ILs, belong to this class of ILs. Usually, the thiol functional group, as for most task-specific ILs, has been introduced on the cation and the resulting ILs have been explored as potential redox switching systems, exploiting the thiol/disulfide couple (with interesting effects on both the viscosity [13] and the polarity of the system [14]), as modulator of wettability of gold surfaces [15] and for the selective extraction of Cd(II) from water and food samples [16]. An example of thiol functionalization of the anion has also been reported and these ILs’ systems have been studied as enhancers of nanotube dispersion in a rubber matrix [17]. Recently, a new access to thioalkylimidazolium ILs exploiting odorless intermediates and their use as ligands in Ullmann and Suzuki couplings have been reported [18]. As part of an ongoing project aimed at identifying ILs characterized by switching properties, the preparation of new thiol-functionalized carboxylate ILs is described. The unexpected isolation of a decomposition product at relatively low temperatures for imidazolium ILs, which was unpredictable on the basis of thermogravimetric analysis, and the rationalization of this result are also reported.

## 2. Results and Discussion

**Scheme 1:**
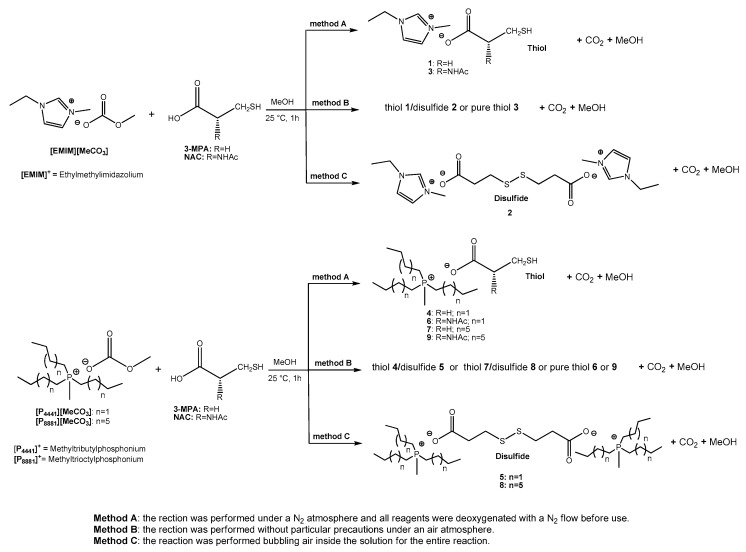
Preparation of thiol-functionalized carboxylate ionic liquids (ILs).

Target ILs were prepared by adding an equimolar amount of either 3-mercapto propionic acid (3-MPA) or *N*-acetyl-cysteine (NAC) to methanolic solutions of commercial methyl ethylimidazolium-, trioctyl methyl phosphonium-, and tributylmethyl phosphonium methyl carbonate ILs (Scheme 1). In agreement with what was reported for other carboxylic acids [19], a smooth neutralization reaction took place at room temperature and the final ILs were recovered after removal of the solvent. This approach is particularly convenient if compared to other protocols for the preparation of carboxylate ILs, which involve the use of silver salts or resins [20]. Following this procedure, a mixture of the thiol IL and the corresponding disulfide dicarboxylate IL was obtained when the reaction was performed with 3-MPA (entries 2, 6, 10 Table 1). Conversely, when NAC was employed, only the thiol IL was isolated (entries 4, 8, 12 Table 1). Attempts to boost the reaction in either direction were carried out. Indeed, by working under a nitrogen atmosphere after deoxygenation of the reagents (method A), almost solely the thiol IL was obtained (entries 1, 5, 9 Table 1). On the other hand, by bubbling air during the entire course of the reaction (method C), it was possible to isolate the disulfide IL (entries 3, 7, 11 Table 1). It is also worth mentioning that thiol ILs convert into the disulfide ILs over time depending on storage conditions.

**Figure 1:**
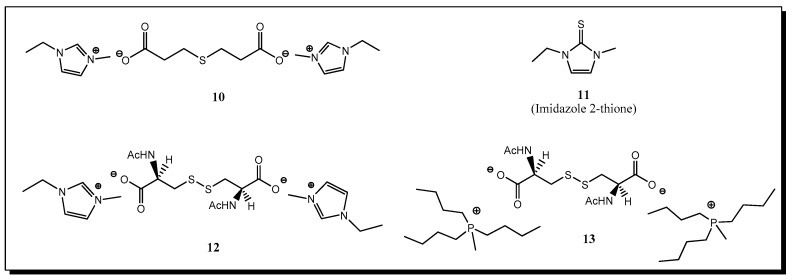
Compounds isolated for imidazolium ILs **2**, **3** and **6** after heating.

Therefore, only the thermal stability of NAC-derived thiol ILs (**3**, **6**, **9**, Scheme 1) and of dicarboxylate disulfide ILs (**2**, **5**, **8**, Scheme 1) was investigated by thermal gravimetric analysis. The ILs investigated displayed multiple degradation steps (please refer to the SI) and, apart from disulfide **2**, presented the first decomposition event at about 200 °C (T_onset_ values reported in Table 1), in good agreement with previous findings on carboxylate ILs [21]. To better understand the conversion of these thiols into disulfides and get an insight into the actual thermal stability in real experimental conditions, selected ILs (**2**, **3**, **5** and **6**, Scheme 1) were heated to 120 °C for 4 h. No degradations were observed for [P_4441_]-based ILs, but only the conversion into the corresponding disulfide IL **13** (Figure 1) for **6**. Instead, a different and unpredictable behavior was found for the two imidazolium ILs **2** and **3**. Indeed, IL **2** afforded the dicarboxylate sulfide IL **10** (Figure 1) and the known imidazole 2-thione **11** [22] (45:55 ratio, Figure 1), while IL **3** was converted into the disulfide IL **12** after 4h with little but detectable amounts of the 2H-imidazole thione **11**, ca 12%. The most thermally sensitive disulfide IL **2** was heated also to lower temperatures and even at 90 °C (8h) decomposed completely into the shown products.

These results underline an active role played by the imidazolium cation in triggering the decomposition process. It is worth mentioning that imidazolium 2-thiones attracted some interest recently for the preparation of nonvolatile alkylating agents [23,24].

**Figure 2:**
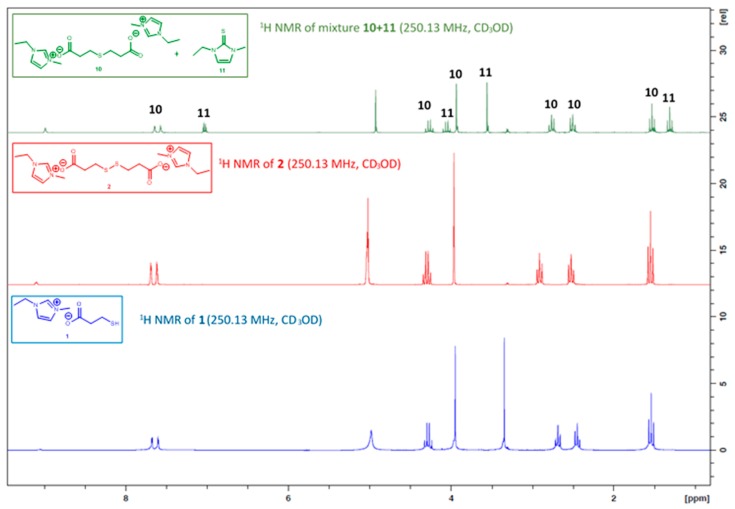
^1^H-NMR spectra of compound **1** (blue), compound **2** (red), and mixture of **10** and **11** obtained by heating **2** at 120 °C for 4 h (green).

In Figure 2, the ^1^H-NMR spectra of the thiol IL **1** (bottom), of the disulfide IL **2** (middle) and of the corresponding mixture of sulfide IL **10** and imidazole 2-thione **11** (up) obtained after heating compound **2** are compared.

It has been recently reported [25] that neat 1-ethyl-3-methyl-imidazolium acetate is able to react with disulfide species to form the same imidazole 2-thione observed by us. The evolution of the reaction was followed by ^1^H-NMR and it was proposed that the reaction proceeded through the formation of a carbene, by the abstraction of the H-2 proton from the imidazolium promoted by the acetate, followed by a nucleophilic substitution on the disulfide. The obtained imidazolium-thio intermediate furnished the imidazole 2-thione and the sulfide product. Indeed, the formation of carbene from imidazolium ILs due to the basicity of the anion has been reported [26] and even exploited before [27]. 

The same mechanism previously described [25] seems to be applicable here, although in our case the structural components required for decomposition, namely the imidazolium moiety, the basic carboxylate group and the disulfide bond, are already present in the dianionic disulfide IL **2** and are not part of separate entities. Therefore, this class of thiol imidazolium ILs is intrinsically thermally labile, while previously reported thiol containing ILs, lacking one of the above mentioned structural features, are not.

To get a further insight into the decomposition mechanism and verify if the suggested mechanism would work in the case under investigation here, Density Functional Theory (DFT) calculations were performed using the Gaussian 16 suite of programs [28]. All calculations have been performed at the B3LYP/6-311++G(d,p) level successfully used in the literature to study ionic liquids [29]. Geometries of all structures cited in this section are reported in the Supplementary Data.

**Figure 3:**
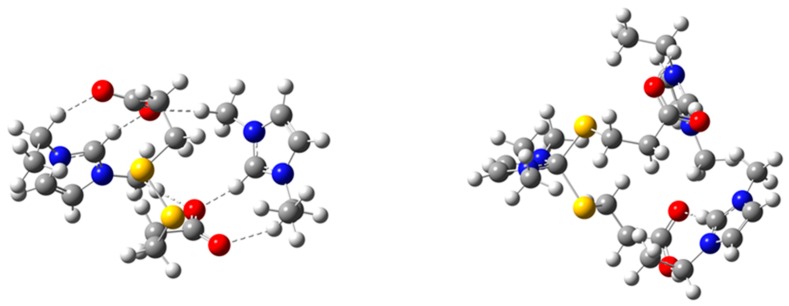
**Left**: DFT optimized structure of the minimal neutral cluster. Each carboxylate group forms two hydrogen bonds with one EMIM cation and one with the other. The –S-S- moiety presents no steric hindrance. **Right**: an EMIM derived carbene breaks the S-S bond leading to the first reaction intermediate (red: oxygen atom, blue: nitrogen atom, yellow: sulfur atom, grey: carbon atom, white: hydrogen atom).

First, a minimal neutral cluster constituted by two EMIM cations and one dicarboxylate disulfide has been considered. Some conformers of this cluster have been generated. The most stable structure is reported and described in Figure 3 (left). The possibility of generating a carbene structure internally by proton transfer from one of the EMIM cations to a carboxylate moiety has been unsuccessfully explored. Instead, for a minimal cluster augmented by an external EMIM derived carbene, a reactive path to an imidazolium thio-intermediate has been obtained (Figure 3, right). Furthermore, a not-reactive encounter complex and a transition state connecting the two last mentioned structures has been found (these structures can be found in supplementary data). The imidazolium thio-intermediate is less stable than the encounter complex by 71.2 KJ/mol and more stable than the transition state by 34.4 KJ/mol. Also, it presents a complete cleavage of the S-S bond replaced by two C-S bonds. The evolution of this structure to the further intermediates and products will be discussed in a full paper.

## 3. Materials and Methods

NMR spectra were recorded with a Bruker Avance II (Billerica, MA, USA) operating at 250.13 (^1^H) and 62.9 MHz (^13^C) or Bruker Avance 400 MHz at 24 °C in the stated solvent (Me_4_Si was used as the internal standard for CDCl_3_). The first order proton chemical shifts δ are referenced to either residual CD_3_OD (δ_H_ 3.31, δ_C_ 49.0) and *J*-values are given in Hz. The assignments were made with the aid of DEPT, HSQC and COSY experiments and in the case of mixtures, assignments were made by referring to the differences in the peak intensities. All reagents were obtained commercially and used without further purification. Commercial methylcarbonate ILs in methanol solution were obtained from Proionic GmbH (Grambach, Austria). The thermal stability of the synthesized ILs was investigated by thermal gravimetric analysis (TGA), conducted in a TA Instruments Q500 TGA (New Castle, DE, USA). ILs (15–20 mg) was heated in a platinum crucible as sample holders. First, the heating mode was set to isothermal at 50 °C in N_2_ (100 mL/min) for 30 min. Then, IL was heated from 40 to 600 °C with a heating rate of 10 °C/min under nitrogen (100 mL/min). Mass change was recorded as a function of temperature and time. TGA experiments were carried out in triplicate.

## 4. Conclusions

In conclusion, new ILs presenting a thiol-functionalized carboxylate anion have been prepared by exploiting a neutralization reaction between methyl carbonate ILs and either 3-mercapto propionic acid or *N*-acetyl-cysteine. Different ratios of thiol/disulfide have been obtained depending on the experimental conditions used. Thermogravimetric analysis drastically overestimated the thermal stability of the imidazolium ILs, since a temperature-promoted decomposition, which caused the formation of a dicarboxylate sulfide IL and the imidazole 2-thione, was experimentally observed even at 90 °C. DFT calculations gave us an insight into the decomposition mechanism, even if further studies are needed to elucidate it completely. Although these novel ILs are undoubtedly of interest for their redox switching potential, their thermal lability represents a potential drawback to be carefully considered.

Thiol/disulfide ionic liquids could be employed in several different applications as for instance in thiol-ene click reactions for the modification of material surfaces or as gold nanoparticle stabilizers. However, we are mainly interested in exploiting the change of their physico-chemical properties (especially polarity) due to the reversible transformation of the thiol ionic liquid into a disulfide ionic liquid. As a result, the scope of the prepared ILs in the development of new extraction media is currently under investigation and results will be presented in due course.

## Figures and Tables

**Table 1:** Thiol carboxylate ILs and dianionic disulfide ILs and their mixture obtained by methods A–C.

Entry	IL MeCO_3_	Acid	Method A	Method B	Method C	Products (N)	T_onset_ (°C) ^1^
1	[EMIM]	3-MPA	X			thiol (**1**)	--
2	[EMIM]	3-MPA		X		thiol (**1**)/disulfide (**2**) 88:12	--
3	[EMIM]	3-MPA			X	disulfide (**2**)	171
4	[EMIM]	NAC		X		thiol (**3**)	206
5	[P_4441_]	3-MPA	X			thiol (**4**) impure of 5 (7%)	--
6	[P_4441_]	3-MPA		X		thiol (**4**)/disulfide (**5**) 40:60	--
7	[P_4441_]	3-MPA			X	disulfide (**5**)	198
8	[P_4441_]	NAC		X		thiol (**6**)	193
9	[P_8881_]	3-MPA	X			thiol (**7**) impure of 8 (12%)	--
10	[P_8881_]	3-MPA		X		thiol (**7**)/disulfide (**8**) 79:21	--
11	[P_8881_]	3-MPA			X	disulfide (**8**)	200
12	[P_8881_]	NAC		X		thiol (**9**)	202

^1^ scanning rate 10K/min.

## Supplementary Materials

The following are available online at https://www.mdpi.com/1420-3049/24/19/3571/s1, all the detailed experimental procedures, NMR spectra, TGA analysis.
