# Supplementary material for: Unexpected Intrinsic Lability of Thiol-Functionalized Carboxylate Imidazolium Ionic Liquids
Source: Molecules. 2019 Oct 3;24(19):3571. doi: 10.3390/molecules24193571 (PMC6804084; doi:10.3390/molecules24193571)
Supplement: Supplementary file 1 [file molecules-24-03571-s001.pdf]

Unexpected intrinsic lability of thiol-functionalized carboxylate imidazolium ionic liquids

Andrea Mezzetta, Lorenzo Poderelli, Felicia D'Andrea, Christian Silvio Pomelli, Cinzia Chiappe, Lorenzo Guazzelli*

Department of Pharmacy, University of Pisa, Via Bonanno 33, 56126, Pisa, Italy

Corresponding Author: lorenzo.guazzelli@unipi.it

Supporting Information

Table of contents

Synthetic procedures and NMR data of ILs **1-10** and **12-13** pages **S2-S9**

NMR spectra of ILs **1-10** and **12-13** pages **S10-S34**

TGA profiles of ILs **2-3, 5-6** and **8-9** pages **S35-S37**

DFT optimized geometries page **S38-S44**

Synthetic procedures and NMR data of ILs 1-10 and 12-13

General procedure for the preparation of thiol-functionalized carboxylate ILs 1-9. -The concentrations of commercial methylcarbonate ILs in methanol solution were determined by acid titration using a 1M HCl solution (pH Meter EUTECH pH 700, calibrated with three standard buffer solutions at pH 4.01, 7.00, and 10.00). Thiol-functionalized carboxylate ILs were prepared by adding an equimolar amount of either 3-mercaptopropionic acid (**3-MPA**) or *N*-acetyl-L-cysteine (**NAC**) to the commercial methylcarbonate IL (1-ethyl-3-methylimidazolium [EMIM], tributylmethylphosphonium [P_{4441}] or trioctylmethylphosphonium [P_{8881}]) methanol solution at room temperature. The reaction was performed under nitrogen atmosphere and all reagents were deoxygenated with a N_2 continuous flow before use (***method A:*** *anhydrous conditions, room temperature for 2h*) or without particular precautions under an air atmosphere (***method B:*** *standard conditions, room temperature for 2h*) or favouring the oxidation of the products by bubbling air inside the solution for the entire reaction (***method C:*** *air flow, room temperature for 6h*). In all cases, the mixture was concentrated at diminished pressure (50 °C) to remove methanol and the viscous liquid was dried under high vacuum and continuous stirring at 50 °C for 6h.

Preparation of 3-ethyl-1-methyl-1*H*-imidazolium 3-mercaptopropionate (1)

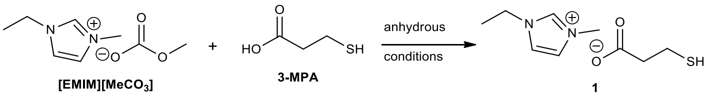

To a commercial methanolic solution of [EMIM]$MeCO_3$ (31.88% w/w, 13.8 g, 23.6 mmol) the commercial 3-mercaptopropionic acid (**3-MPA**, 1 equiv, 2.54 g, 23.6 mmol) was added and the reaction was performed according to the general procedure (Method A). Ionic liquid **1 (**5.10 g) was obtained as a pale-yellow viscous liquid in quantitative yield. ^{1}H NMR (CD_3OD, 250 MHz) δ: 9.06 (s, 1H, Im-H_2), 7.68, 7.60 (2m, each 1H, Im-H_4, Im-H_5), 4.30 (q, 2H, J_{vic} 7.3 Hz, CH_2N^+), 3.95 (s, 3H, CH_3N), 2.68 (t, 2H, J_{vic} 7.0 Hz, CH_2SH), 2.45 (t, 2H, J_{vic} 7.0 Hz, CH_2CO), 1.54 (t, 3H, J_{vic} 7.3 Hz, CH_3). ^{13}C NMR (CD_3OD, 62.9 MHz) δ: 179.7 (C=O), 137.7 (Im-C_2), 124.9, 123.2 (Im-C_4, Im-C_5), 45.9 (*C*H_2N^+), 43.3 (*C*H_2CO), 36.4 (*C*H_3N), 22.1 (*C*H_2SH), 15.6 (CH_3).

Preparation of 3-ethyl-1-methyl-1*H*-imidazolium 3-mercaptopropionate (1) and disulfide 2.

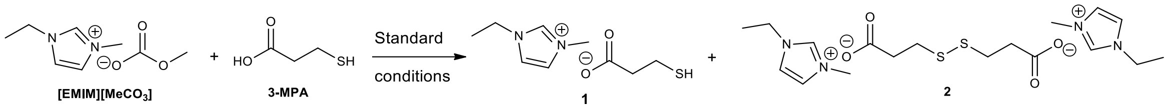

To a commercial methanolic solution of [EMIM]$MeCO_3$ (31.88% w/w, 13.8 g, 23.6 mmol), commercial 3-mercaptopropionic acid (**3-MPA**, 1 equiv, 2.54 g, 23.6 mmol) was added and the reaction was performed in accordance to the general procedure (Method B). The ^{1}H NMR spectrum (CD_3OD, 250.13 MHz) of the pale-yellow viscous liquid showed a mixture of thiol **1** and disulfide **2** in a 88:12 ratio, measured on the relative intensities of the CH_2S signals at δ 2.68 and 2.91, respectively. After 10 days the ^{1}H NMR spectrum showed a change in the mixture composition (^{1}H NMR, **1**:**2** 70:30 ratio), which changed again after 30 days (^{1}H NMR, **1**:**2** 10:90 ratio).

Preparation of imidazolium disulfide salt 2

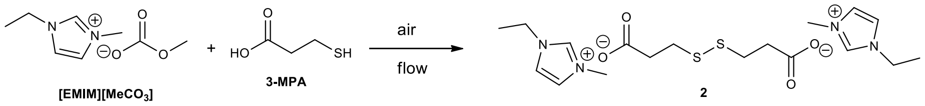

To a commercial methanolic solution of [EMIM]$MeCO_3$ (31.88% w/w, 13.8 g, 23.6 mmol), commercial 3-mercaptopropionic acid (**3-MPA**, 1 equiv, 2.54 g, 23.6 mmol) was added and the reaction was performed according to the general procedure (Method C). Ionic liquid **2** (5.08 g) was obtained as a pale-yellow viscous liquid in quantitative yield. ^{1}H NMR (CD_3OD, 250.13 MHz) δ: 9.01 (bs, 2H, 2×Im-H_2), 7.68, 7.61 (2bs, each 2H, 2×Im-H_4, 2×Im-H_5), 4.30 (q, 4H, J_{vic} 7.3 Hz, 2×CH_2N^+), 3.96 (s, 6H, 2×CH_3N), 2.91 (t, 4H, J_{vic} 7.2 Hz, 2×CH_2SS), 2.52 (t, 4H, J_{vic} 7.2 Hz, 2×CH_2CO), 1.55 (t, 6H, J_{vic} 7.3 Hz, 2×CH_3). ^{13}C NMR (CD_3OD, 62.9 MHz) δ: 179.4 (2×*C*=O), 137.7 (2×Im-C_2), 124.9, 123.3 (2×Im-C_4, 2×Im-C_5), 45.9 (2×*C*H_2N^+), 38.7 (2×*C*H_2CO), 36.5 (2×*C*H_3N), 36.4 (2×*C*H_2SS), 15.7 (2×*C*H_3). Anal. Calcd for $C_{18}H_{30}N_4O_4S_2$: C, 50.21; H, 7.02; N, 13.01. Found: C, 50.18; H, 7.05; N, 13.10.

Preparation of 3-ethyl-1-methyl-1*H*-imidazolium *N*-acetyl-L-cysteinate (3)

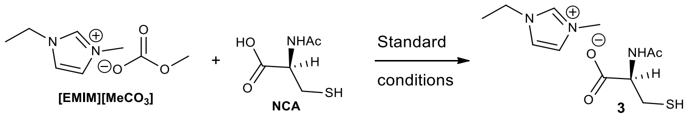

To a commercial methanolic solution of [EMIM]$MeCO_3$ (58.74% w/w, 2.19 g, 3.89 mmol), commercial *N*-acetyl-L-Cysteine (**NCA**, 1 equiv, 0.640 g, 3.89 mmol) was added and the reaction was performed according to the general procedure (Method B). Ionic liquid **3** (1.01 g, 95% yield) was obtained as a yellow viscous liquid. ^{1}H NMR (CD_3OD, 250.13 MHz) δ: 9.10 (s, 1H, Im-H2), 7.65, 7.59 (2m, each 1H, Im-H4, Im-H5), 4.39 (bt, 1H, J_{vic} 7.4 Hz, C*H*N), 4.26 (q, 2H, J_{vic} 7.5 Hz, C*H*2N$^+$), 3.94 (s, 3H, C*H*3N), 2.94 (dd, 1H, J_{vic} 5.0 Hz, J_{gem} 12.5 Hz, C*H*2SH), 2.87 (dd, 1H, C*H*2SH), 2.02 (s, 3H, C*H*3CON), 1.53 (t, 3H, J_{vic} 7.4 Hz, C*H*3). ^{13}C NMR (CD_3OD, 62.9 MHz) δ: 176.2 (*C*OO), 172.6 (*C*ON) 137.7 (Im-*C*2), 125.0, 123.3 (Im-*C*4, Im-*C*5), 57.9 (*C*HN), 46.0 (*C*H2N$^+$), 36.4 (*C*H3N), 28.0 (*C*H2SH), 22.8 (*C*H3CON), 15.6 (*C*H3).

Preparation of tributylmethylphosphonium 3-mercaptopropionate (4)

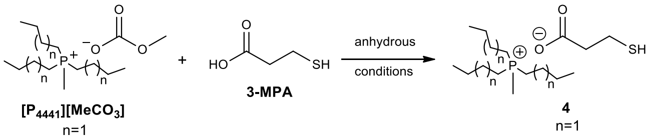

To a commercial methanolic solution of [P_{4441}]$MeCO_3$ (32.00% w/w, 2.30 g, 2.52 mmol), commercial 3-mercaptopropionic acid (**3-MPA**, 1 equiv, 270 mg, 2.52 mmol) was added and the reaction was performed in accordance to the general procedure (Method A). Ionic liquid **4** (800 mg, 99% yield) was obtained as a yellow viscous hygroscopic liquid. The ^{1}H NMR spectrum showed that thiol **4** was impure of disulfide **5** (7%). ^{1}H NMR ($CDCl_3$, 250.13 MHz) of thiol **4** δ: 2.68 (t, 2H, J_{vic} 7.0 Hz, C*H*2SH), 2.39 (t, 2H, J_{vic} 7.0 Hz, C*H*2CO), 2.34-2.17 (m, 6H, 3×C*H*2P$^+$), 1.95 (d, 3H, $J_{H,P}$ 13.5 Hz, C*H*3P$^+$), 1.55-1.33 (m, 12H, 3×C*H*2C*H*2), 0.92 (bt, 9H, J_{vic} 6.8 Hz, 3×C*H*3). ^{13}C NMR ($CDCl_3$, 62.9 MHz) δ: 176.4 (*C*=O), 42.9 (*C*H2CO), 23.8-23.4 (3×*C*H2*C*H2), 22.1 (*C*H2SH), 20.0-19.2 (3×*C*H2P$^+$), 13.3 (3×*C*H3), 4.60, 3.77 (*C*H3P$^+$).

Preparation of tributylmethylphosphonium 3-mercaptopropionate (4) and disulfide 5.

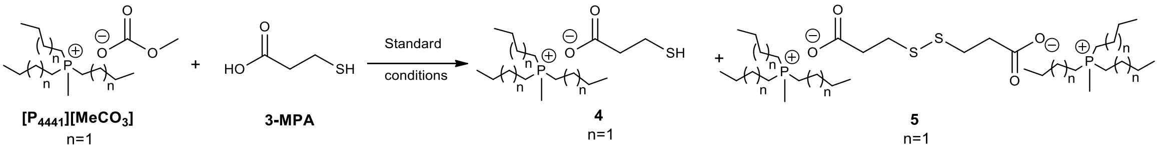

To a commercial methanolic solution of [P_{4441}]$MeCO_3$ (32.00% w/w, 2.30 g, 2.52 mmol), commercial 3-mercaptopropionic acid (**3-MPA**, 1 equiv, 270 mg, 2.52 mmol) was added and the reaction was performed according to the general procedure (Method B). The ^{1}H NMR spectrum ($CDCl_3$, 250.13 MHz) of the yellow viscous liquid showed a mixture of thiol **4** and disulfide **5** in a 40:60 ratio, measured on the relative intensities of the C*H*2S signals at δ 2.68

and 2.91 respectively. After 60 days (storage under air), NMR analyses (^{1}H and ^{13}C) showed the exclusive presence of disulphide **5**.

Preparation of tributylmethylphosphonium disulfide salt 5.

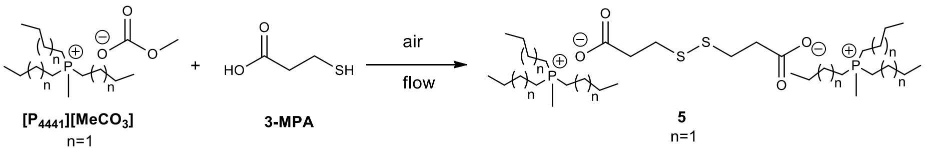

To a commercial methanolic solution of [P_{4441}]$MeCO_3$ (32.00% w/w, 2.30 g, 2.52 mmol), commercial 3-mercaptopropionic acid (**3-MPA**, 1 equiv, 270 mg, 2.52 mmol) was added and the reaction was performed in accordance to the general procedure (Method C). Ionic liquid **5** (801 mg) was obtained as a yellow viscous liquid in quantitative yield. ^{1}H NMR (CD_3OD, 400 MHz) δ: 2.92 (t, 4H, J_{vic} 7.3 Hz, 2×CH_2SS), 2.55 (t, 4H, J_{vic} 7.3 Hz, 2×CH_2CO), 2.23-2.15 (m, 12H, 6×C$H_2$$P^+$), 1.81 (d, 6H, $J_{H,P}$ 13.7 Hz, 2×C$H_3$$P^+$), 1.60-1.49 (m, 24H, 6×CH_2CH_2), 1.00 (t, 18H, J_{vic} 7.1 Hz, 6×CH_3). ^{13}C NMR ($CDCl_3$, 62.9 MHz) δ: 176.4 (2×*C*=O), 38.4 (2×*C*H_2CO), 36.8 (2×*C*H_2SS), 23.8-23.4 (6×*C*H_2*C*H_2), 20.0, 19.3 (6×*C*$H_2$$P^+$), 13.4 (6×*C*$H_3$), 4.61, 3.78 (2×*C*$H_3$$P^+$). Anal. Calcd for $C_{32}H_{68}O_4P_2S_2$: C, 59.78; H, 10.66. Found: C, 59.81; H, 10.70.

Preparation of tributylmethylphosphonium *N*-acetyl-L-cysteinate (6)

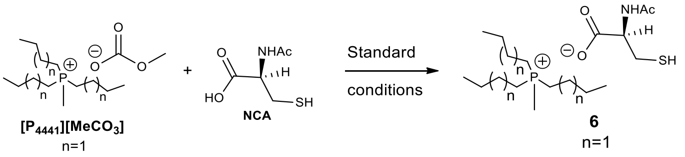

To a commercial methanolic solution of [P_{4441}]$MeCO_3$ (32.00 % w/w, 2.41 g, 2.64 mmol), commercial *N*-acetyl-L-cysteine (**NCA**, 1 equiv, 435.4 mg, 2.64 mmol) was added and the reaction was performed according to the general procedure (Method B). Ionic liquid **6** (980 mg. 98% yield) was obtained as a yellow viscous hygroscopic liquid. ^{1}H NMR ($CDCl_3$, 250.13 MHz) δ: 7.05 (d, 1H, $J_{NH,H}$ 5.0 Hz, N*H*Ac), 4.27 (m, 1H, C*H*N), 3.00 (m, 2H, CH_2SH), 2.10-2.32 (m, 6H, 3×C$H_2$$P^+$), 1.93 (s, 3H, CH_3CON), 1.91 (d, 3H, $J_{H,P}$ 13.0 Hz, C$H_3$$P^+$), 1.70-1.25 (m, 12H, 3×CH_2CH_2), 0.96-0.82 (m, 9H, 3×CH_3). ^{13}C NMR ($CDCl_3$, 62.9 MHz) δ: 172.8 (COO), 169.6 (*C*ON), 55.9 (*C*HN), 27.4 (*C*H_2SH), 23.8 (*C*H_3CON), 23.5-23.3 (3×*C*H_2*C*H_2), 20.1, 19.3 (3×*C*$H_2$$P^+$), 13.3 (3×*C*$H_3$), 4.60, 3.80 ($CH_3P^+$).

Preparation of trioctylmethylphosphonium 3-mercaptopropionate (7)

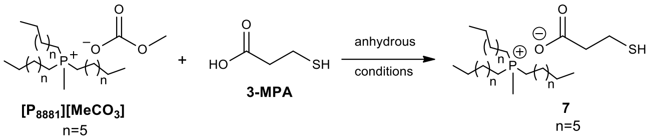

To a commercial methanolic solution of [P_{8881}]$MeCO_3$ (60.31 % w/w, 1.51 g, 1.98 mmol), commercial 3-mercaptopropionic acid (**3-MPA**, 1 equiv, 212.3 mg, 1.98 mmol) was added and the reaction was performed according to the general procedure (Method A). Ionic liquid **7** (970 mg) was obtained as a yellow viscous liquid in quantitative yield. The ^{1}H NMR spectrum ($CDCl_3$, 400 MHz) showed the presence of thiol **7** and disulfide **8** (12%). ^{1}H NMR ($CDCl_3$, 400 MHz) of thiol **7** δ: 2.72 (t, 2H, J_{vic} 7.4 Hz, CH_2SH), 2.45 (m, 2H, CH_2CO), 2.36-2.28 (m, 6H, 3×C$H_2$$P^+$), 2.02 (d, 3H, $J_{H,P}$ 13.5 Hz, C$H_3$$P^+$), 1.50-1.38 and 1.26-1.21 (2m, 36H, 18CH_2), 0.82 (bt, 9H, 3×CH_3). ^{13}C NMR ($CDCl_3$, 100 MHz) δ: 176.0 (*C*=O), 42.8 (*C*H_2CO), 31.5, 30.6, 30.5, 28.8, 22.4, 21.6, 21.5 (18×*C*H_2), 22.0 (*C*H_2SH), 20.2, 19.7 (3×*C*$H_2$$P^+$), 13.9 (3×*C*$H_3$), 4.58, 4.05 (*C*$H_3$$P^+$).

Preparation of trioctylmethylphosphonium 3-mercaptopropionate (7) and disulfide 8.

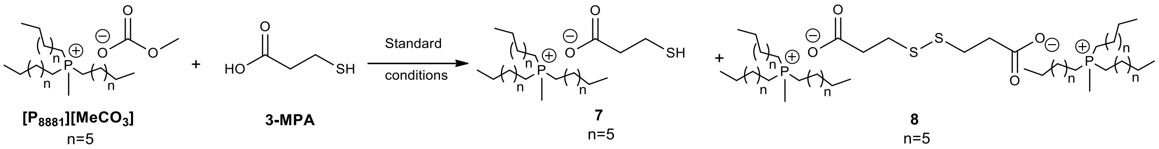

To a commercial methanolic solution of [P_{8881}]$MeCO_3$ (58.74% w/w, 1.60 g, 2.04 mmol), commercial 3-mercaptopropionic acid (**3-MPA**, 1 equiv, 219 mg, 2.04 mmol) was added and the reaction was performed according to the general procedure (Method B). The ^{1}H NMR spectrum ($CDCl_3$, 250.13 MHz) of the yellow viscous liquid (1.01 g) showed a mixture of thiol **7** and disulfide **8** in a 79:21 ratio, measured on the relative intensities of the CH_2S signals at δ 2.56 and 2.78 respectively.

Preparation of trioctylmethylphosphonium disulfide salt 8.

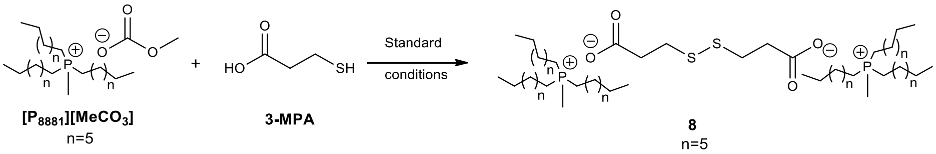

To a commercial methanolic solution of [P_{8881}]$MeCO_3$ (60.31 % w/w, 1.51 g, 1.98 mmol), commercial 3-mercaptopropionic acid (**3-MPA**, 1 equiv, 212.3 mg, 1.98 mmol) was added

and the reaction was performed according to the general procedure (Method C). Ionic liquid **8** (969 mg) was obtained as a yellow viscous liquid in quantitative yield. ^{1}H NMR (CD_3OD, 400 MHz) δ: 2.93 (t, 4H, J_{vic} 7.3 Hz, 2×CH_2S), 2.57 (t, 4H, J_{vic} 7.3 Hz, 2×CH_2CO), 2.23-2.16 (m, 12H, 6×CH_2P$^+$), 1.81 (d, 6H, $J_{H,P}$ 13.6 Hz, 2×CH_3P$^+$), 1.62-1.63 and 1.1.42-1.28 (2m, 72H, 36×CH_2), 0.91 (t, 18H, J_{vic} 7.0 Hz, 6×CH_3); ^{13}C NMR (CD_3OD, 100 MHz) δ: 178.9 (*C*=O), 38.2 (2×*C*H_2CO), 36.1 (2×*C*H_2SS), 32.9, 31.8, 31.7, 30.1, 29.9, 23.7, 22.4, 22.3, (36×*C*H_2), 21.1, 20.6 (6×*C*H_2P$^+$), 14.4 (6×*C*H_3), 4.10, 4.58 (2×*C*H_3P$^+$). Anal. Calcd for $C_{56}H_{116}O_4P_2S_2$: C, 68.66; H, 11.94. Found: C, 68.70; H, 11.98.

Preparation of trioctylmethylphosphonium *N*-acetyl-L-cysteinate (9)

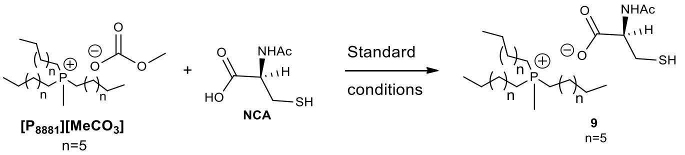

To a commercial methanolic solution of [P_{8881}]$MeCO_3$ (60.31 % w/w, 1.40 g, 1.84 mmol), commercial *N*-acetyl-L-Cysteine (**NCA**, 1 equiv, 303 mg, 1.84 mmol) was added and the reaction was performed according to the general procedure (Method B). Ionic liquid **9** (1.00 g, 99% yield) was obtained as a viscous hygroscopic liquid. ^{1}H NMR ($CDCl_3$, 250.13 MHz) δ: 7.14 (d, 1H, $J_{NH,H}$ 6.5 Hz, N*H*Ac), 4.35 (m, 1H, C*H*N), 3.43 (dd, 1H, J_{vic} 4.6 Hz, J_{gem} 12.3 Hz, CH_2SH), 3.28 (dd, 2H, J_{vic} 5.5 Hz, CH_2SH), 2.37-2.21 (m, 6H, 3×CH_2P$^+$), 2.02 (d, 3H, $J_{H,P}$ 13.6 Hz, CH_3P$^+$), 1.98 (s, 3H, CH_3CON), 1.60-1.40 and 1.38-1.20 (2m, 36H, 18CH_2), 0.88 (bt, 9H, 3×CH_3); ^{13}C NMR ($CDCl_3$, 62.9 MHz) δ: 172.6 (*C*OO), 169.4 (*C*ON), 55.9 (*C*HN), 27.4 (*C*H_2SH), 23.4 (*C*H_3CON), 31.5, 30.6, 30.4, 28.7, 22.4, 21.5 (18×CH_2), 20.3-19.5 (3×*C*H_2P$^+$), 13.9 (3×*C*H_3), 4.63, 3.80 (*C*H_3P$^+$).

Heating treatment of imidazolium disulfide salt 2.

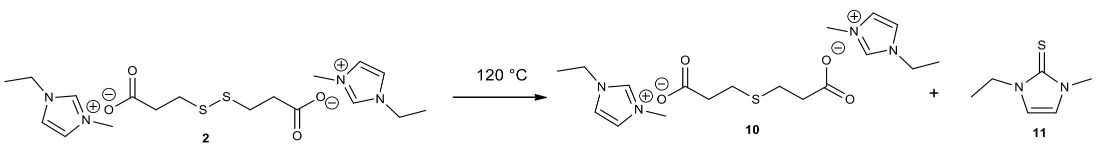

Imidazolium disulfide salt **2** (230 mg) was heated to 120 °C degrees for 4h. The reaction was cooled down to room temperature and the ^{1}H NMR analysis (CD_3OD, 250.13 MHz) of viscous liquid showed a mixture of **11** and **10** in a 45:55 ratio, measured on the relative intensities of the CH_3N signals at δ 3.59 and 3.91 respectively. The syrup was partitioned between H_2O (10 mL) and Et_2O (10 mL), the phases were separated, and the aqueous layer was washed with Et_2O (3×10 mL). The combined organic extracts were dried and

concentrated at diminished pressure to give a solid constituted (NMR) exclusively by an imidazole 2-thione (**11)** (30 mg). The aqueous layer was concentrated at diminished pressure to give **10** (150 mg) as a viscous liquid.

The 2H-imidazole-thione (**11**) was a solid, ^{1}H NMR (CD_3OD, 250.13 MHz) δ: 6.69 (s, 2H, Im-H_4, Im-H_5), 4.05 (q, 2H, J_{vic} 7.2 Hz, CH_2N^+), 3.59 (s, 3H, CH_3N), 1.34 (t, 6H, J_{vic} 7.2 Hz, CH_3).^{13}C NMR (CD_3OD, 62.9 MHz) δ: 161.4 (*C*=S), 117.8, 115.8 (Im-C_4, Im-C_5), 42.9 (CH_2N^+), 34.9 (CH_3N), 14.2 (CH_3). NMR data (^{1}H and ^{13}C) agreed with those reported in the literature [1].

Sulfide ionic liquid **10** was a viscous yellow liquid, ^{1}H NMR (CD_3OD, 400 MHz) δ: 9.01 (bs, 2H, 2×Im-H_2), 7.65, 7.57 (2bt, each 2H, 2×Im-H_4, 2×Im-H_5), 4.27 (q, 4H, J_{vic} 7.3 Hz, 2×CH_2N^+), 3.94 (s, 6H, 2×CH_3N), 2.77 (t, 4H, J_{vic} 7.6 Hz, 2×CH_2S), 2.50 (t, 4H, J_{vic} 7.6 Hz, 2×CH_2CO), 1.53 (t, 6H, J_{vic} 7.3 Hz, 2×CH_3).^{13}C NMR (CD_3OD, 100 MHz) δ: 177.8 (2×*C*=O), 137.7 (2×Im-C_2), 125.0, 123.3 (2×Im-C_4, 2×Im-C_5), 46.0 (2×CH_2N^+), 37.5 (2×CH_2CO), 36.5 (2×CH_3N), 28.7 (2×CH_2S), 15.6 (2×CH_3). Anal. Calcd for $C_{18}H_{30}N_4O_4S$: C, 54.25; H, 7.59; N, 14.06. Found: C, 54.21; H, 7.57; N, 14.11.

Heating treatment of 3-ethyl-1-methyl-1*H*-imidazolium *N*-acetyl-L-cysteinate (3)

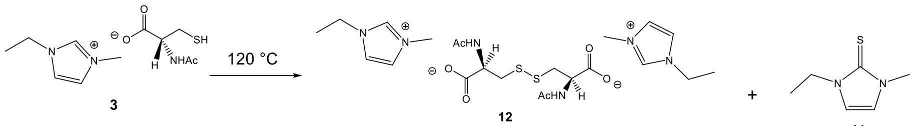

Compound **3** (100 mg, 0.366 mmol) was heated to 120 ℃ degrees for 4h. The reaction was cooled down to room temperature and the ^{1}H NMR analysis (CD_3OD, 250.13 MHz) of the viscous liquid showed a mixture of **11** and disulfide **12** in a 12:88 ratio, based on integral values of CH_3N proton signals at δ 3.59 and 3.92 respectively. The viscous liquid was washed with Et_2O (3×10 mL) and the aqueous layer was concentrated under diminished pressure to give a yellow viscous liquid constituted exclusively by disulfide ionic liquid **12** (78 mg). ^{1}H NMR (CD_3OD, 250.13 MHz) δ: 9.01 (bs, 2H, 2×Im-H_2), 7.65, 7.58 (2bs, each 2H, 2×Im-H_4, 2×Im-H_5), 4.46 (m, 2H, 2×C*H*N), 4.28 (q, 4H, J_{vic} 7.5 Hz, 2×CH_2N^+), 3.92 (s, 6H, 2×CH_3N), 3.30 (m, 2H, CH_2S), 3.00 (dd, 2H, J_{gem} 12.5 Hz, J_{vic} 7.5 Hz, CH_2S), 2.00 (s, 3H, 2×CH_3CON), 1.53 (t, 6H, J_{vic} 7.5 Hz, 2×CH_3); ^{13}C NMR (CD_3OD, 62,9 MHz) δ: 175.0 (2×COO^-), 172.8 (2×*C*ON), 137.7 (2×Im-C_2), 125.0, 123.3 (2×Im-C_4, 2×Im-C_5), 46.0 (2×CH_2N^+), 55.9 (2×*C*HN), 43.0 (2×CH_2S), 36.5 (2×CH_3N), 22.9 (2×CH_3CON), 15.6

(2×CH_3). Anal. Calcd for $C_{22}H_{36}N_6O_6S_2$: C, 48.51; H, 6.66; N, 15.43. Found: C, 48.55; H, 6.71; N, 15.48.

Heating treatment of tributylmethylphosphonium disulfide salt 5.

Tributylmethylphosphonium disulphide salt **5** (50 mg) was heated to 120 ℃ degrees for 4h. The reaction was cooled down to room temperature and the viscous liquid (49 mg) was constituted (NMR analysis, $CDCl_3$, 250.13 MHz) exclusively by disulfide salt **5**.

Heating treatment of tributylmethylphosphonium *N*-acetyl-L-cysteinate (6)

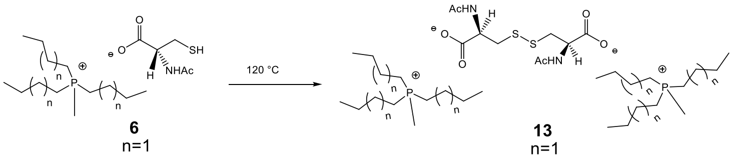

Compound **6** (100 mg, 0.366 mmol) was heated to 120 ℃ degrees for 4h. The reaction was cooled down to room temperature and the pale-yellow viscous liquid (95 mg) was constituted (NMR analysis, $CDCl_3$, 250.13 MHz) exclusively by the disulfide **13**. ^{1}H NMR ($CDCl_3$, 250.13 MHz) δ: 7.30 (d, 2H, $J_{NH,H}$ 6.9 Hz, 2×N*H*Ac), 4.32 (m, 1H, 2×C*H*N), 3.35 (dd, 2H, J_{vic} 4.2 Hz, J_{gem} 13.2 Hz, CH_2S), 3.13 (dd, 2H, J_{vic} 6.2 Hz, CH_2S), 2.31-2.10 (m, 12H, 6×C$H_2$$P^+$), 1.93 (t, 3H, 2×C$H_3$CON), 1.91 (d, 3H, $J_{H,P}$ 13.0 Hz, 2×C$H_3$$P^+$), 1.46-1.38 (m, 24H, 6×CH_2CH_2), 0.89 (bt, 18H, J_{vic} 7.0 Hz, 6×CH_3); ^{13}C NMR ($CDCl_3$, 62.9 MHz) δ: 173.7 (2×*C*OO), 169.9 (2×*C*ON), 54.2 (2×*C*HN), 43.5 (2×*C*H_2S), 23.8 (2×*C*H_3CON), 23.5-23.3 (6×CH_2*C*H_2), 20.0, 19.2 (6×CH_2P^+), 13.3 (6×CH_3), 4.62, 3.79 (2×CH_3P^+). Anal. Calcd for $C_{36}H_{74}N_2O_6P_2S_2$: C, 57.11; H, 9.85; N, 3.70. Found: C, 57.15; H, 9.89; N, 3.76.

References

[1] Tao, X.-L., Mei, M., Wang, Y.-G. *Synthetic Comm.* **2007**, *37*, 399-408.

NMR spectra of ILs 1-10 and 12-13

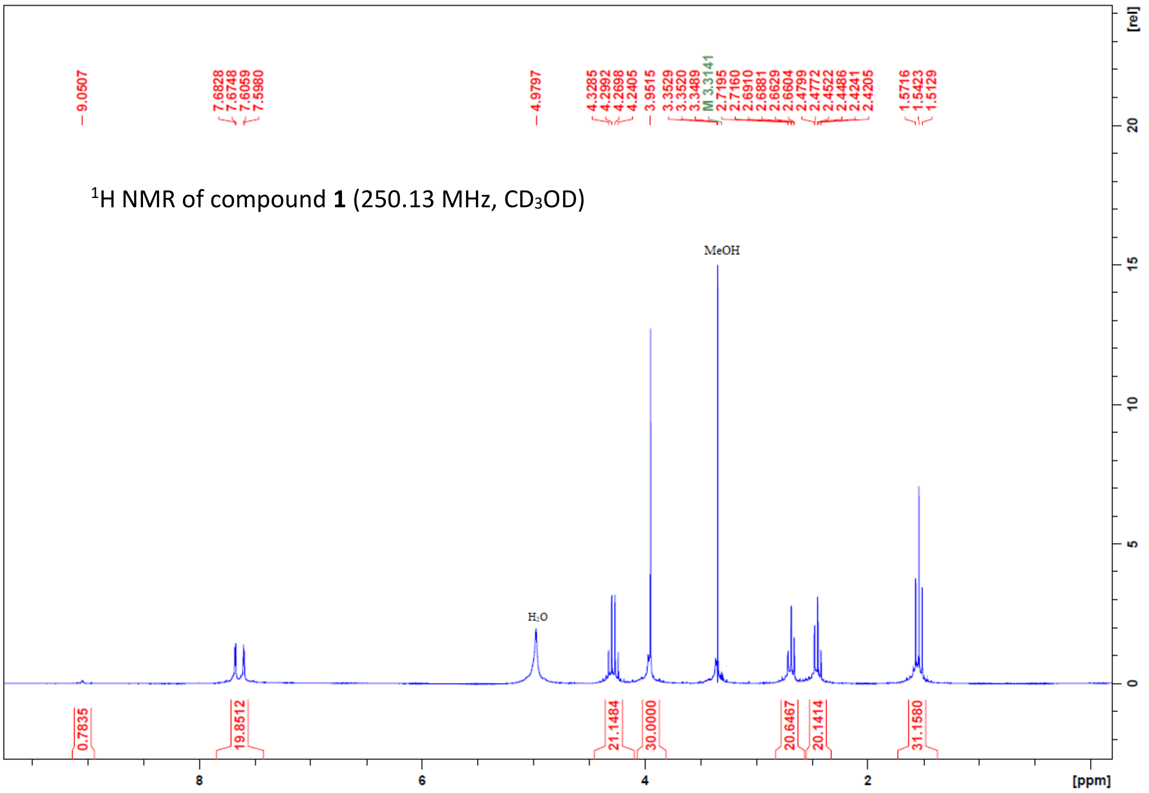

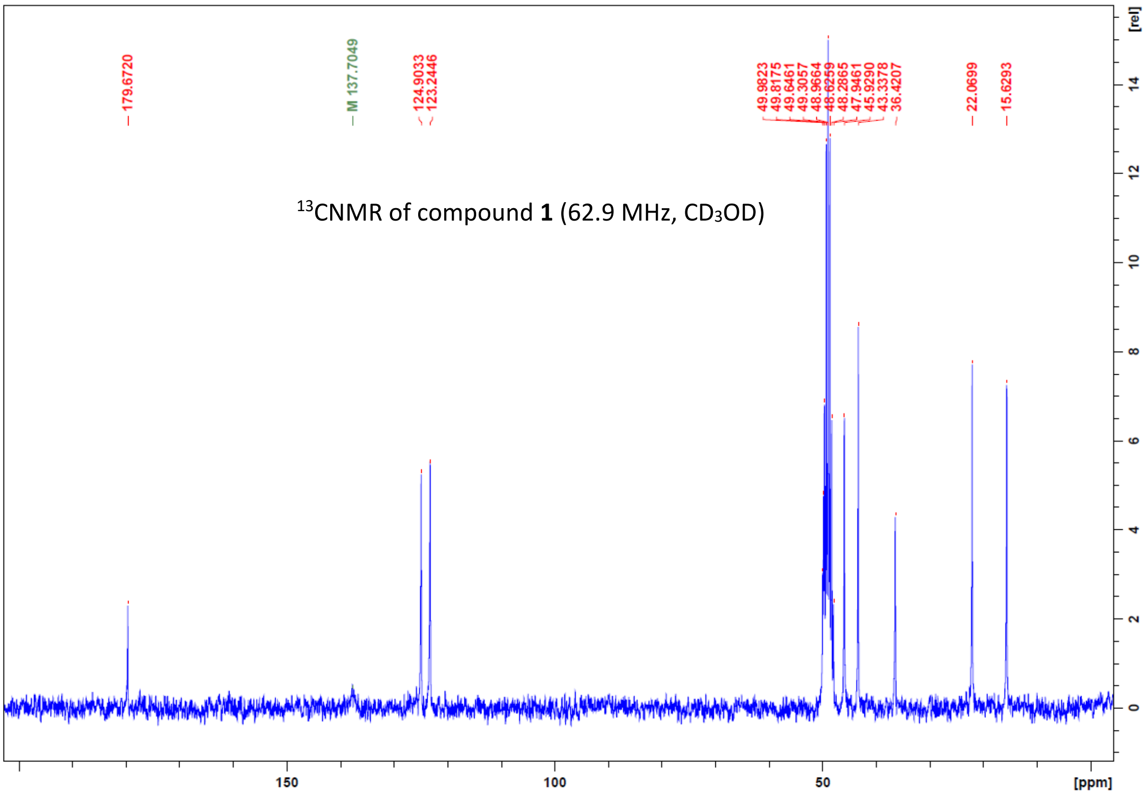

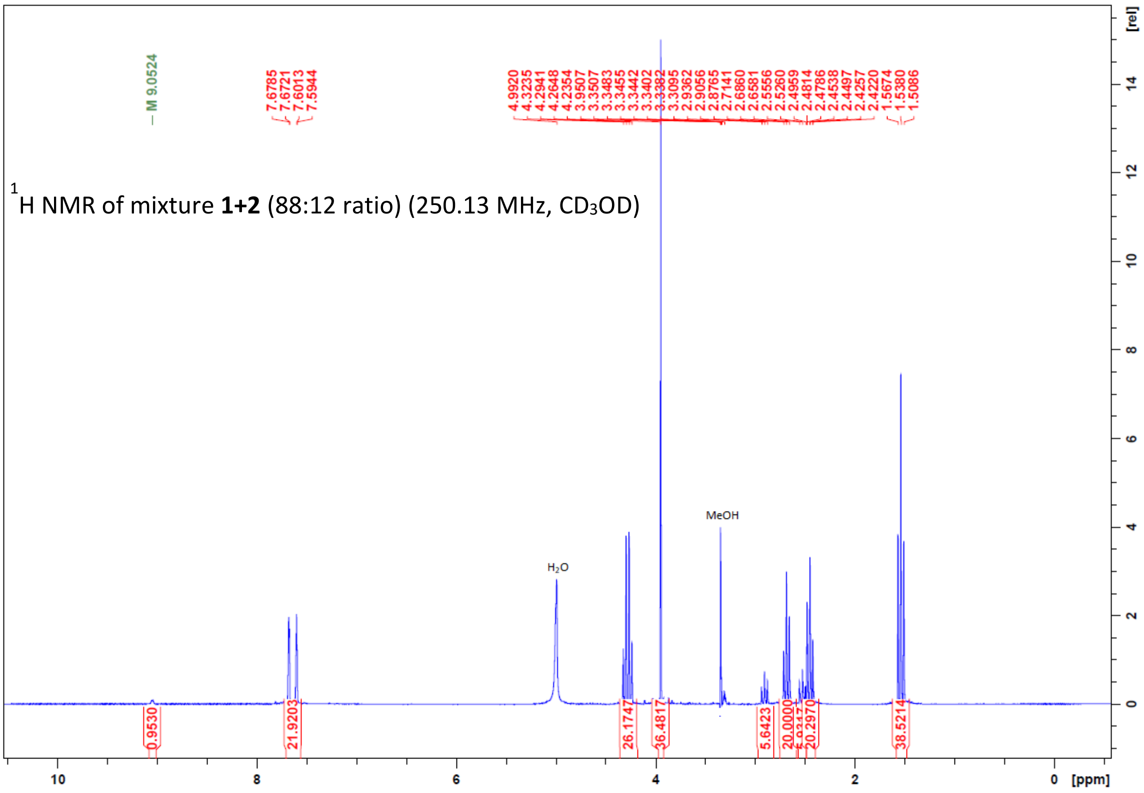

^{1}H NMR of mixture **1+2** (88:12 ratio) (250.13 MHz, CD_3OD)

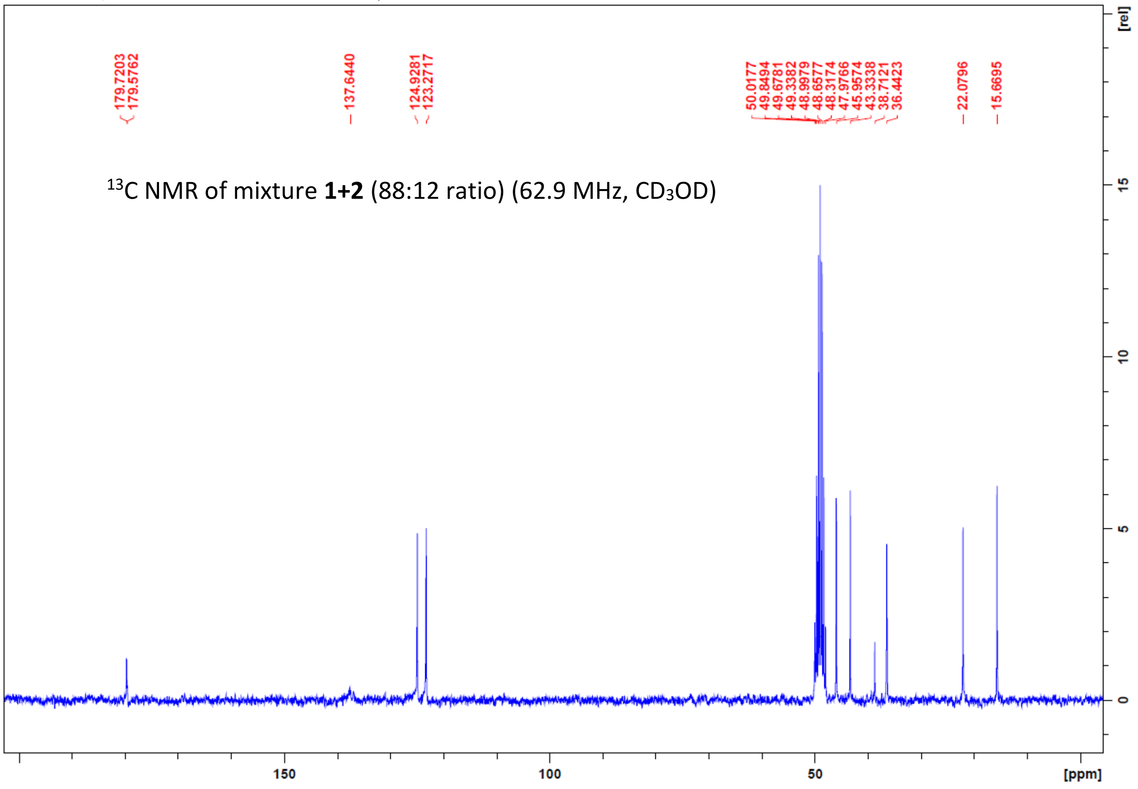

^{13}C NMR of mixture **1+2** (88:12 ratio) (62.9 MHz, CD_3OD)

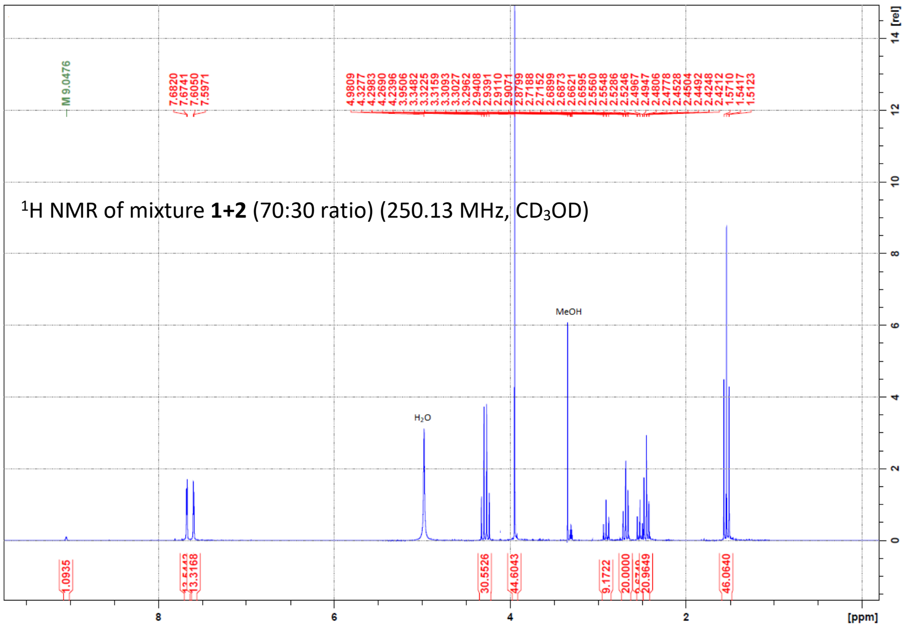
1H NMR of mixture 1+2 (70:30 ratio) (250.13 MHz, CD3OD)
H2O
MeOH
[ppm]
[rel]

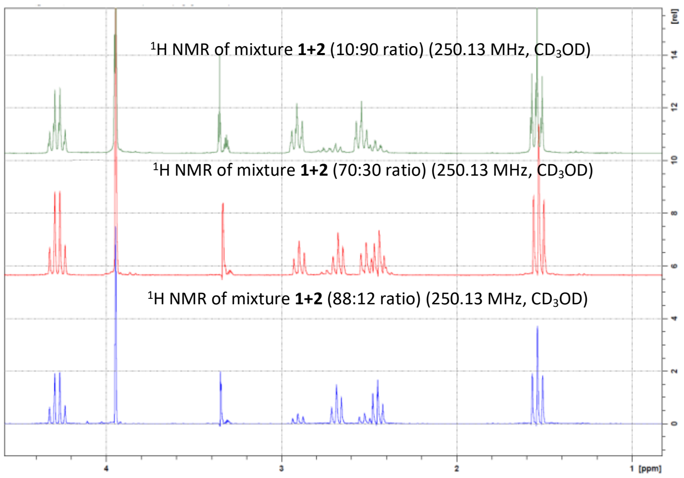
1H NMR of mixture 1+2 (10:90 ratio) (250.13 MHz, CD3OD)
1H NMR of mixture 1+2 (70:30 ratio) (250.13 MHz, CD3OD)
1H NMR of mixture 1+2 (88:12 ratio) (250.13 MHz, CD3OD)
[ppm]
[rel]

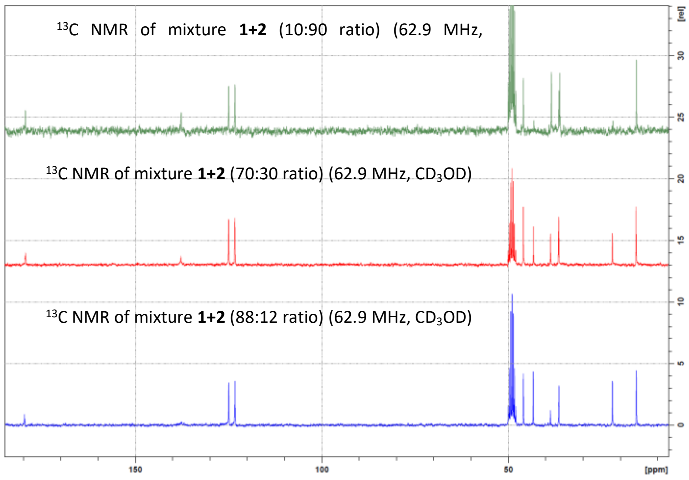
13C NMR of mixture 1+2 (10:90 ratio) (62.9 MHz,
13C NMR of mixture 1+2 (70:30 ratio) (62.9 MHz, CD3OD)
13C NMR of mixture 1+2 (88:12 ratio) (62.9 MHz, CD3OD)
[ppm]
[rel]

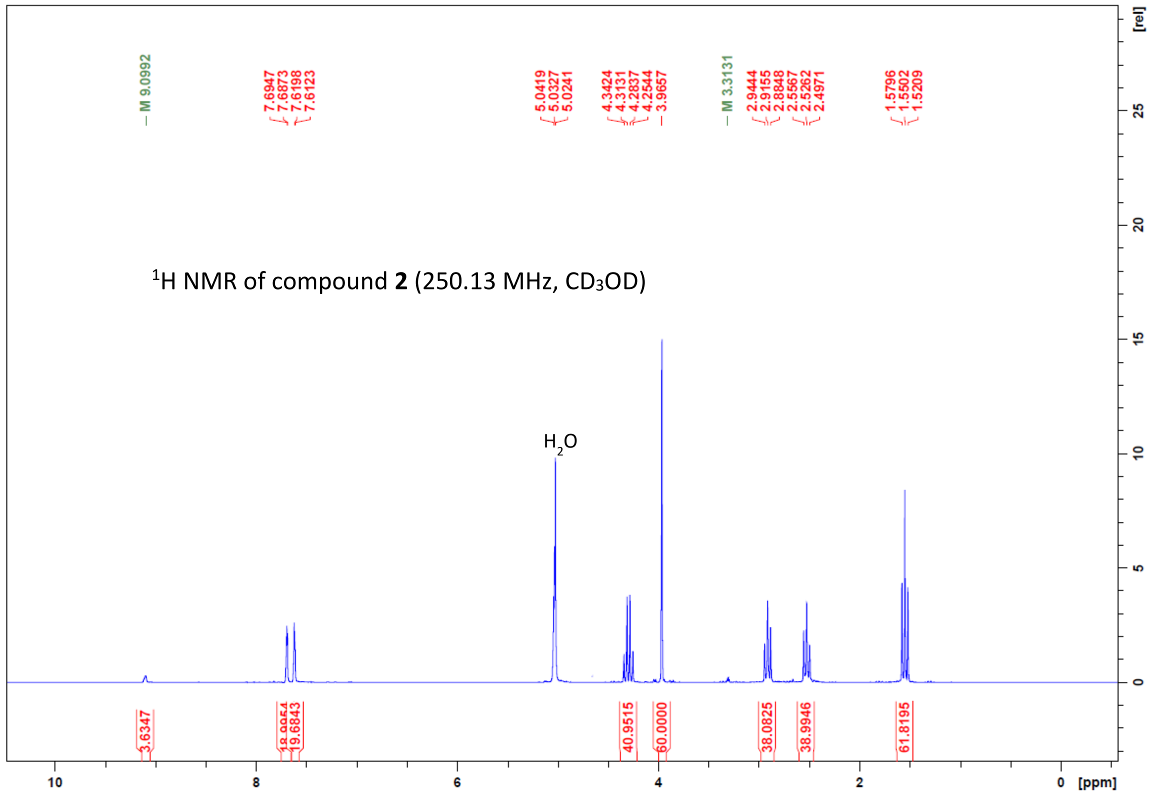
1H NMR of compound 2 (250.13 MHz, CD3OD)
H2O
[rel]
[ppm]

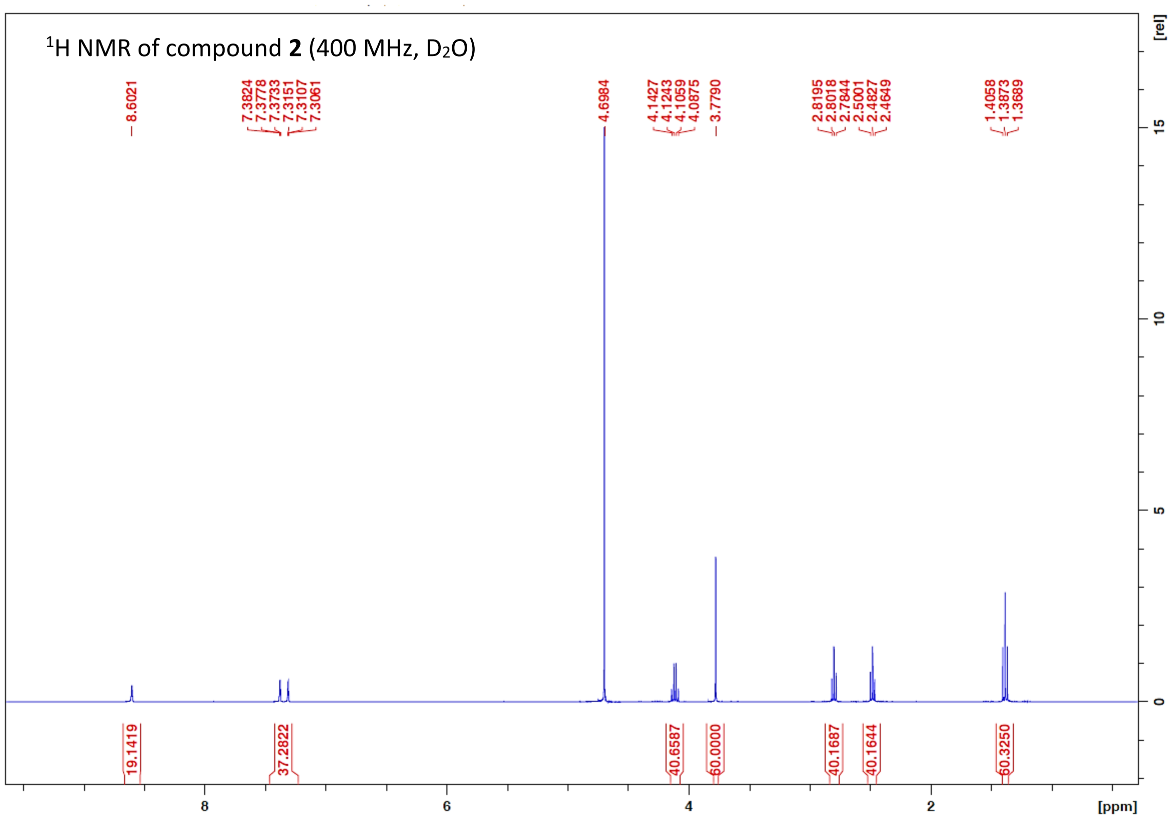
1H NMR of compound 2 (400 MHz, D2O)
[rel]
[ppm]

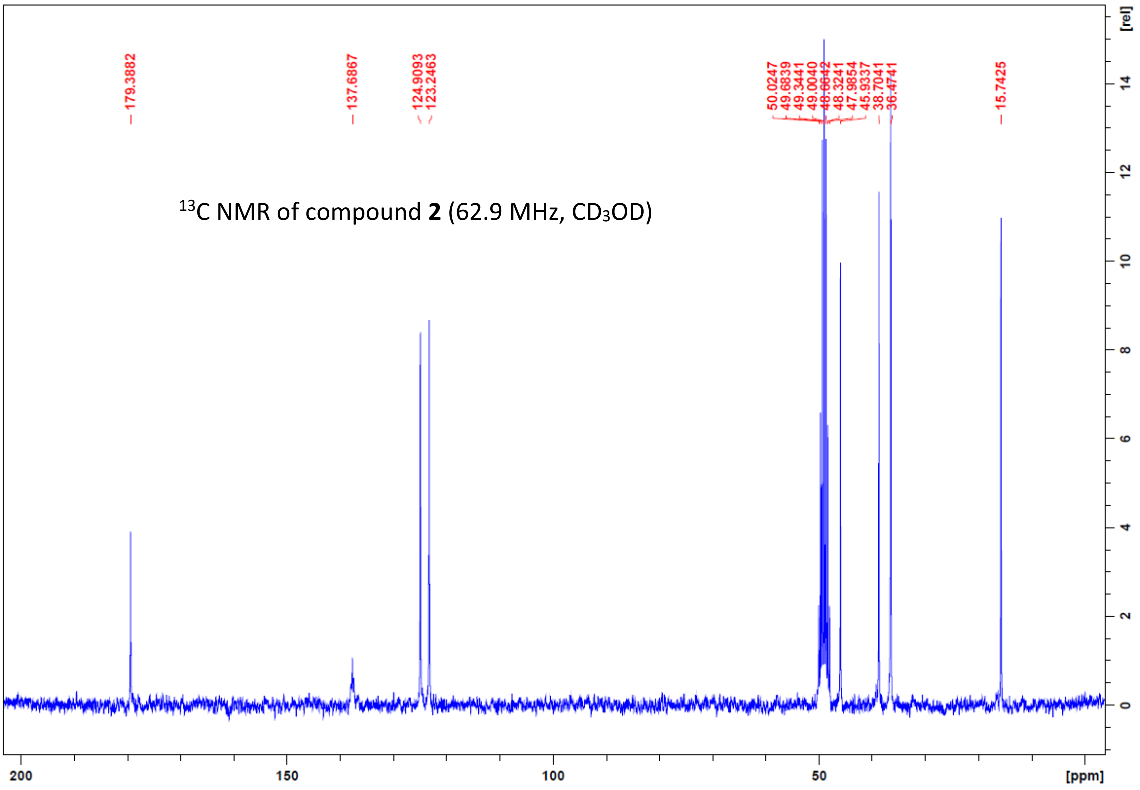
179.3882
137.6867
124.9093
123.2463
50.0247
49.6839
49.3441
49.0040
48.6642
48.3241
47.9854
45.9337
38.7041
36.4741
15.7425
^{13}C NMR of compound **2** (62.9 MHz, CD_3OD)
[rel]
[ppm]

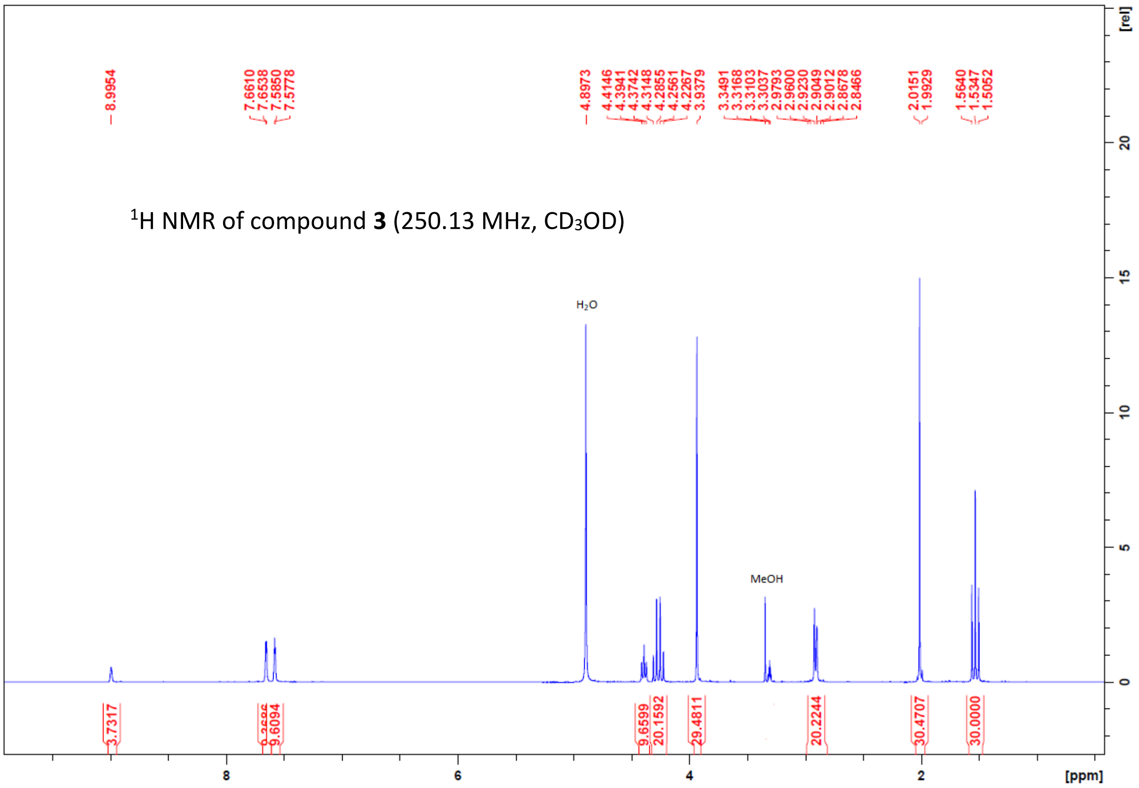
8.9954
7.6610
7.6538
7.5850
7.5778
4.8973
4.4146
4.3941
4.3742
4.3148
4.2855
4.2561
4.2267
3.9379
3.3491
3.3168
3.3103
3.3037
2.9793
2.9600
2.9230
2.9049
2.9012
2.8678
2.8466
2.0151
1.9929
1.5640
1.5347
1.5052
^{1}H NMR of compound **3** (250.13 MHz, CD_3OD)
H_2O
MeOH
3.7317
9.6094
9.6599
20.1592
29.4811
20.2244
30.4707
30.0000
[rel]
[ppm]

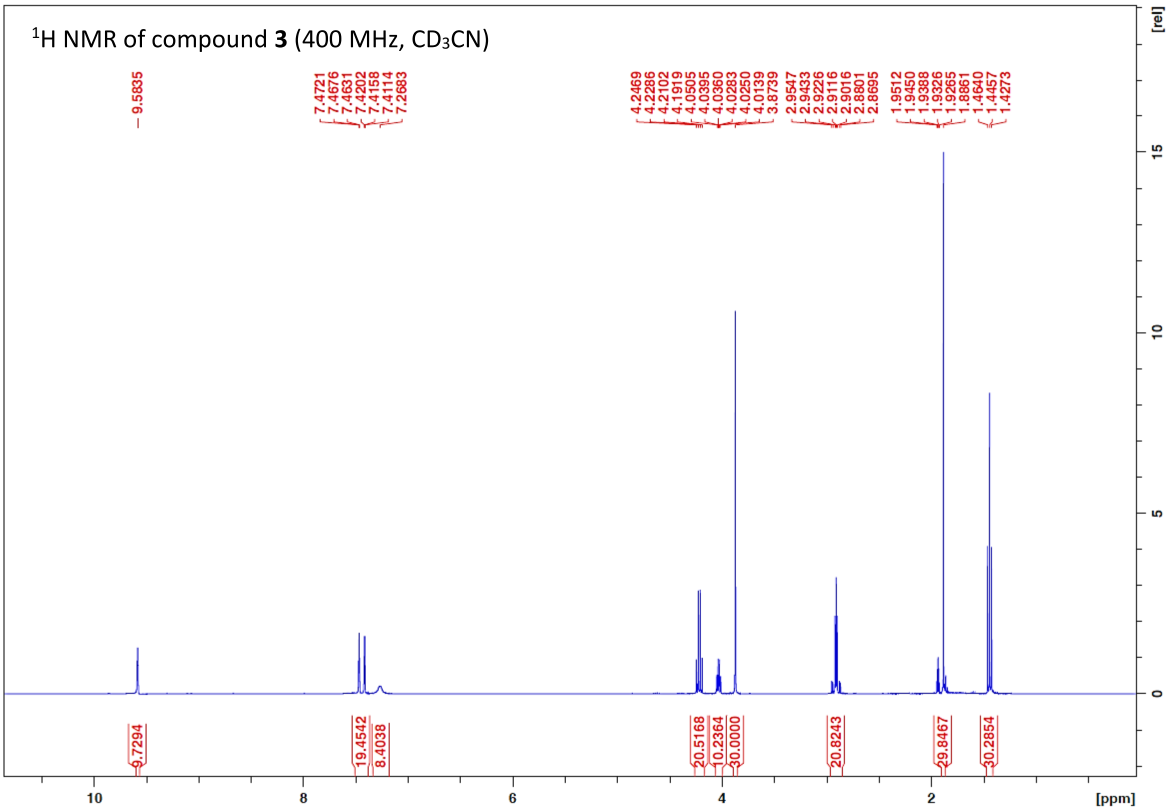
^{1}H NMR of compound **3** (400 MHz, CD_3CN)
9.5835
7.4721 7.4676 7.4631 7.4202 7.4158 7.4114 7.2683
4.2469 4.2286 4.2102 4.1919 4.0505 4.0395 4.0360 4.0283 4.0250 4.0139 3.8739
2.9547 2.9433 2.9226 2.9116 2.9016 2.8801 2.8695
1.9512 1.9450 1.9388 1.9326 1.9265 1.8861 1.4640 1.4457 1.4273
9.7294 19.4542 8.4038 20.5168 10.2364 30.0000 20.8243 29.8467 30.2854
[rel]
[ppm]

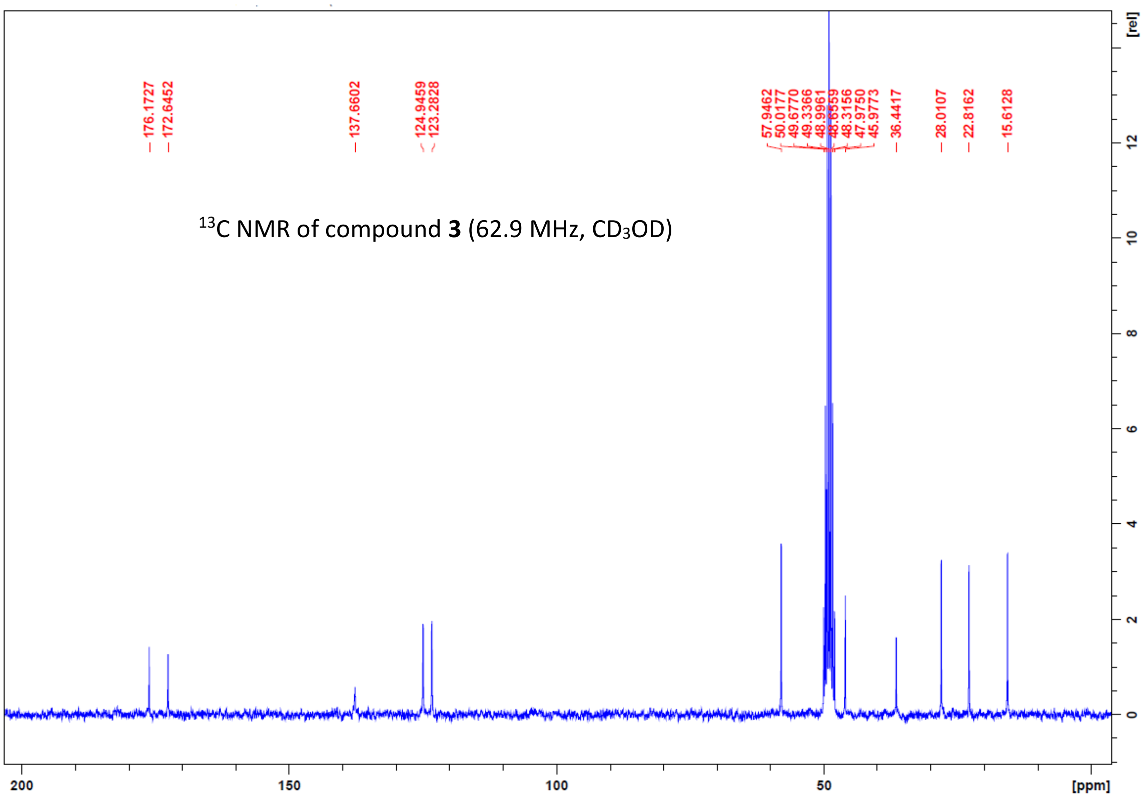
^{13}C NMR of compound **3** (62.9 MHz, CD_3OD)
176.1727 172.6452
137.6602
124.9459 123.2828
57.9462 50.0177 49.6770 49.3366 48.9961 48.8559 48.3156 47.9750 45.9773
36.4417 28.0107 22.8162 15.6128
[rel]
[ppm]

COSY spectra of compound **3** (250.13 MHz, CD_3OD)

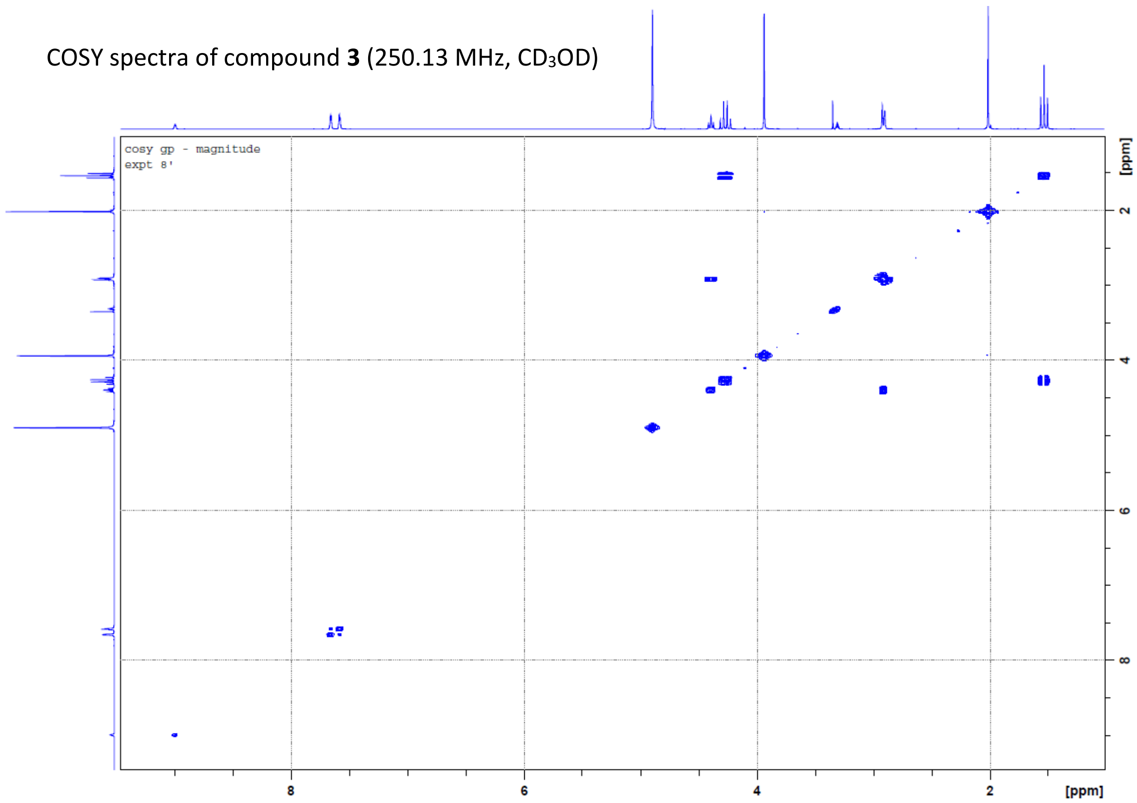

HSQC spectra of compound **3** (250.13 MHz, CD_3OD)

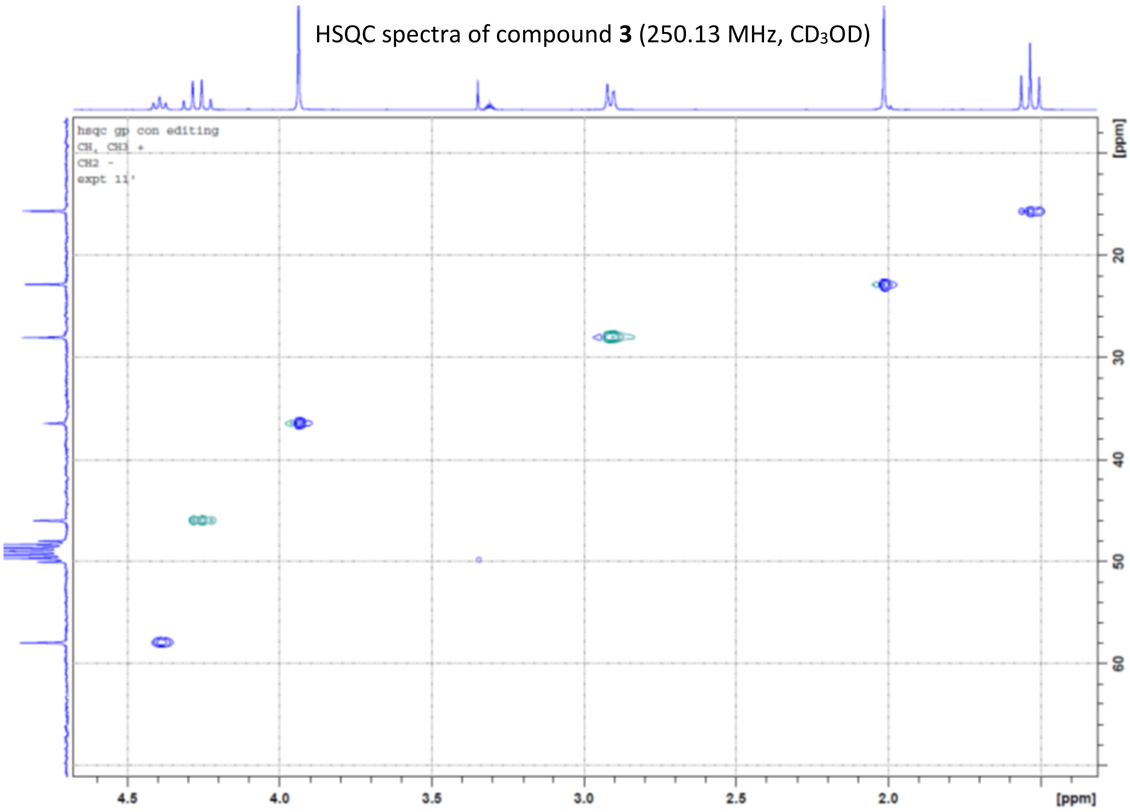

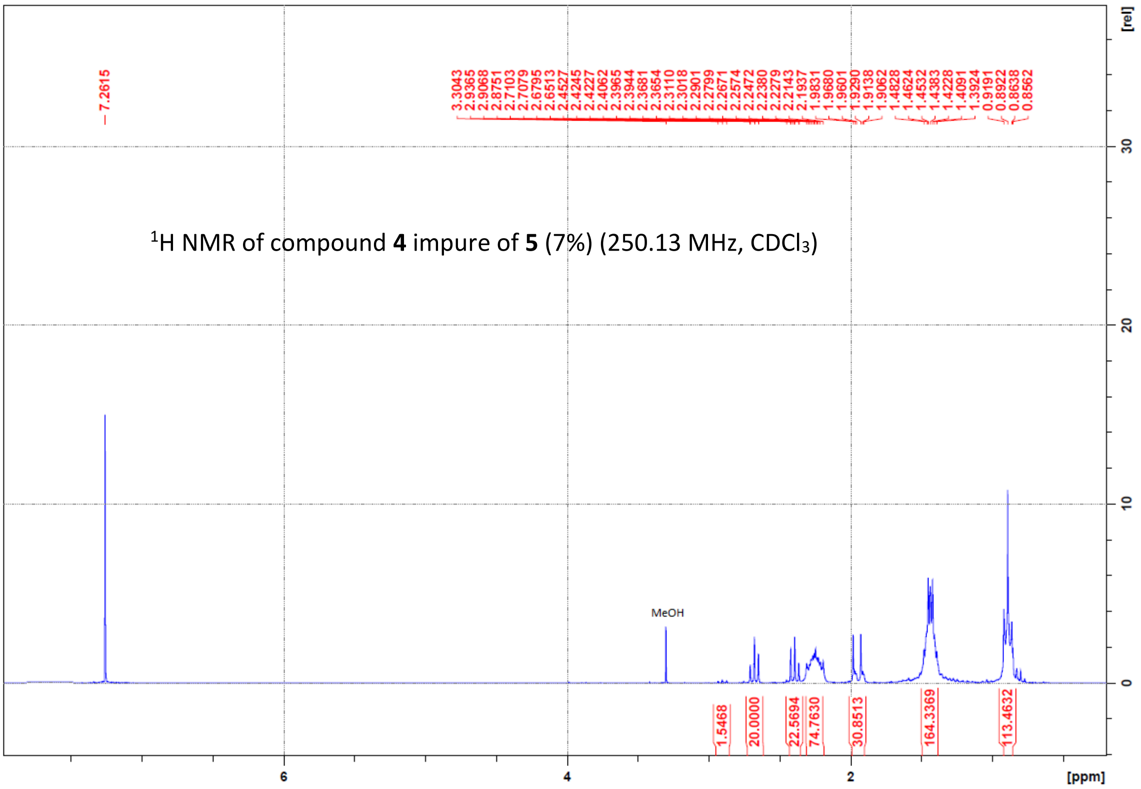
1H NMR of compound 4 impure of 5 (7%) (250.13 MHz, CDCl3)
MeOH

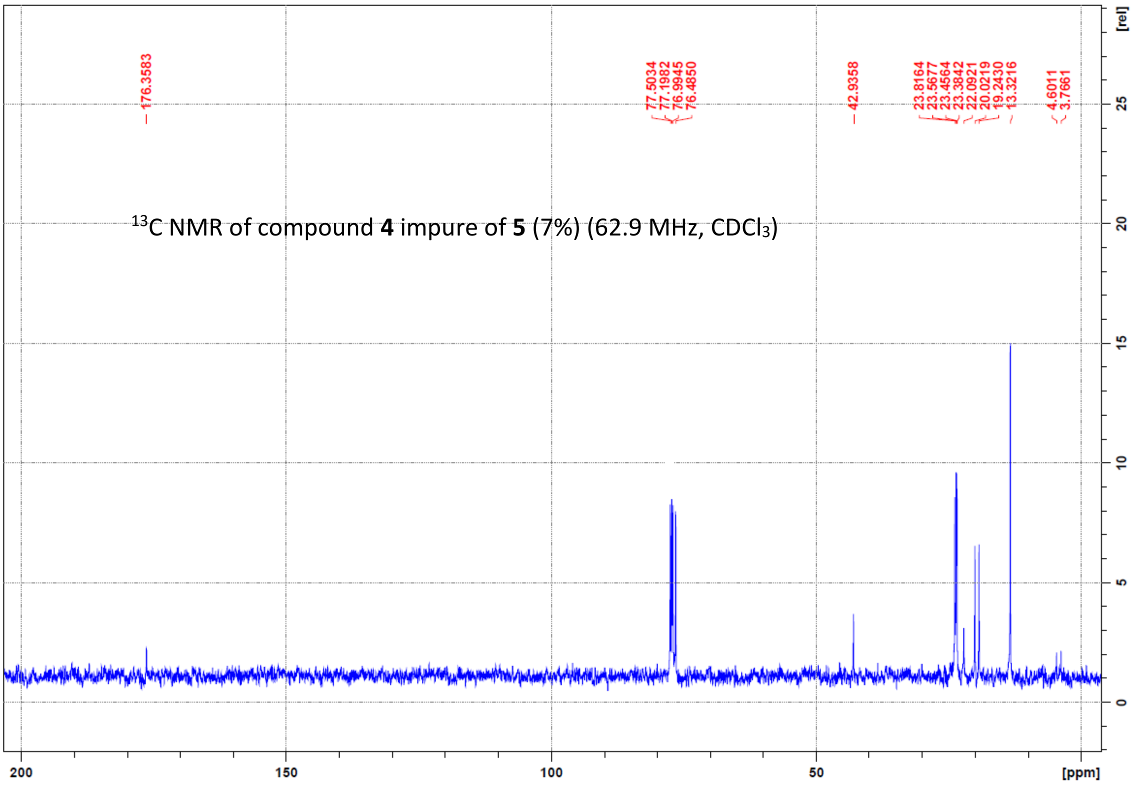
13C NMR of compound 4 impure of 5 (7%) (62.9 MHz, CDCl3)

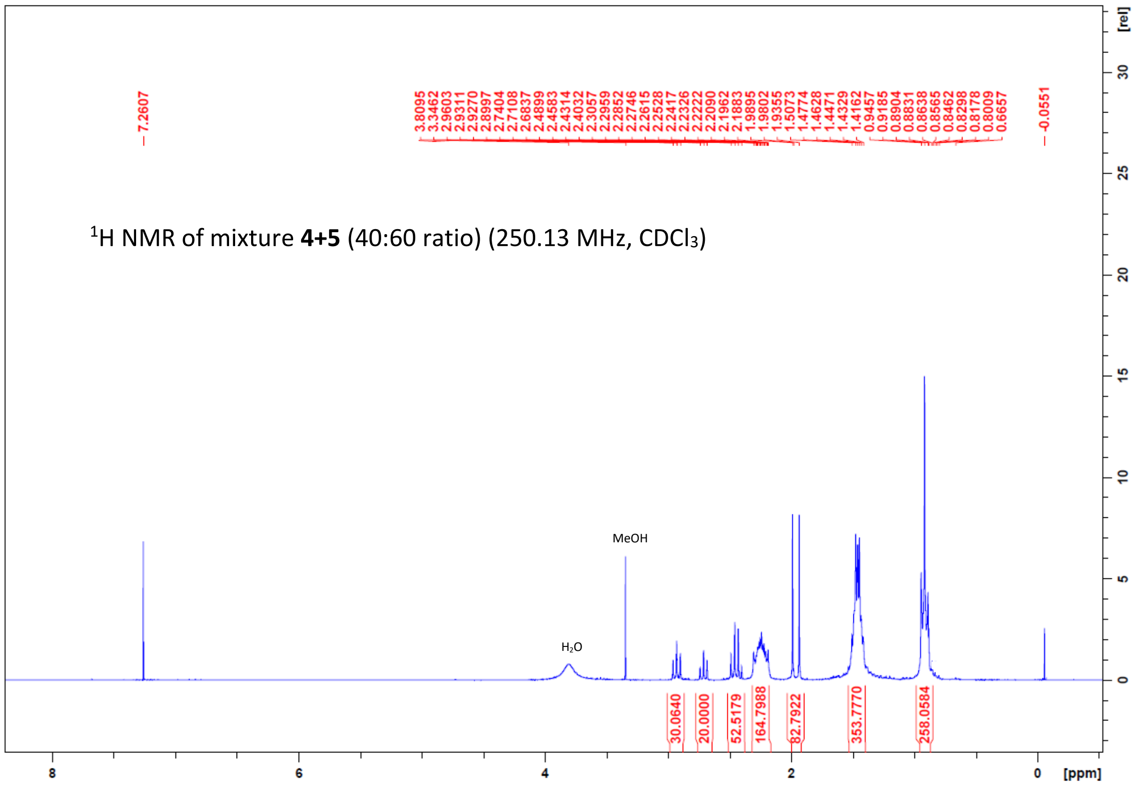

^{1}H NMR of mixture **4+5** (40:60 ratio) (250.13 MHz, CDCl3)
H2O
MeOH
[ppm]
[rel]

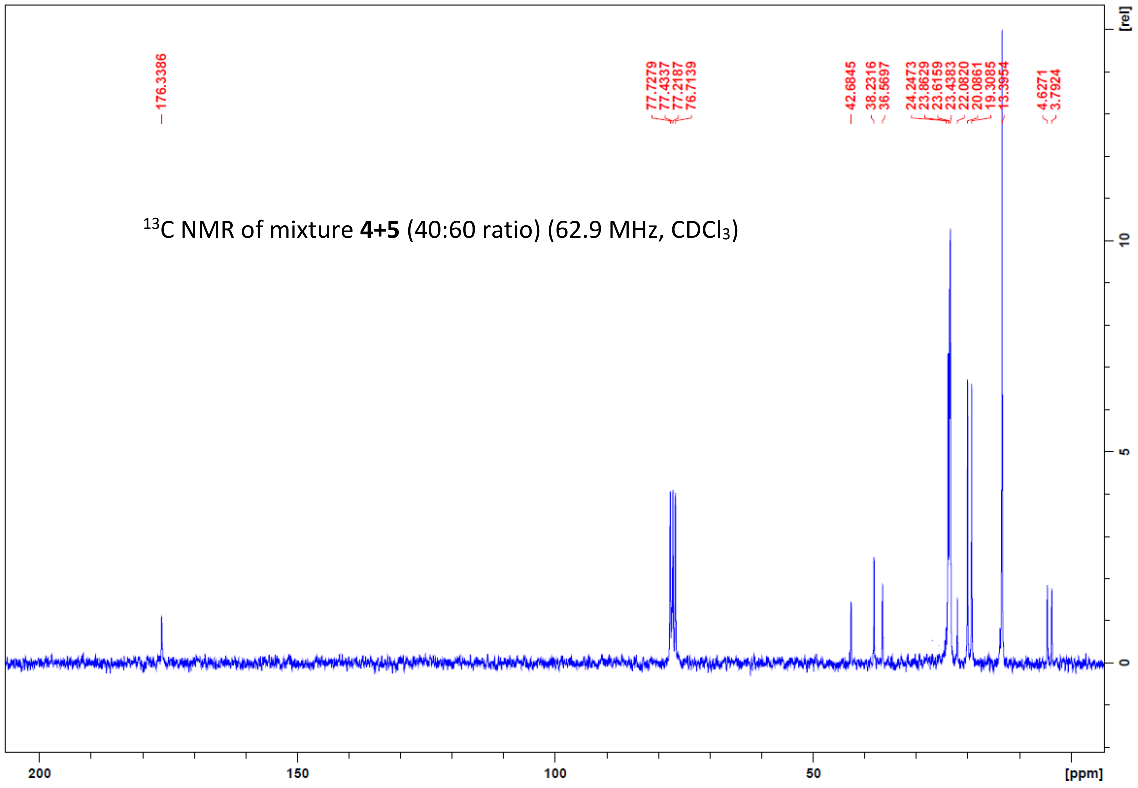

^{13}C NMR of mixture **4+5** (40:60 ratio) (62.9 MHz, CDCl3)
[ppm]
[rel]

COSY spectra of mixture **4+5** (40:60 ratio) (250.13 MHz, $CDCl_3$)

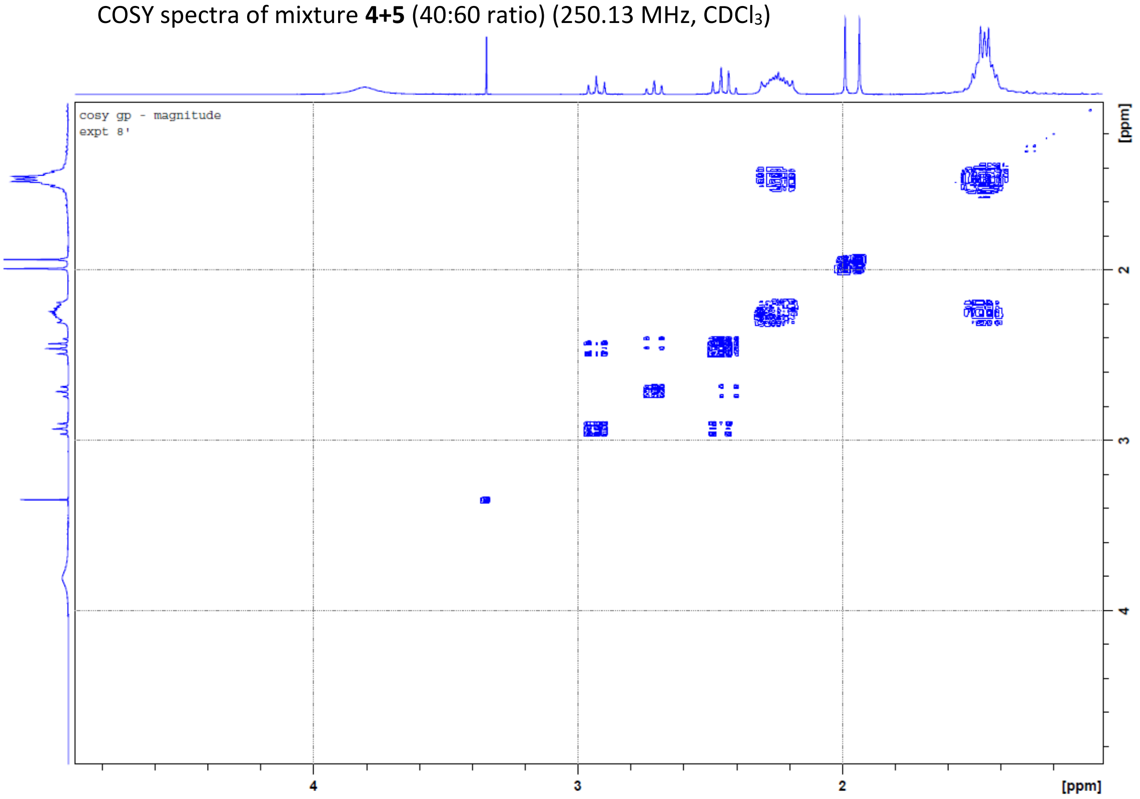

HSQC spectra of mixture **4+5** (40:60 ratio) (250.13 MHz, $CDCl_3$)

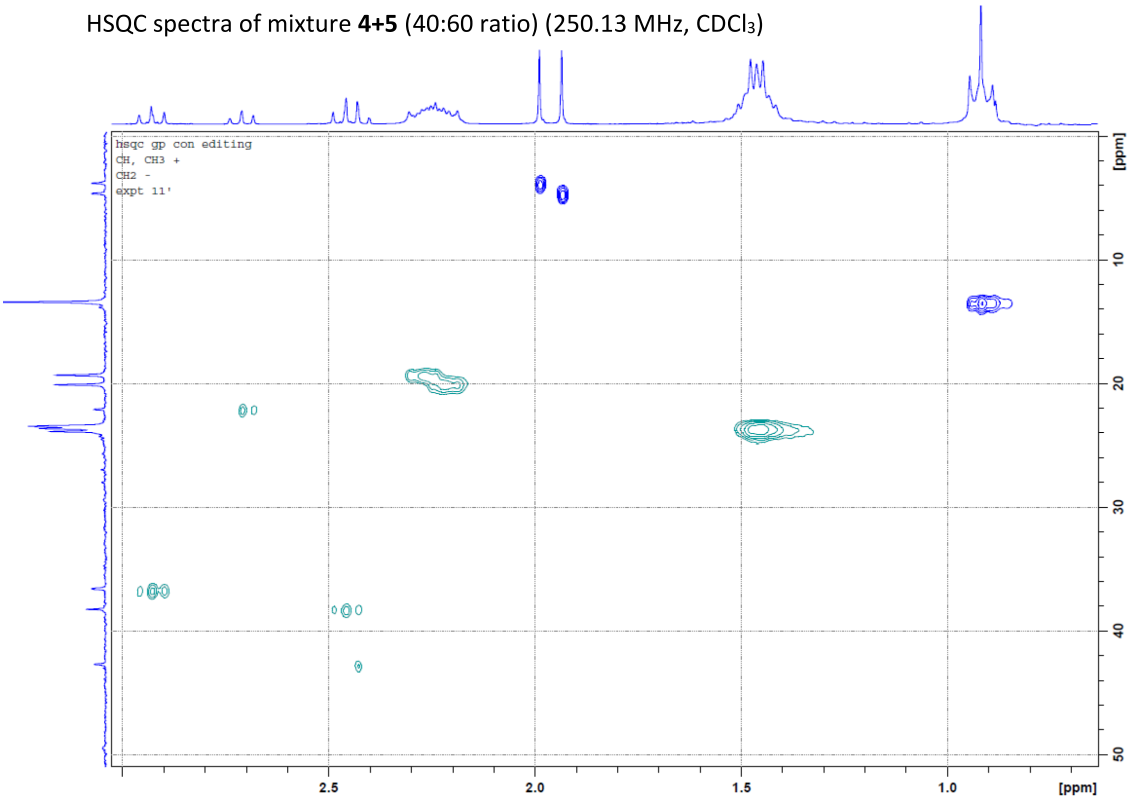

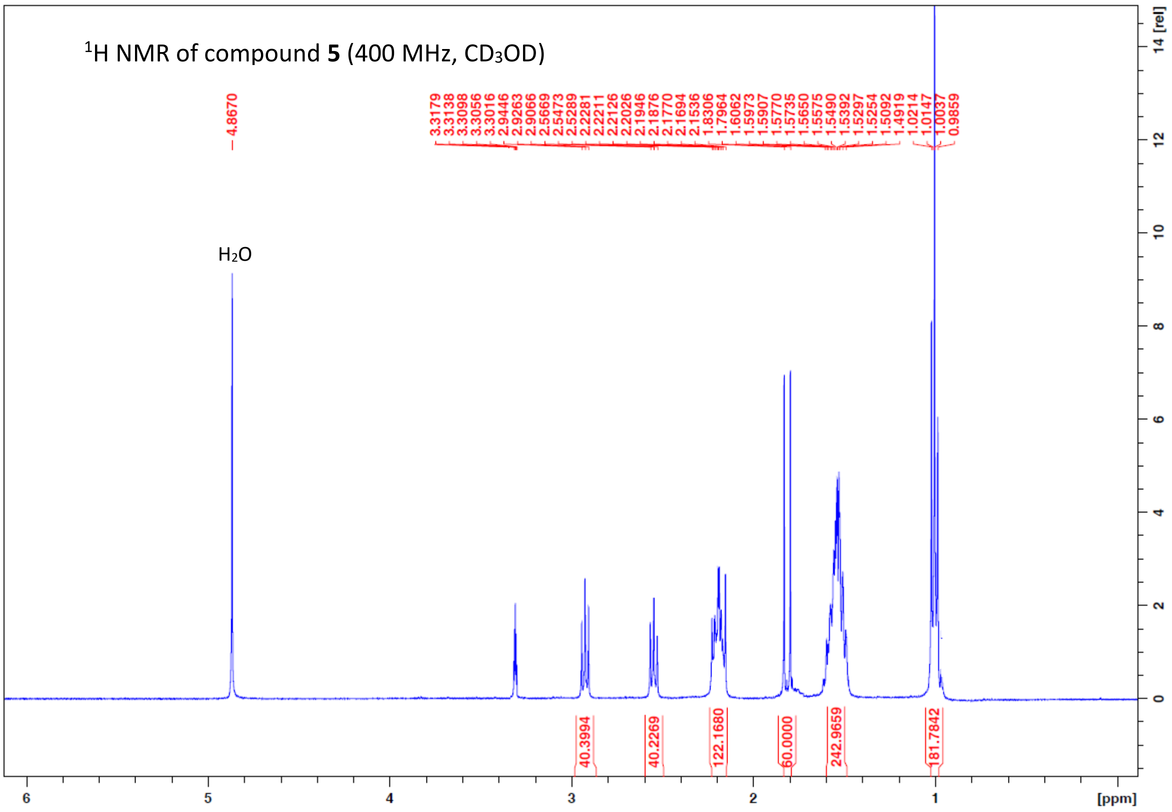
^{1}H NMR of compound **5** (400 MHz, CD_3OD)
H_2O
[ppm]
[rel]

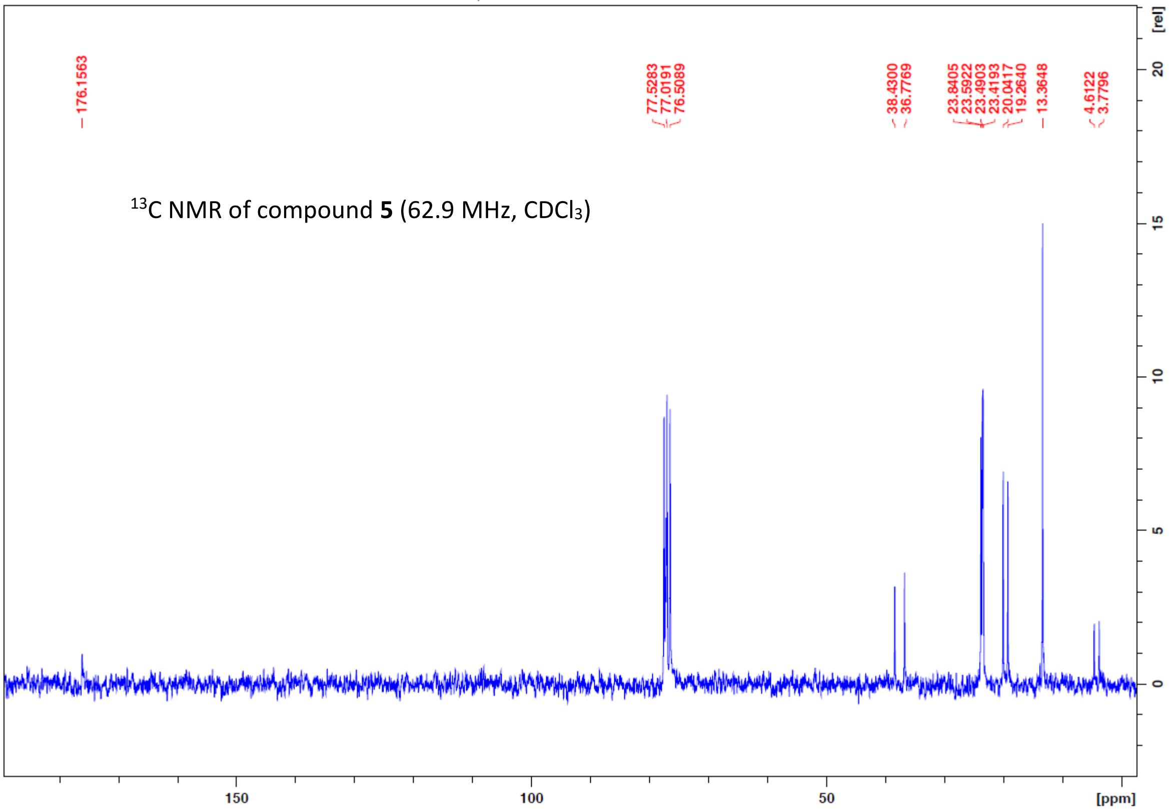
^{13}C NMR of compound **5** (62.9 MHz, $CDCl_3$)
[ppm]
[rel]

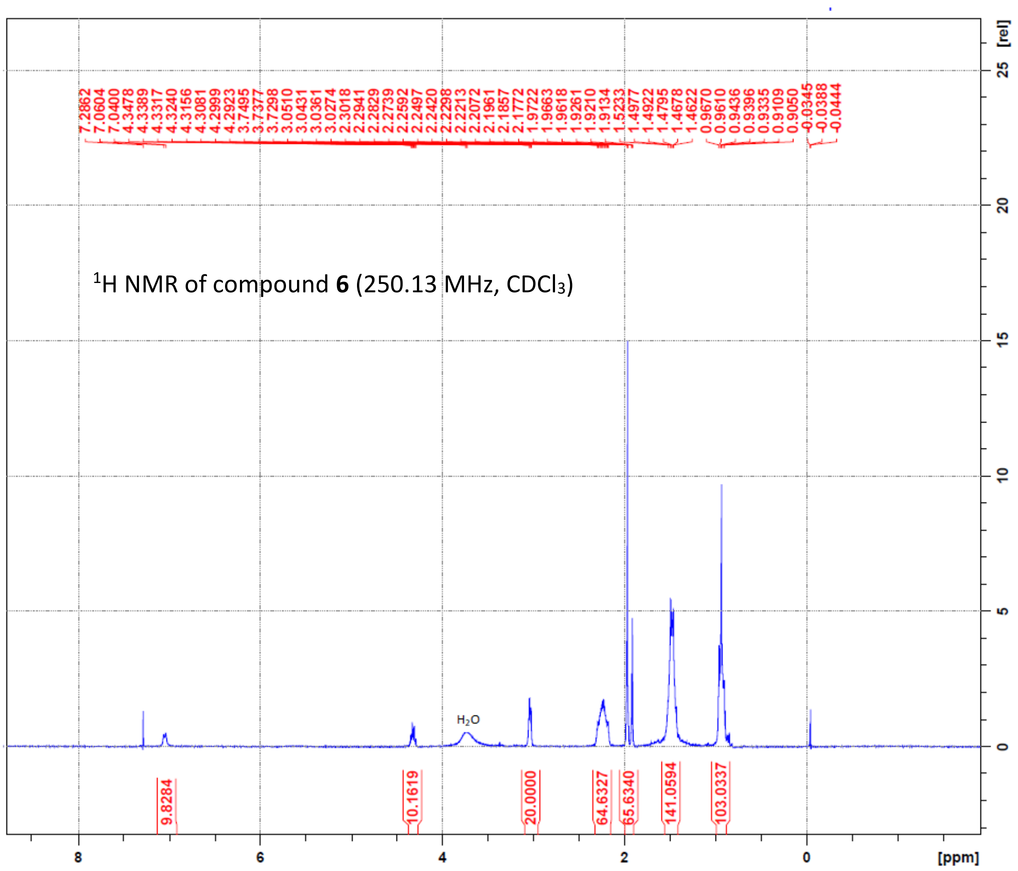
¹H NMR of compound 6 (250.13 MHz, CDCl₃)
H₂O
[rel]
[ppm]

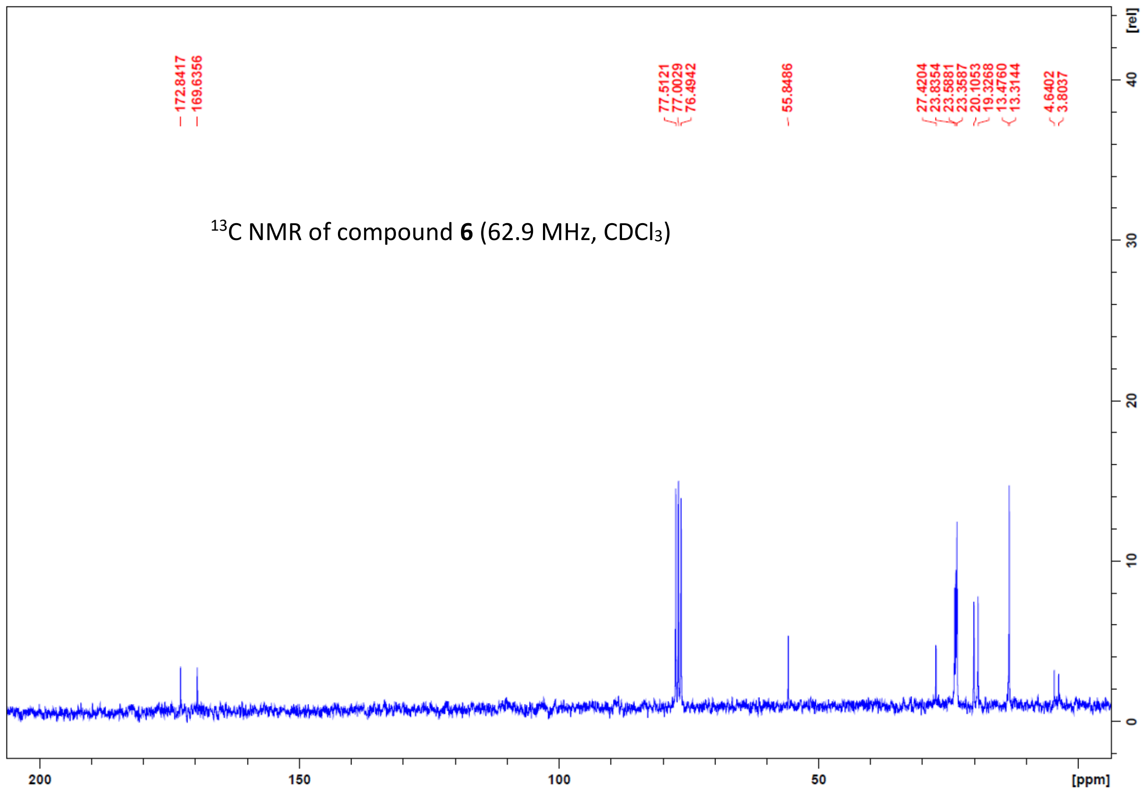
¹³C NMR of compound 6 (62.9 MHz, CDCl₃)
172.8417
169.6356
77.5121
77.0029
76.4942
55.8486
27.4204
23.8354
23.5881
23.3587
20.1053
19.3268
13.4760
13.3144
4.6402
3.8037
[rel]
[ppm]

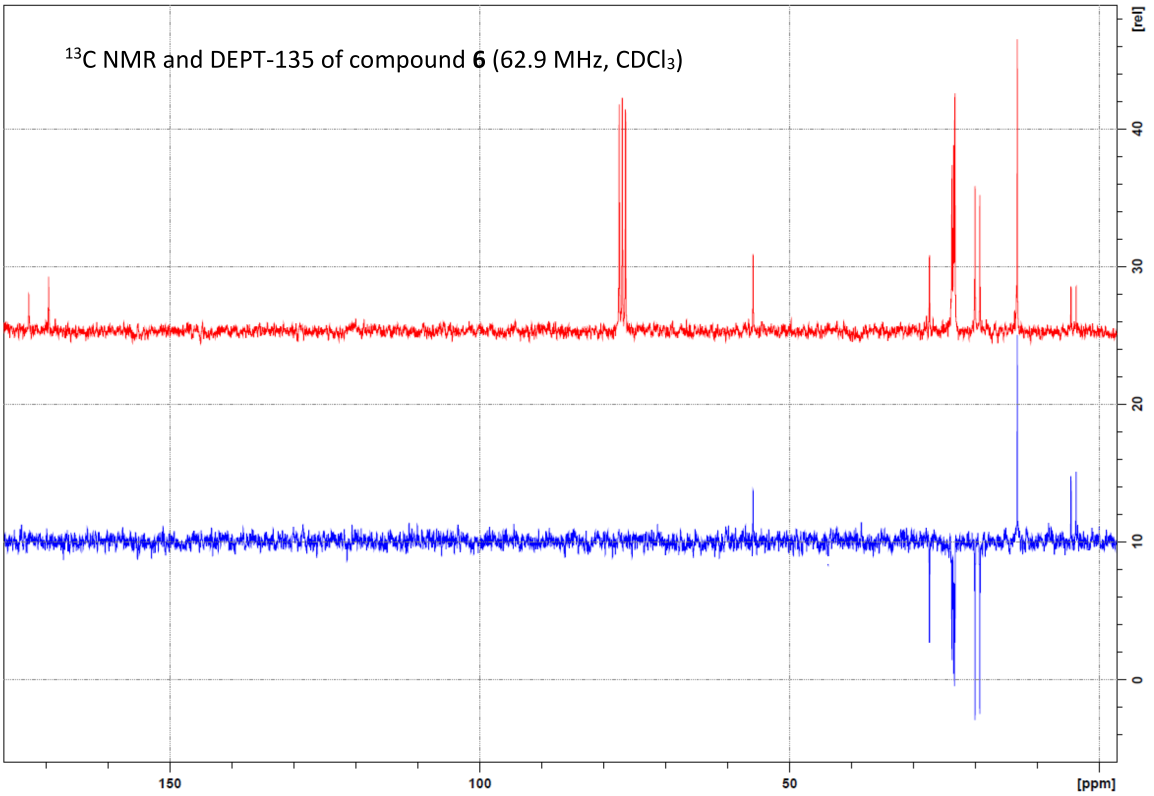
[rel]
40
30
20
10
0
150
100
50
[ppm]

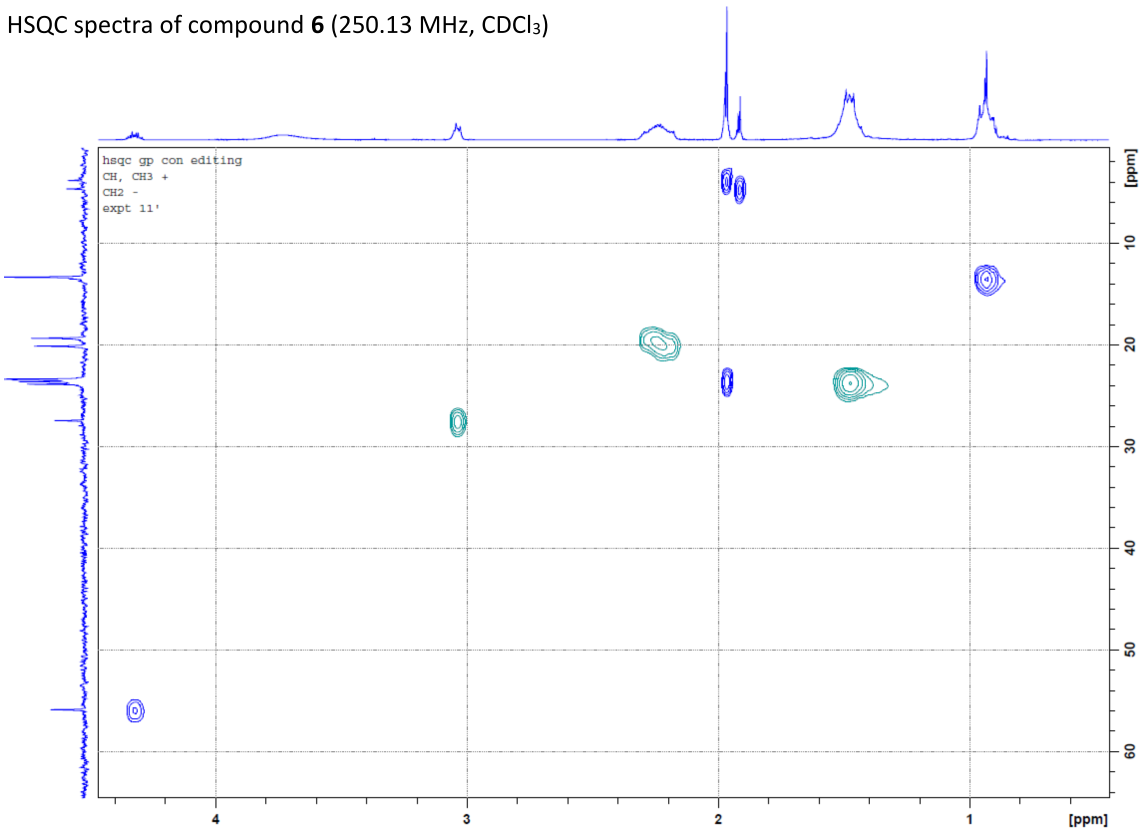
hsqc gp con editing
CH, CH3 +
CH2 -
expt 11'
[ppm]
10
20
30
40
50
60
4
3
2
1
[ppm]

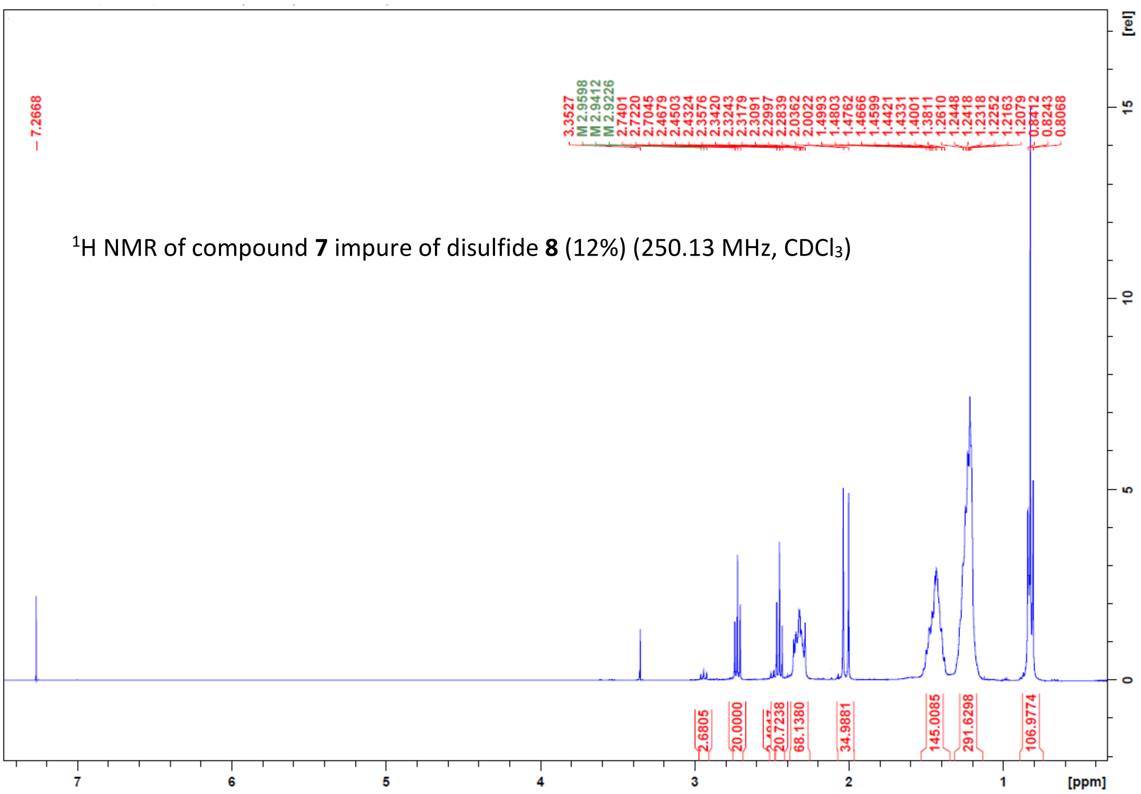
^{1}H NMR of compound **7** impure of disulfide **8** (12%) (250.13 MHz, $CDCl_3$)
[ppm]
[rel]

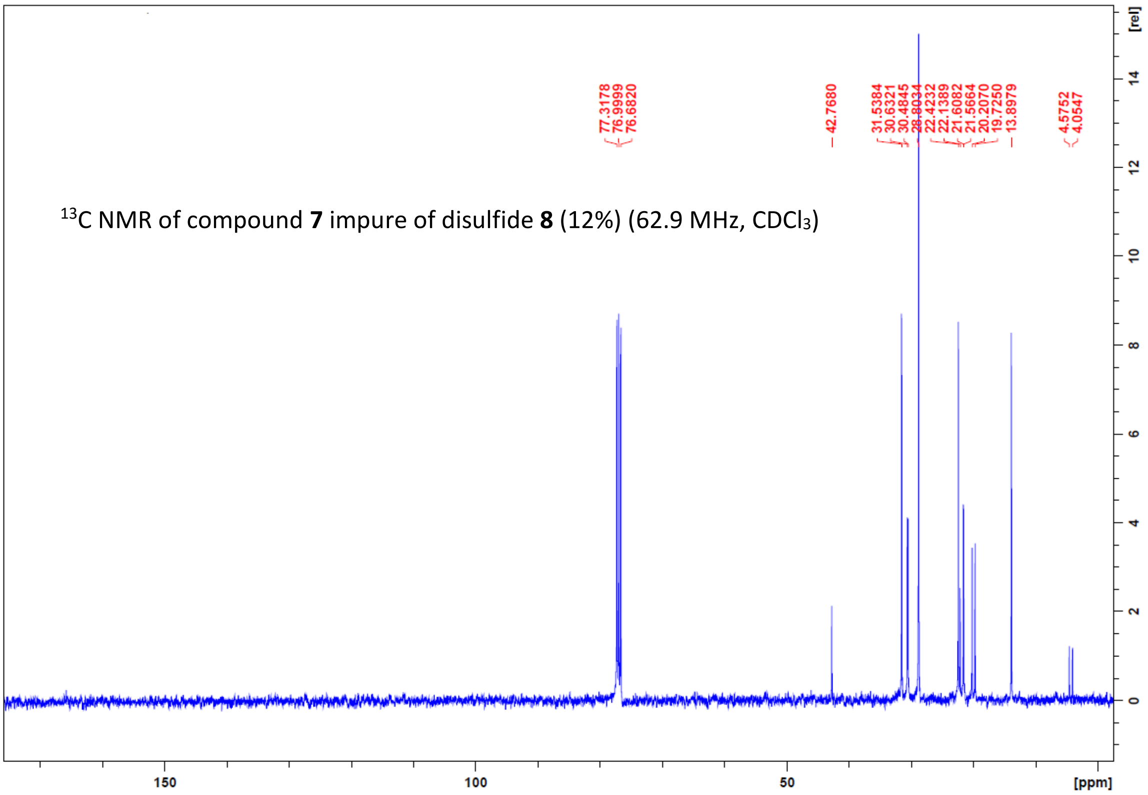
^{13}C NMR of compound **7** impure of disulfide **8** (12%) (62.9 MHz, $CDCl_3$)
[ppm]
[rel]

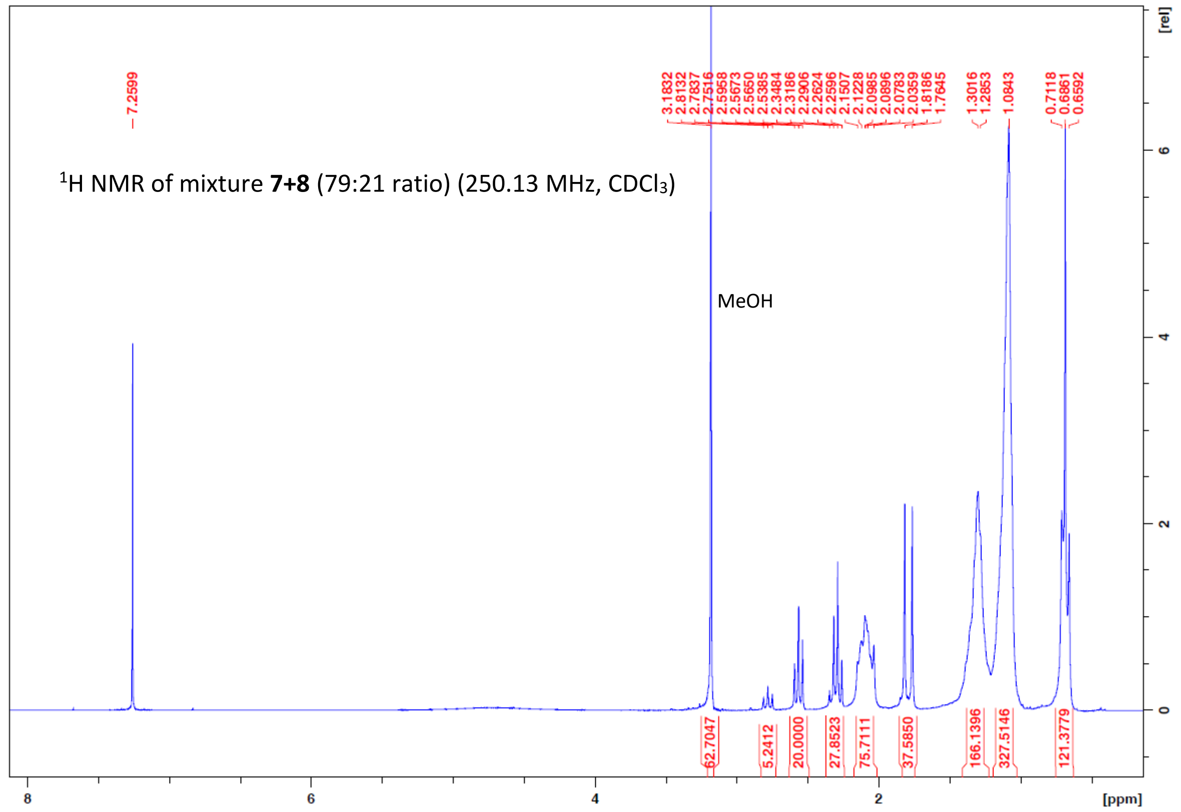

1H NMR of mixture 7+8 (79:21 ratio) (250.13 MHz, CDCl3)
MeOH

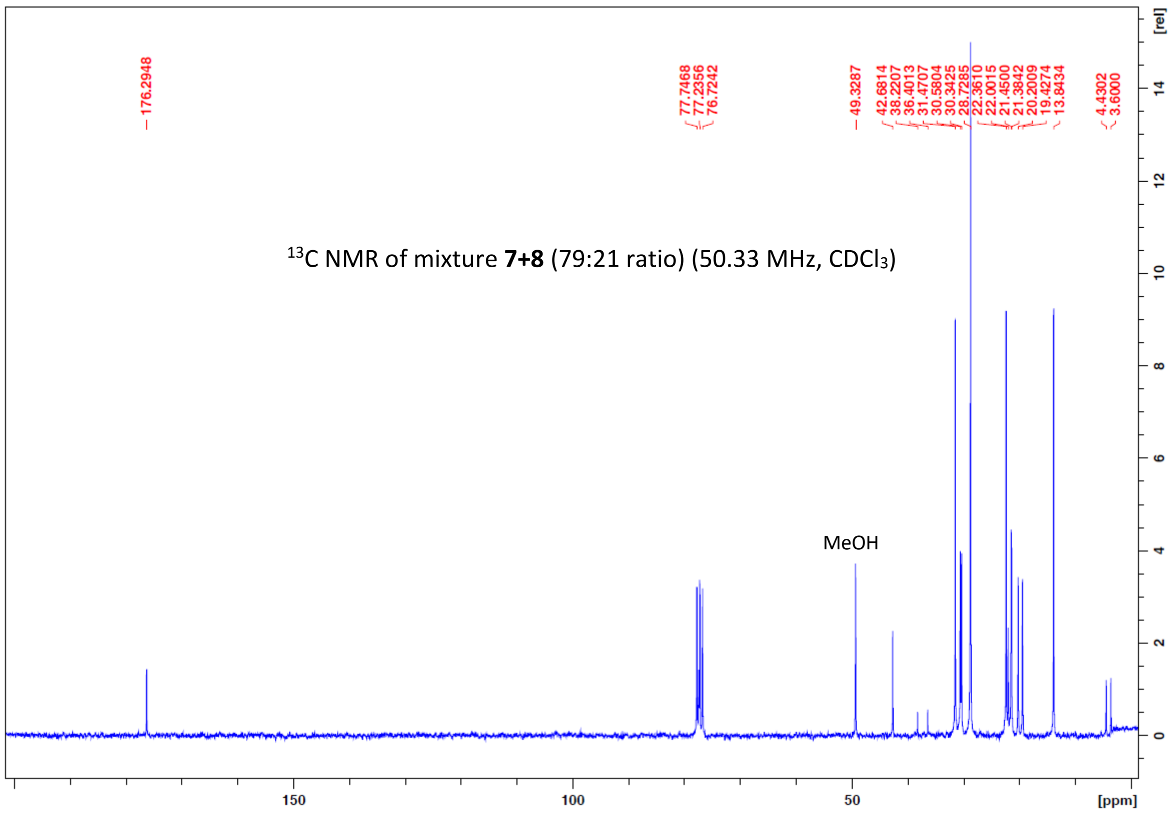

13C NMR of mixture 7+8 (79:21 ratio) (50.33 MHz, CDCl3)
MeOH

COSY spectra of mixture **7+8** (79:21 ratio) (250.13 MHz, $CDCl_3$)

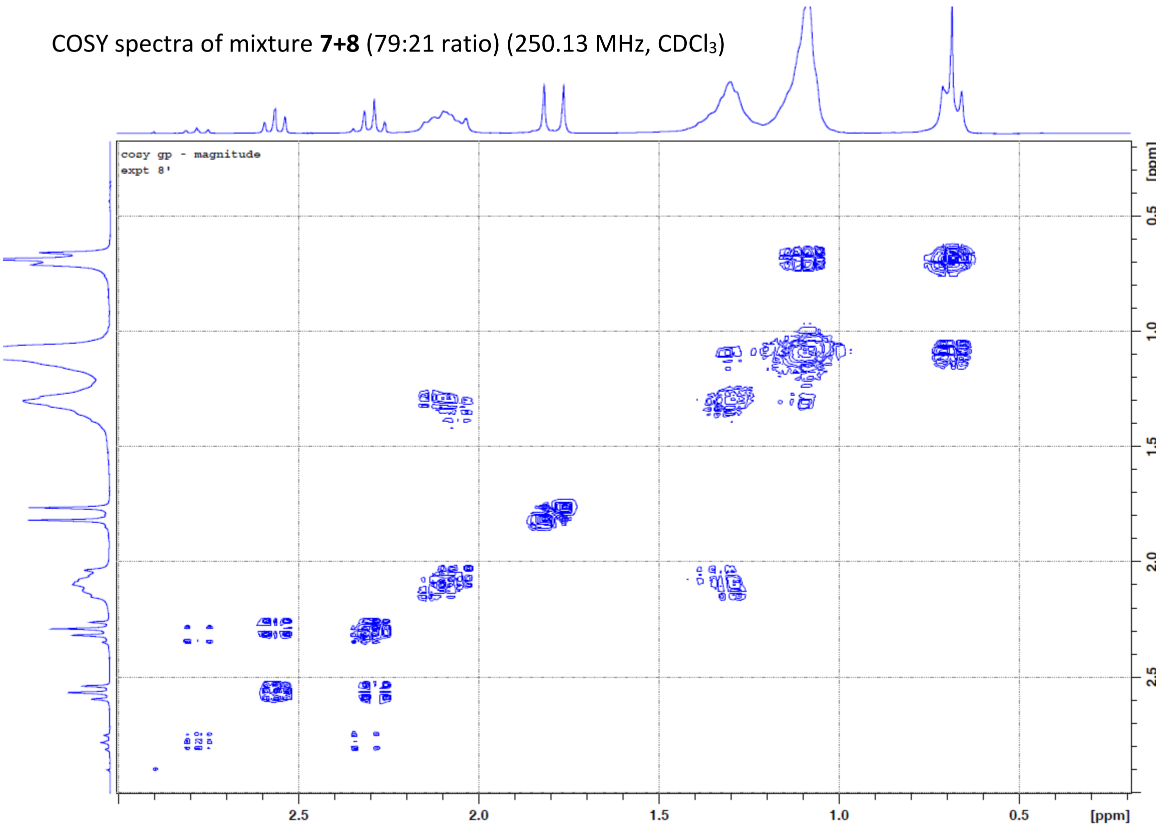

HSQC spectra of mixture **7+8** (79:21 ratio) (250.13 MHz,

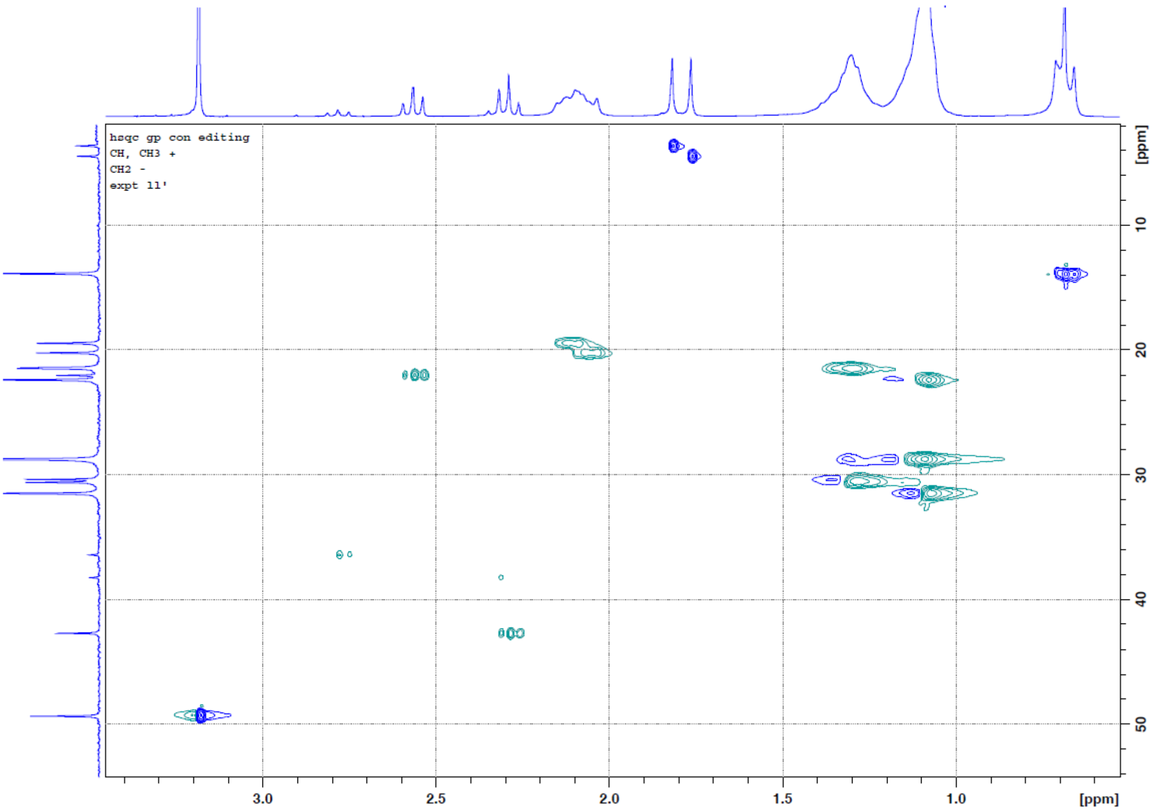

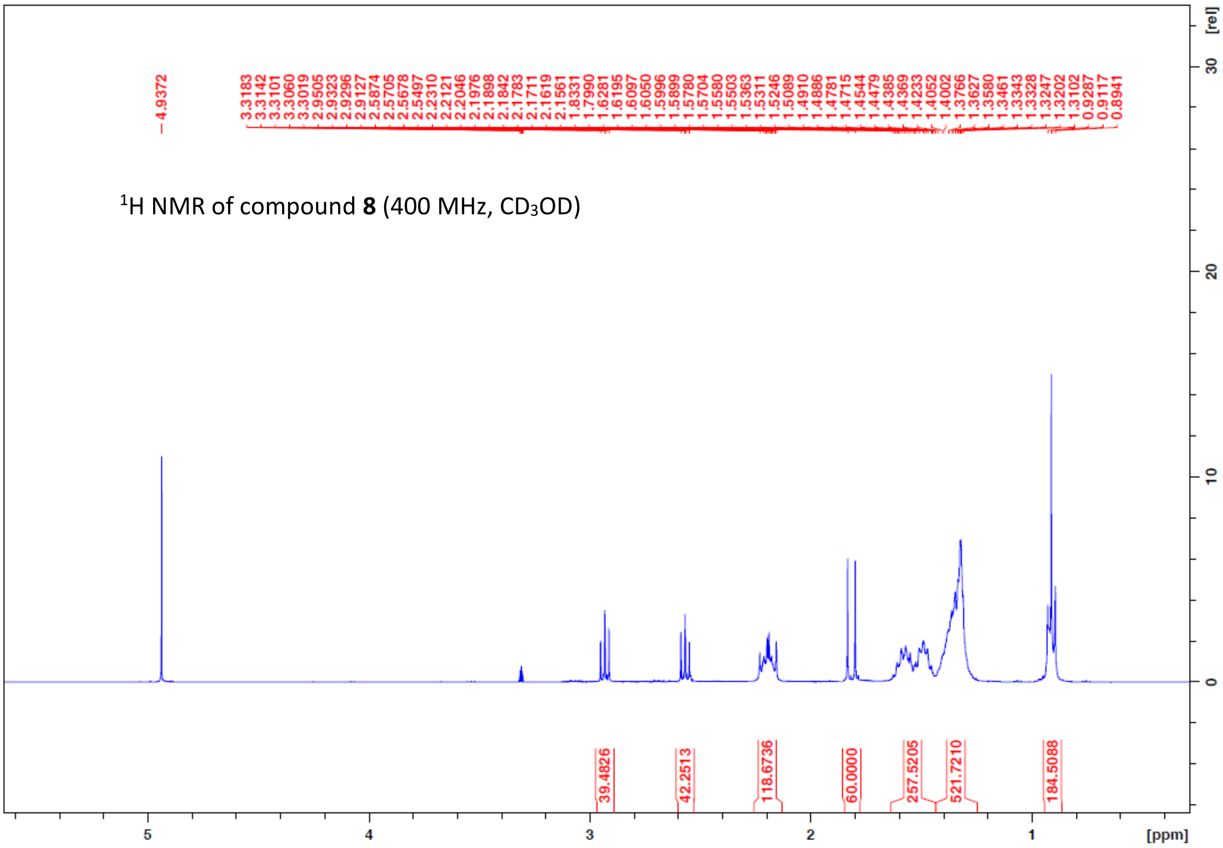

1H NMR of compound 8 (400 MHz, CD3OD)

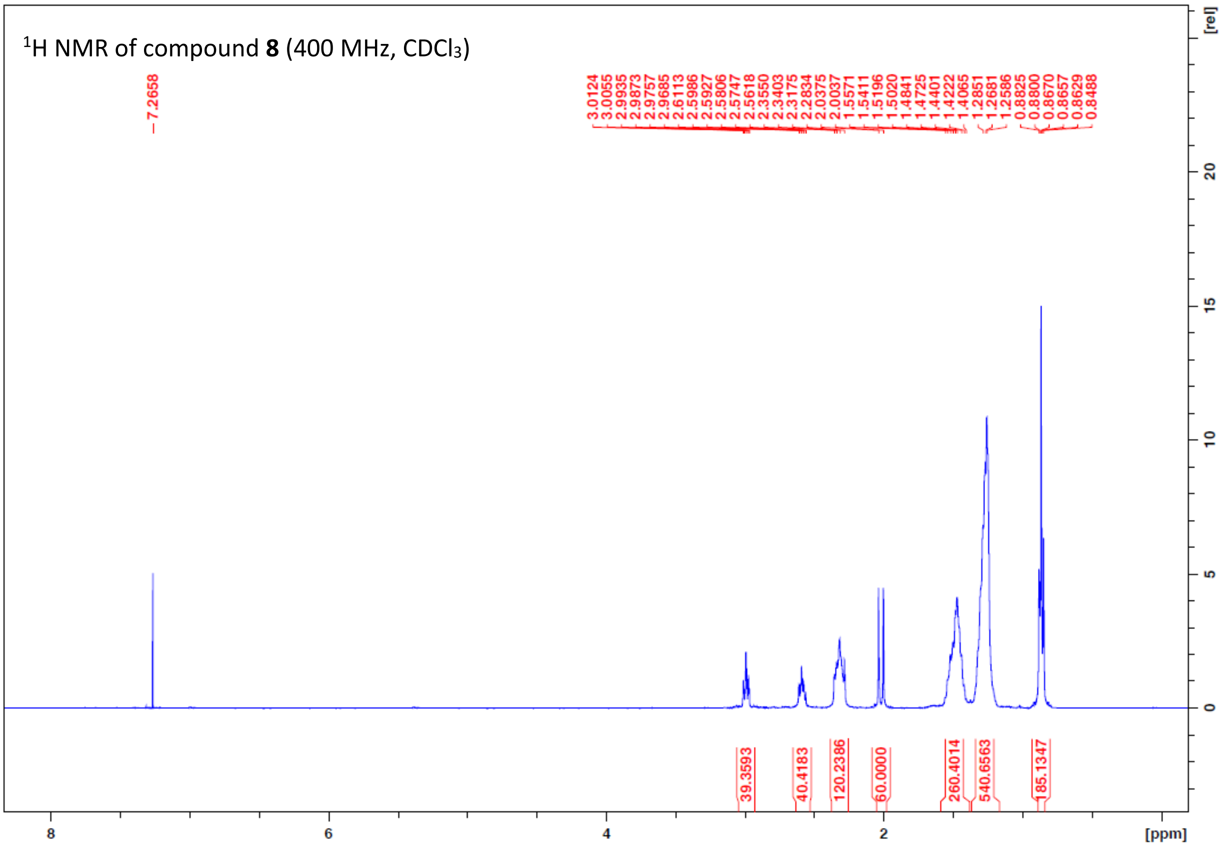

1H NMR of compound 8 (400 MHz, CDCl3)

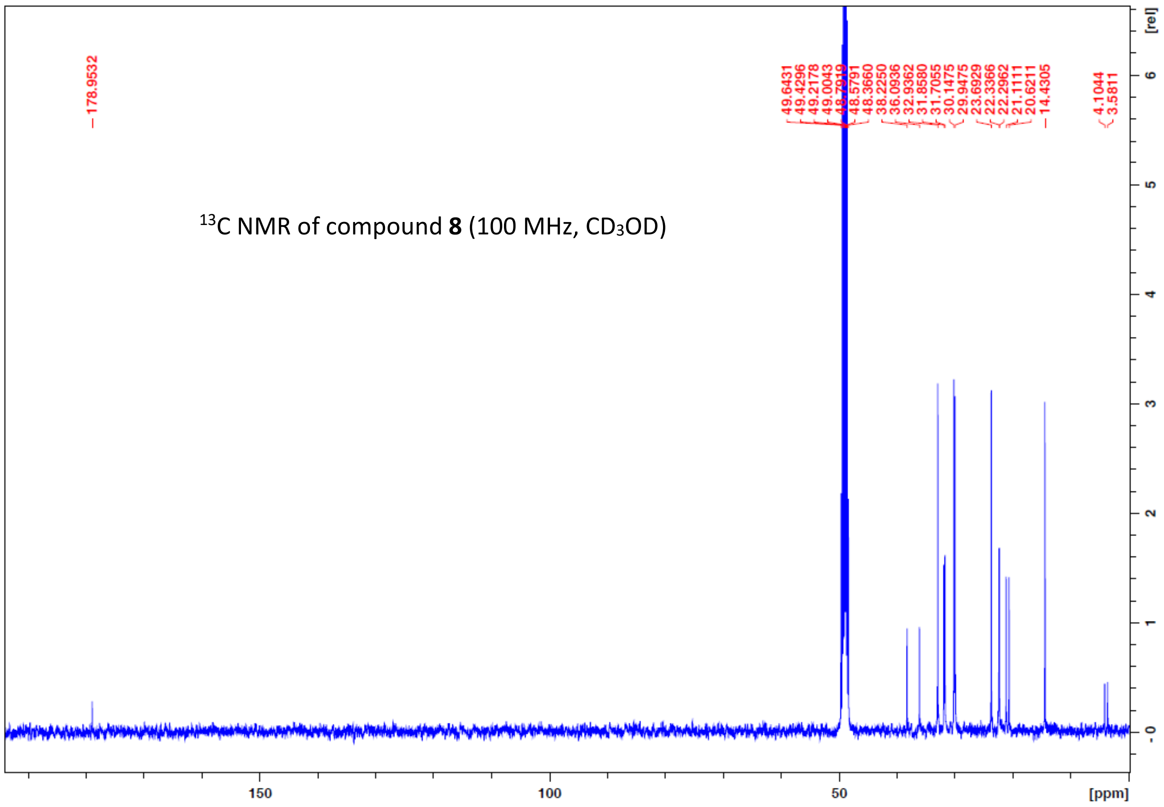

^{13}C NMR of compound **8** (100 MHz, CD$_3$OD)
178.9532
49.6431
49.4296
49.2178
49.0043
48.7919
48.5791
48.3660
38.2250
36.0936
32.9362
31.8580
31.7055
30.1475
29.9475
23.6929
22.3366
22.2962
21.1111
20.6211
14.4305
4.1044
3.5811
[rel]
150
100
50
[ppm]

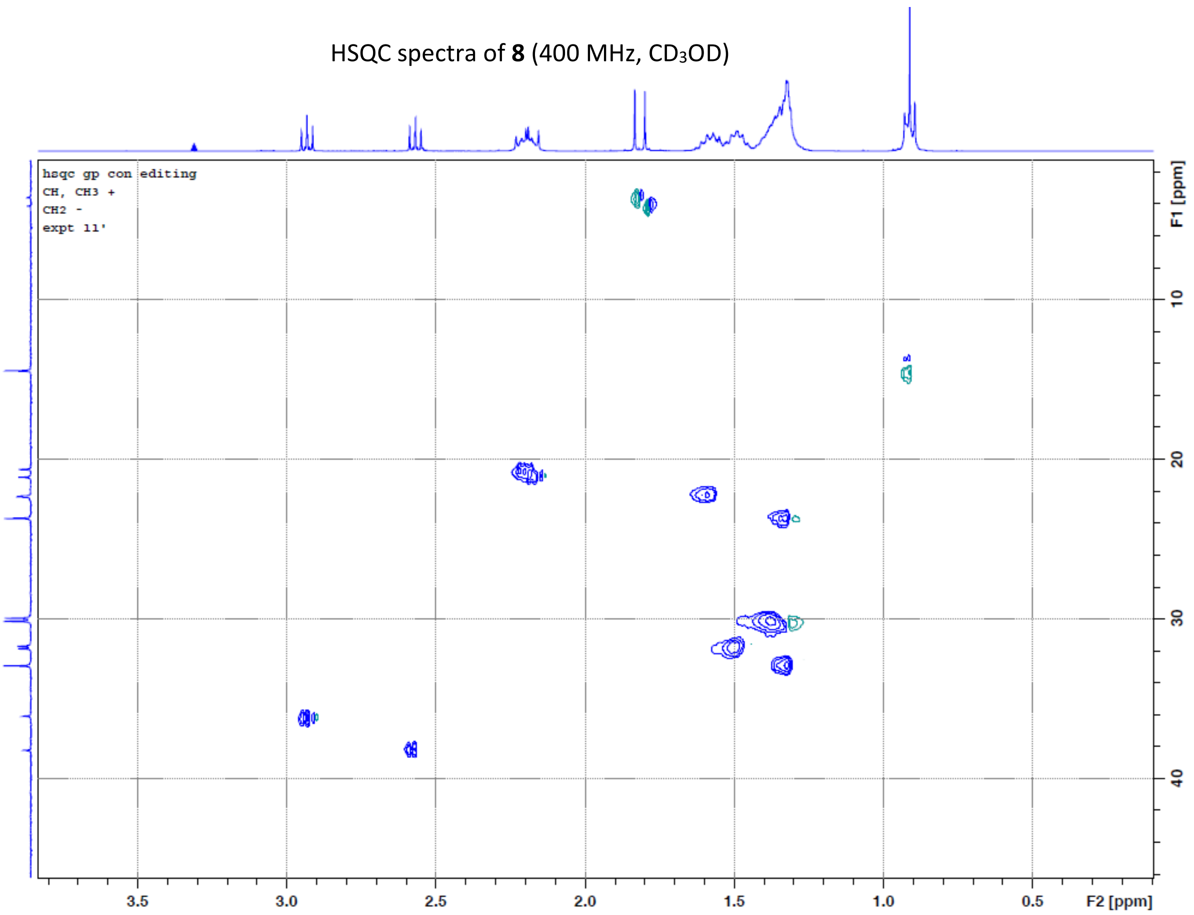

HSQC spectra of **8** (400 MHz, CD$_3$OD)
hsqc gp con editing
CH, CH3 +
CH2 -
expt 11'
F1 [ppm]
10
20
30
40
3.5
3.0
2.5
2.0
1.5
1.0
0.5
F2 [ppm]

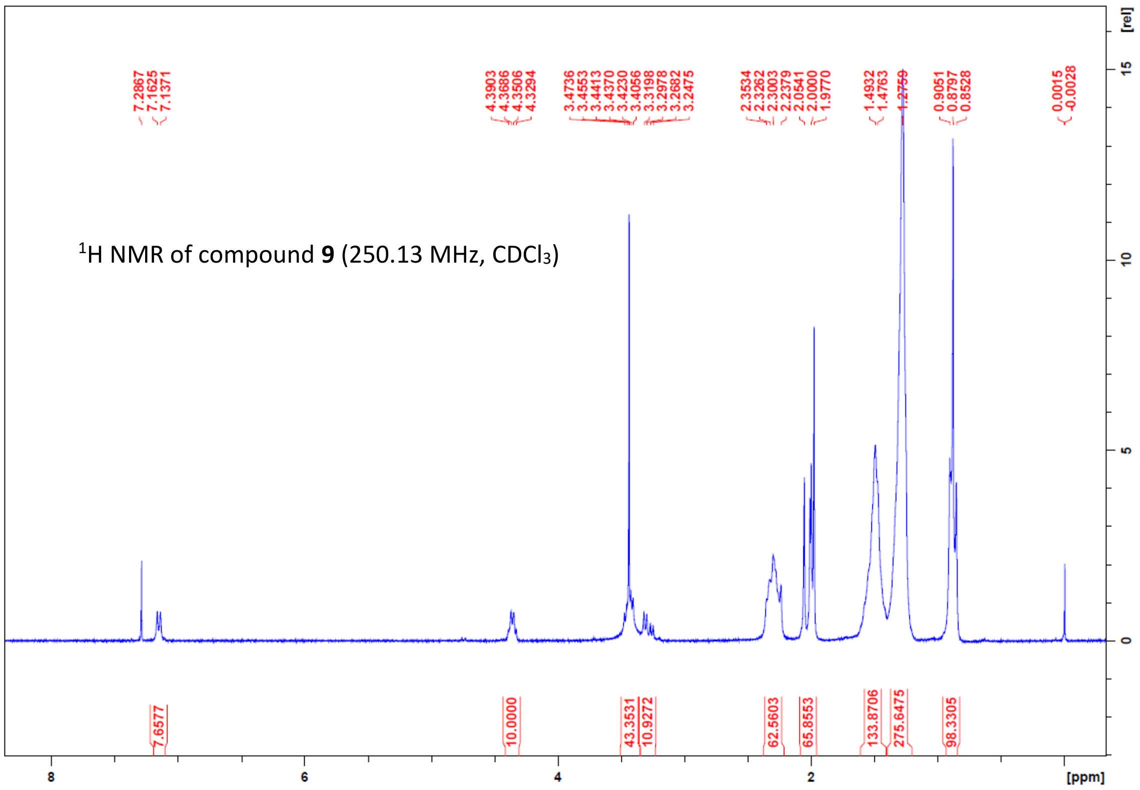
1H NMR of compound 9 (250.13 MHz, CDCl3)
[ppm]
[rel]

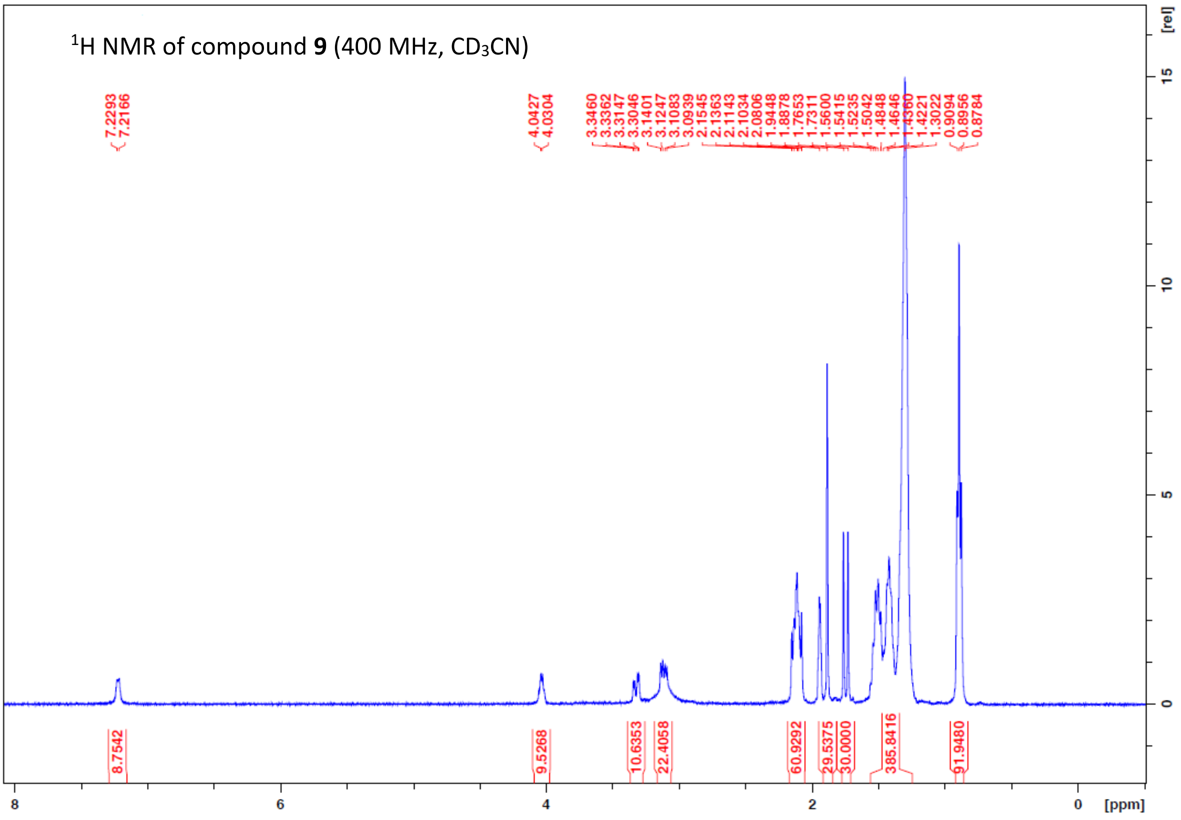
1H NMR of compound 9 (400 MHz, CD3CN)
[ppm]
[rel]

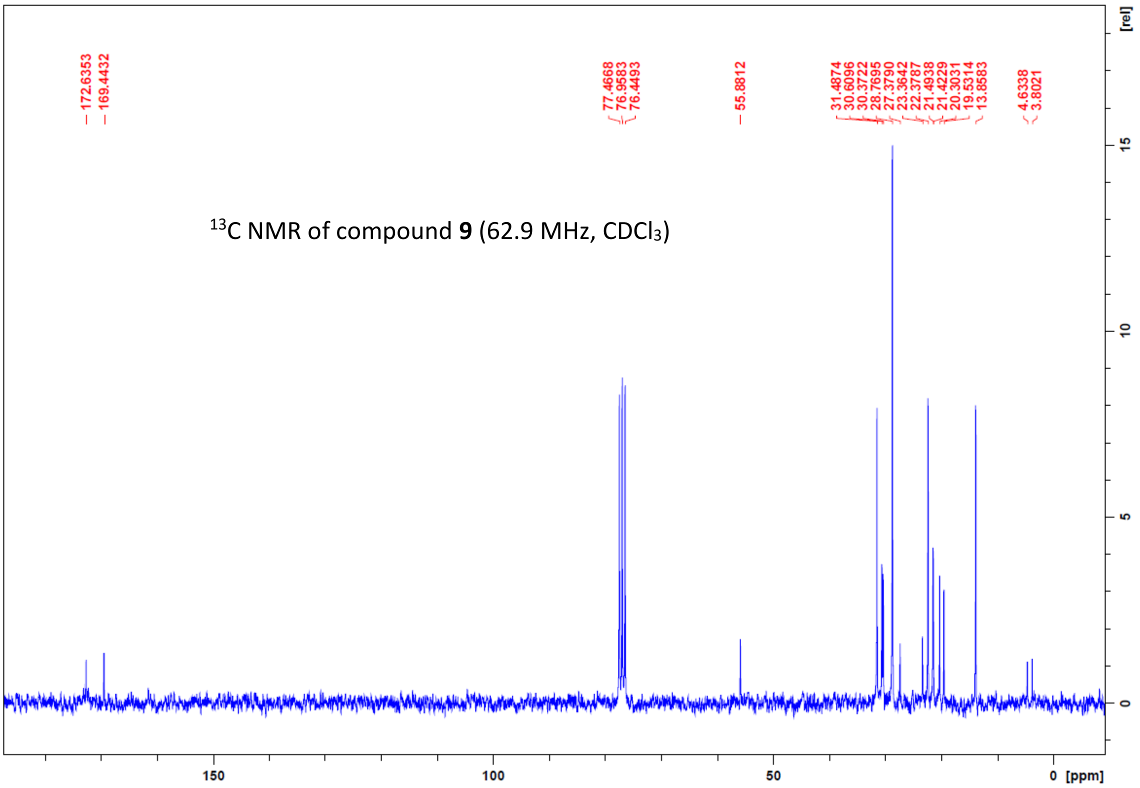

^{13}C NMR of compound **9** (62.9 MHz, $CDCl_3$)

^{1}H NMR of compound **10** (400 MHz, CD_3OD)

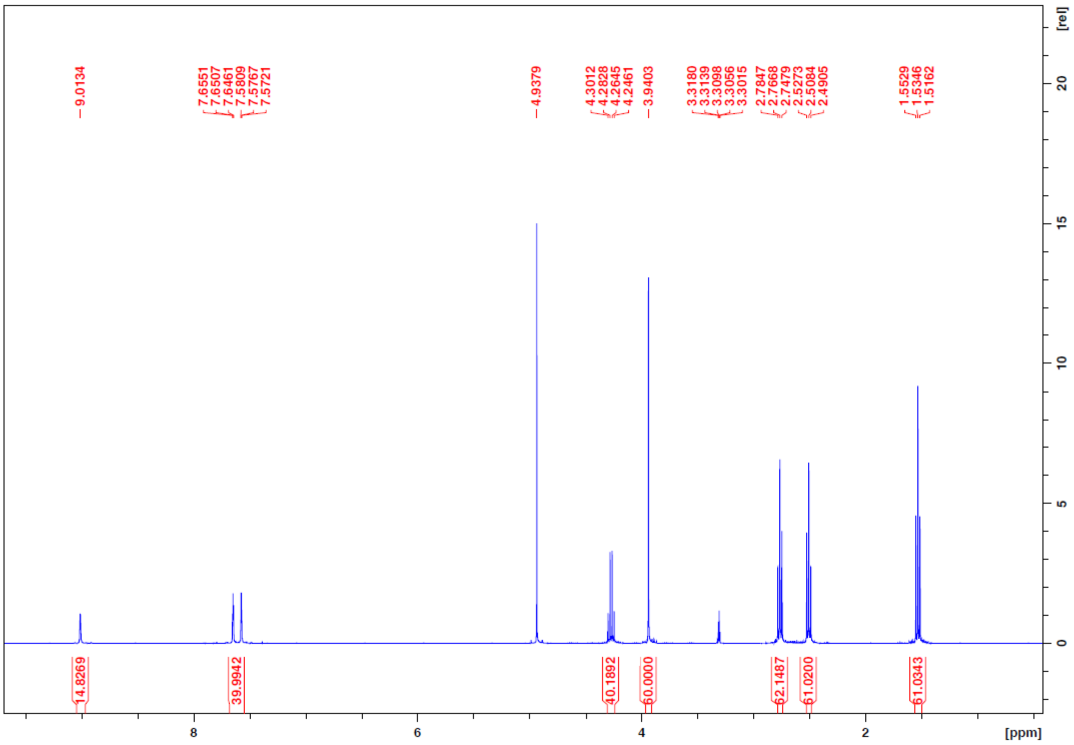

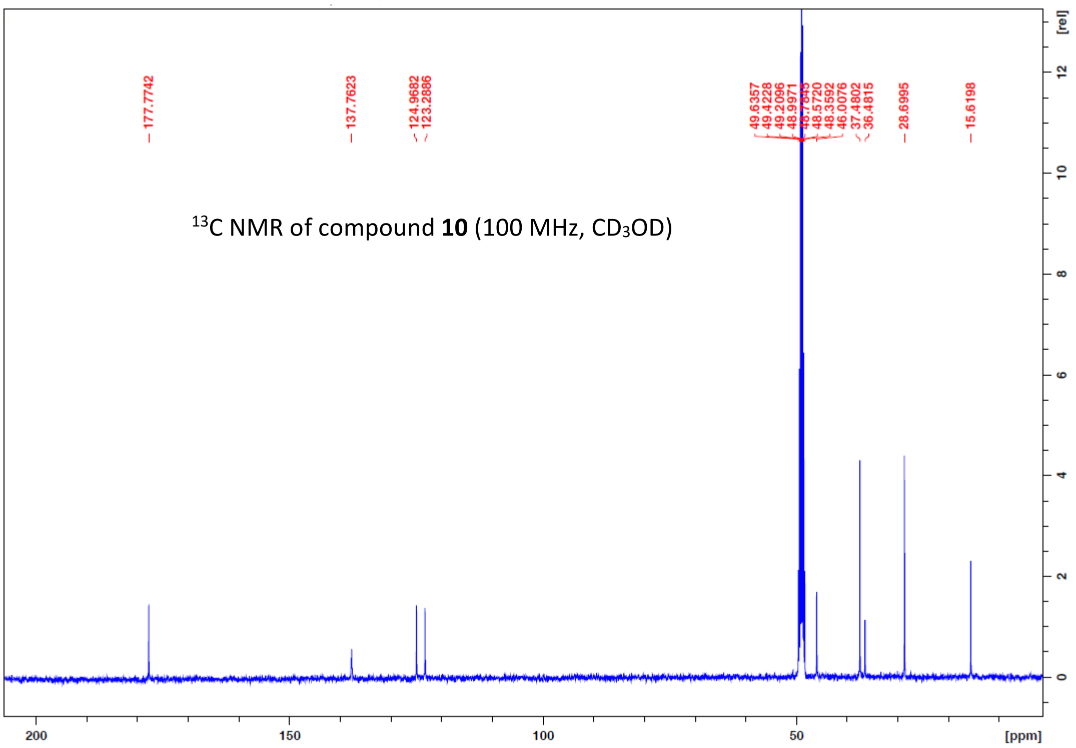

13C NMR of compound 10 (100 MHz, CD3OD)
177.7742
137.7623
124.9682
123.2886
49.6357
49.4228
49.2096
48.9971
48.7845
48.5720
48.3592
46.0076
37.4802
36.4815
28.6995
15.6198
[rel]
[ppm]

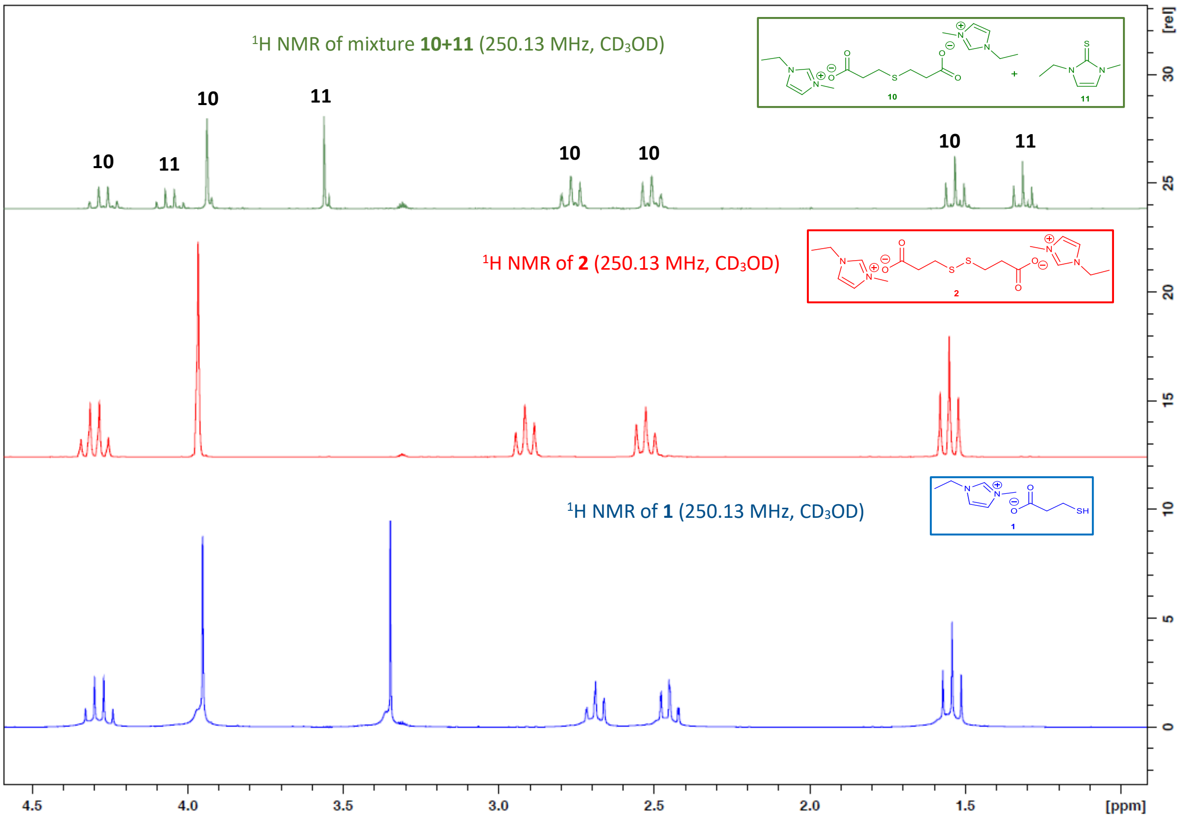

1H NMR of mixture 10+11 (250.13 MHz, CD3OD)
1H NMR of 2 (250.13 MHz, CD3OD)
1H NMR of 1 (250.13 MHz, CD3OD)
10
11
[rel]
[ppm]

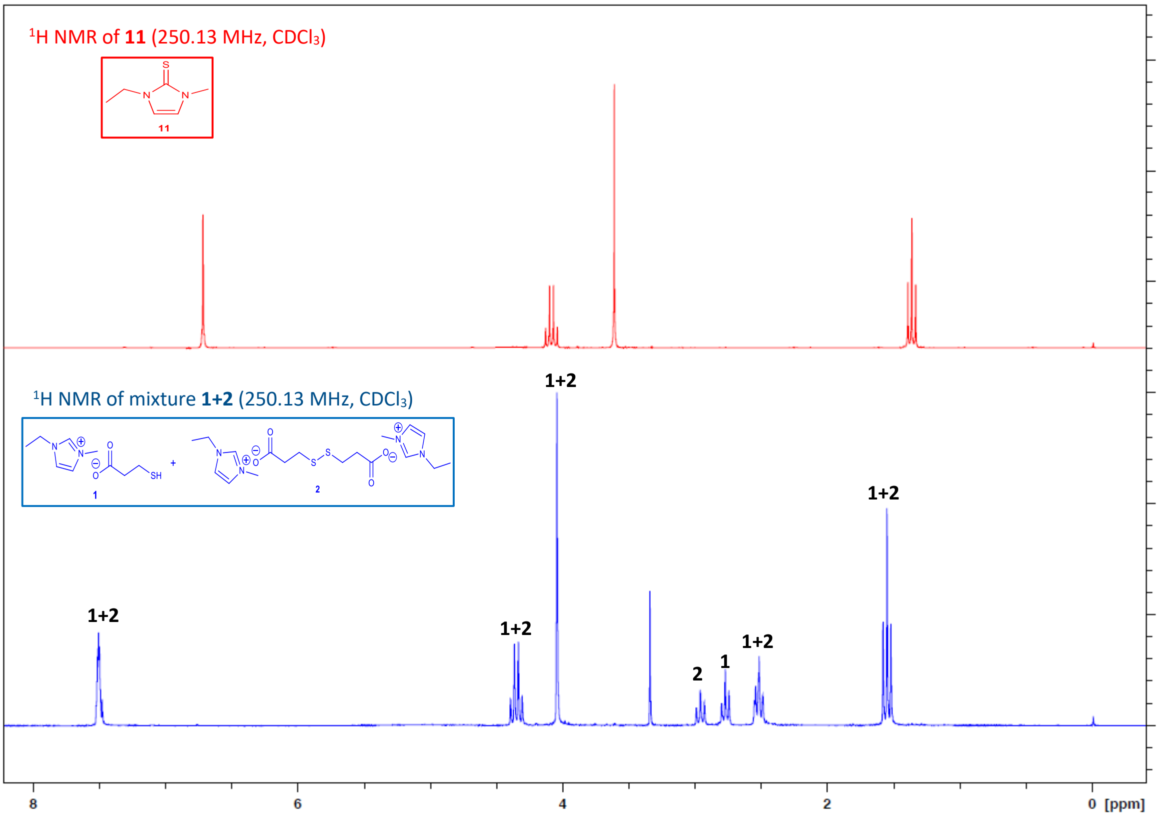

^{1}H NMR of 11 (250.13 MHz, CDCl$_3$)
11
^{1}H NMR of mixture 1+2 (250.13 MHz, CDCl$_3$)
1
2
1+2
1+2
1+2
1+2
1+2
2
1
[rel]
[ppm]

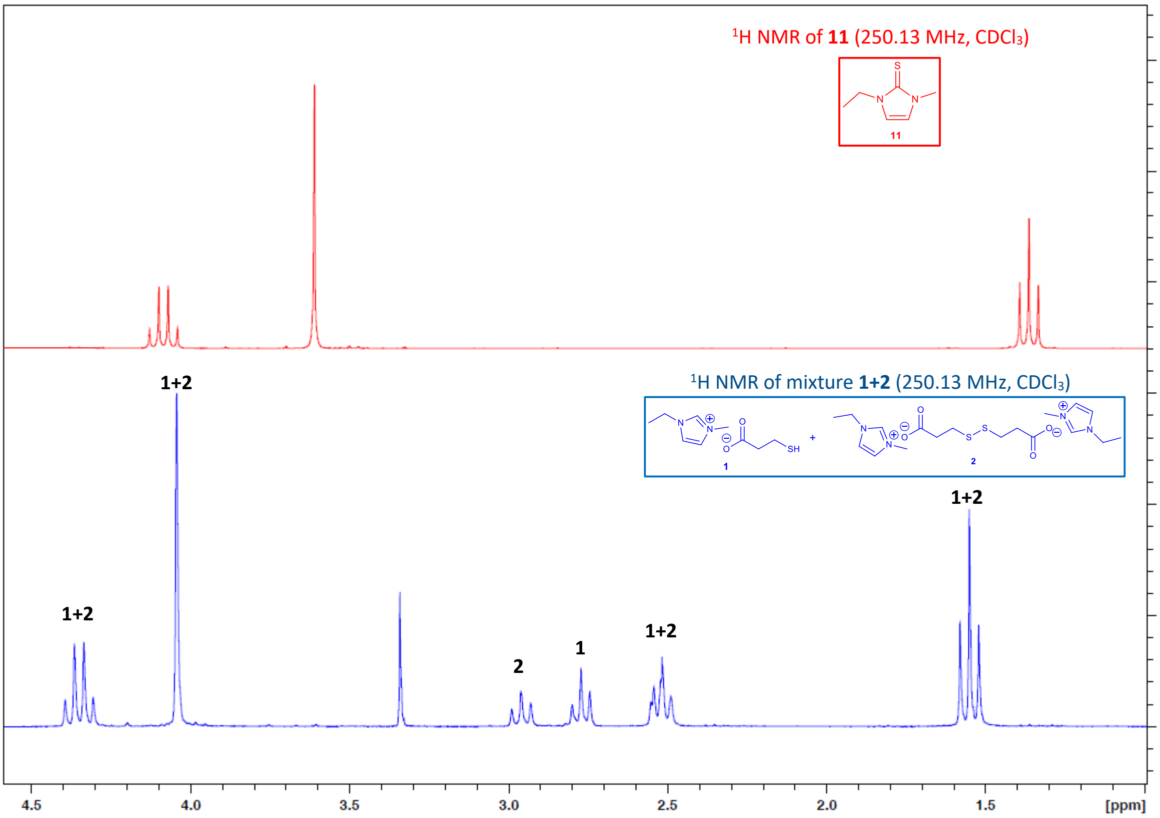

^{1}H NMR of 11 (250.13 MHz, CDCl$_3$)
11
^{1}H NMR of mixture 1+2 (250.13 MHz, CDCl$_3$)
1
2
1+2
1+2
1+2
1+2
2
1
[rel]
[ppm]

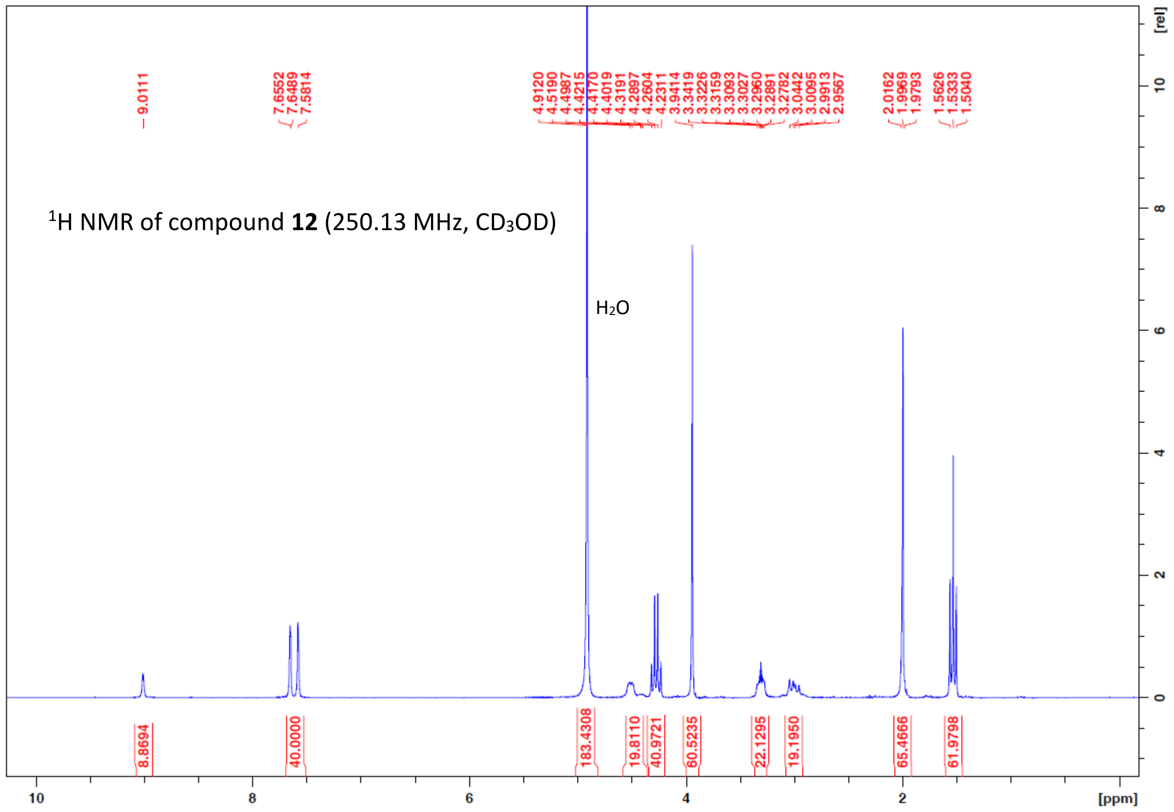
^{1}H NMR of compound 12 (250.13 MHz, CD3OD)
H2O
9.0111
7.6552
7.6489
7.5814
4.9120
4.5190
4.4987
4.4215
4.4170
4.4019
4.3191
4.2897
4.2604
4.2311
3.9414
3.3419
3.3226
3.3159
3.3093
3.3027
3.2960
3.2891
3.2782
3.0442
3.0095
2.9913
2.9567
2.0162
1.9969
1.9793
1.5626
1.5333
1.5040
8.8694
40.0000
183.4308
19.8110
40.9721
60.5235
22.1295
19.1950
65.4666
61.9798
[rel]
[ppm]

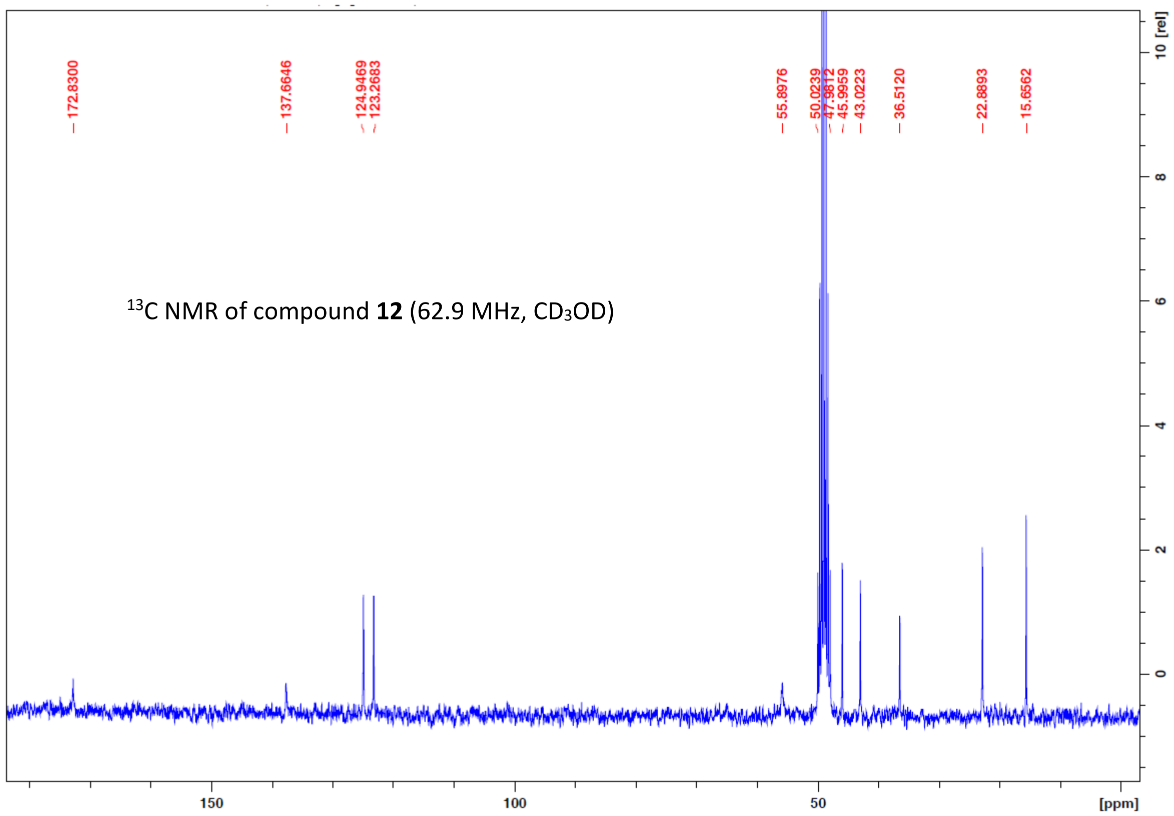
^{13}C NMR of compound 12 (62.9 MHz, CD3OD)
172.8300
137.6646
124.9469
123.2683
55.8976
50.0239
47.9812
45.9959
43.0223
36.5120
22.8893
15.6562
[rel]
[ppm]

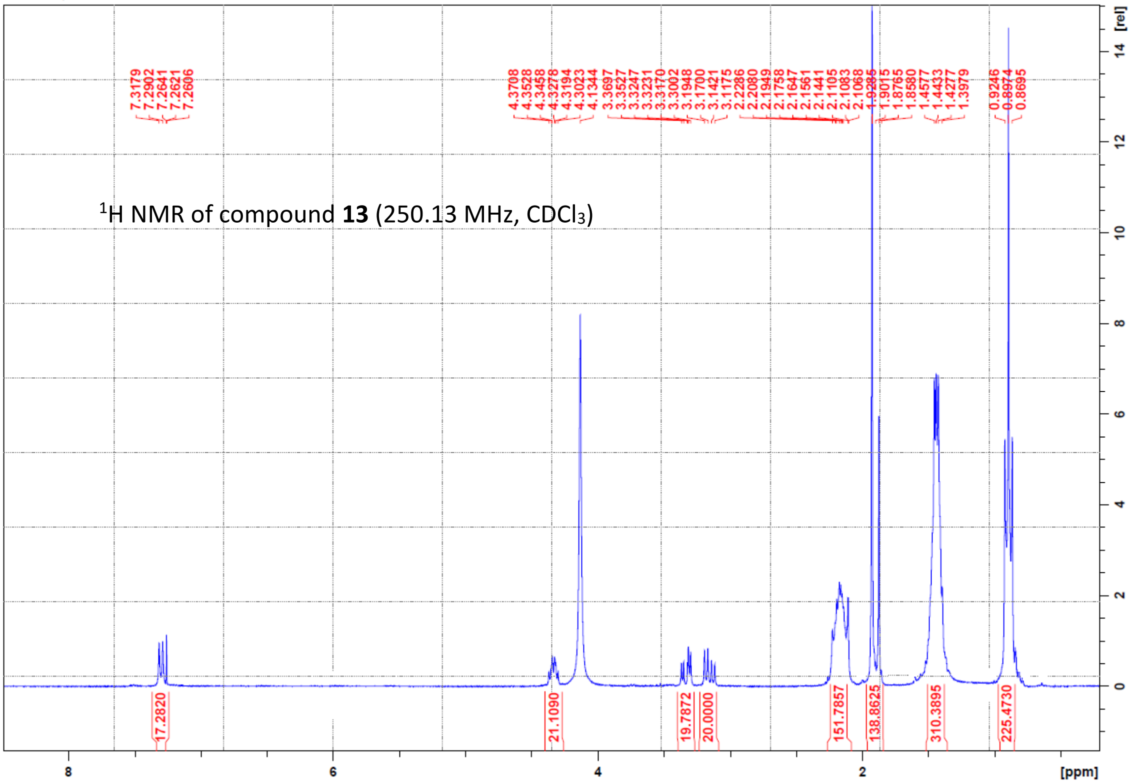
7.3179
7.2902
7.2641
7.2621
7.2606
4.3708
4.3528
4.3458
4.3278
4.3194
4.3023
4.1344
3.3697
3.3527
3.3247
3.3231
3.3170
3.3002
3.1948
3.1700
3.1421
3.1175
2.2286
2.2080
2.1949
2.1758
2.1647
2.1561
2.1441
2.1105
2.1083
2.1068
1.9285
1.9015
1.8765
1.8580
1.4577
1.4433
1.4277
1.3979
0.9246
0.8974
0.8695
^{1}H NMR of compound **13** (250.13 MHz, $CDCl_3$)
17.2820
21.1090
19.7872
20.0000
151.7857
138.8625
310.3895
225.4730
[rel]
[ppm]

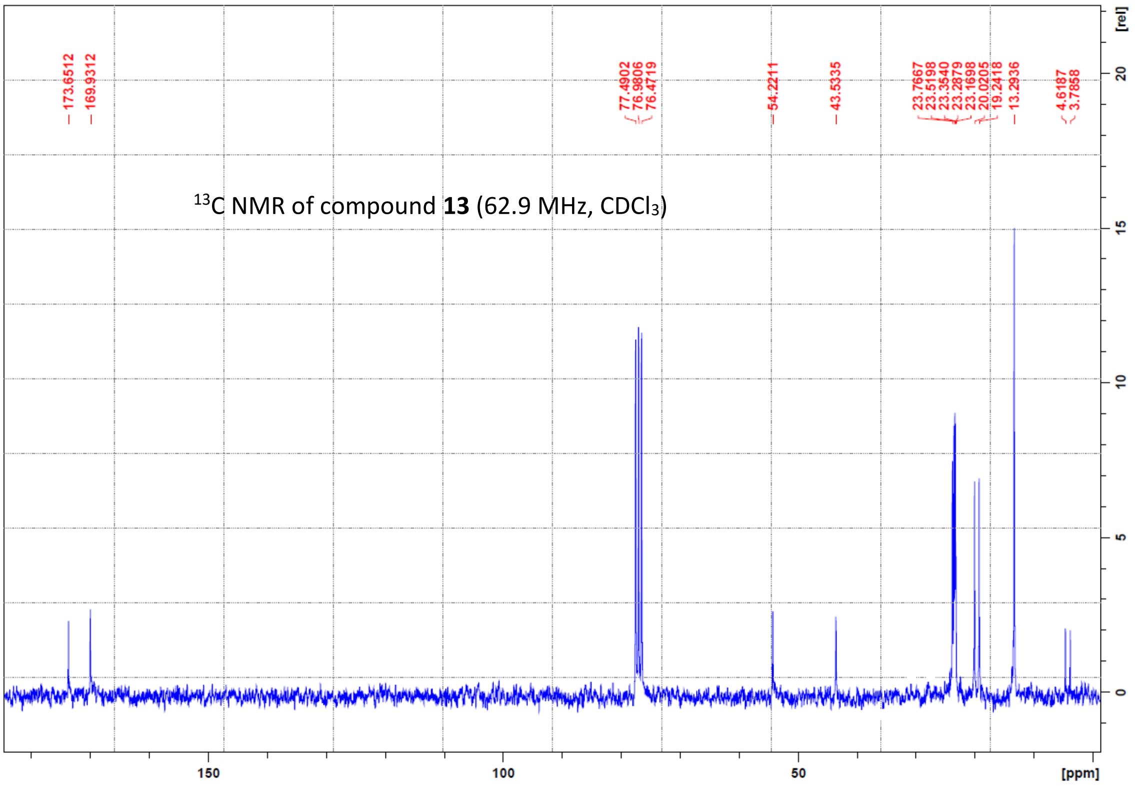
173.6512
169.9312
77.4902
76.9806
76.4719
54.2211
43.5335
23.7667
23.5198
23.3540
23.2879
23.1698
20.0205
19.2418
13.2936
4.6187
3.7858
^{13}C NMR of compound **13** (62.9 MHz, $CDCl_3$)
[rel]
[ppm]

COSY spectra of compound **13** (250.13 MHz, $CDCl_3$)

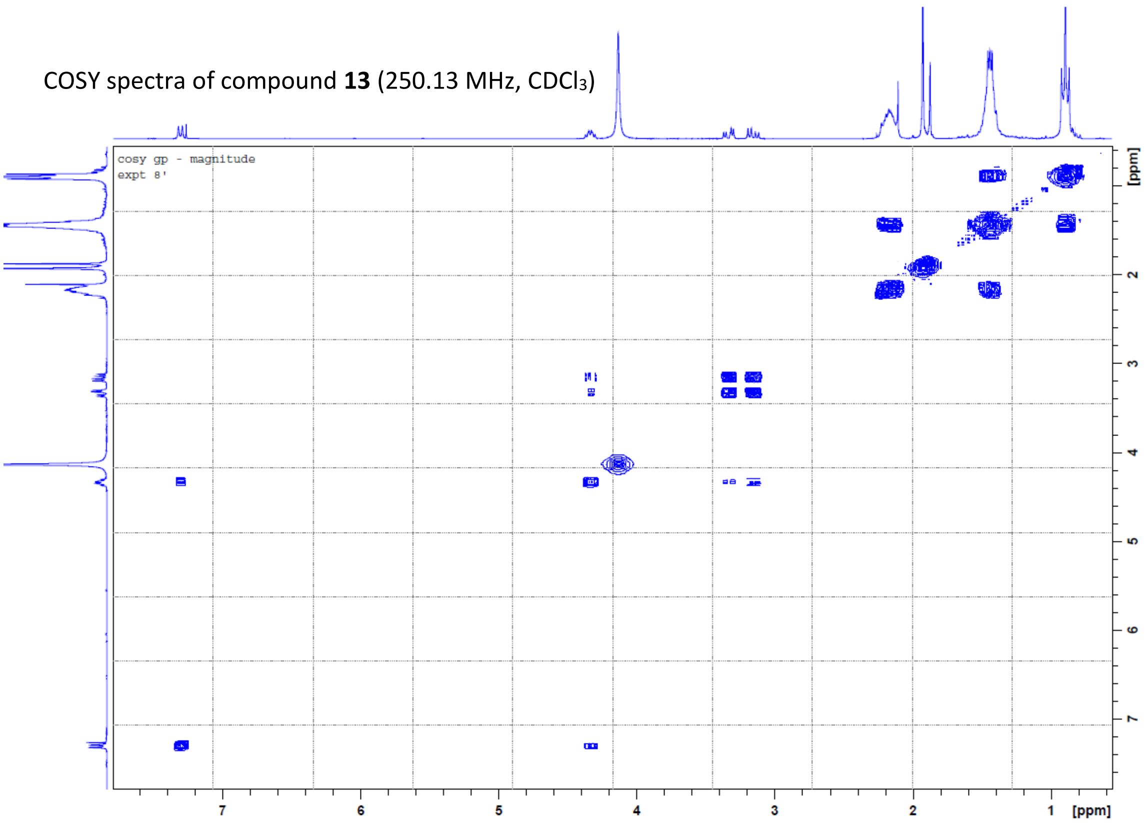

HSQC spectra of compound **13** (250.13 MHz, $CDCl_3$)

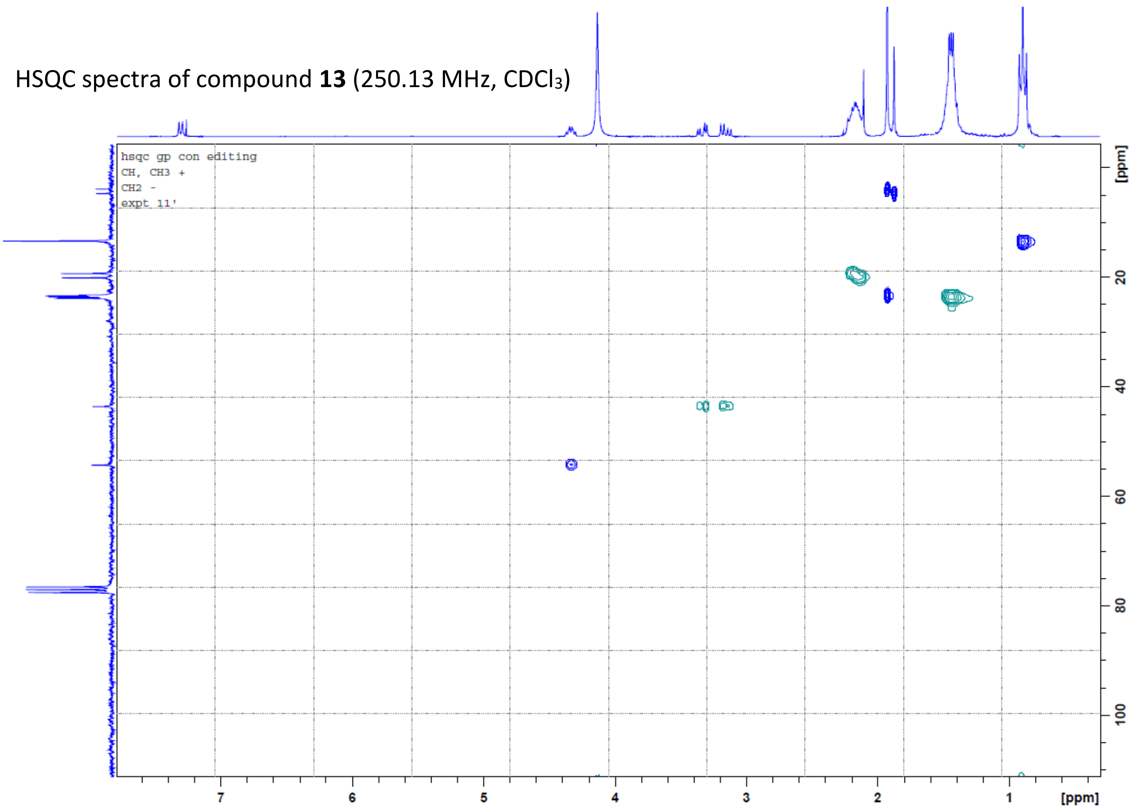

TGA profiles of ILs 2-3, 5-6 and 8-9

Figure S1. Thermal gravimetric analysis and derivative of IL **2**.

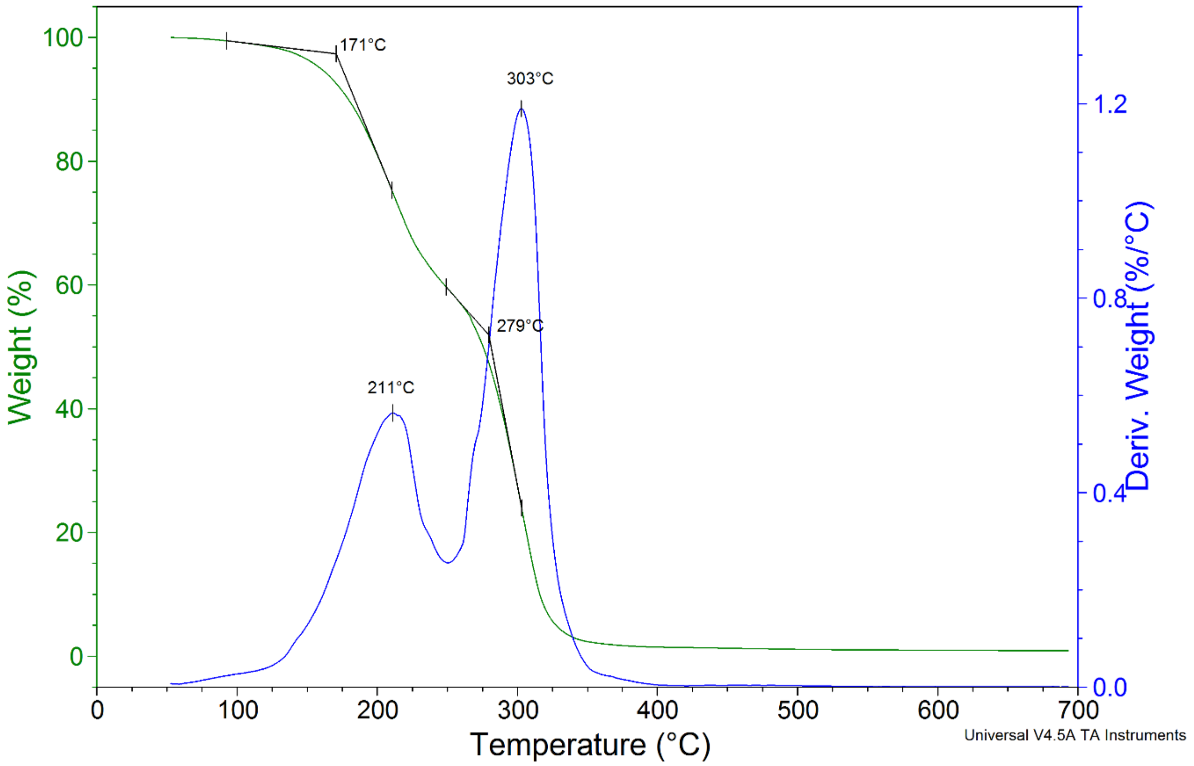

Figure S2. Thermal gravimetric analysis and derivative of IL **3**.

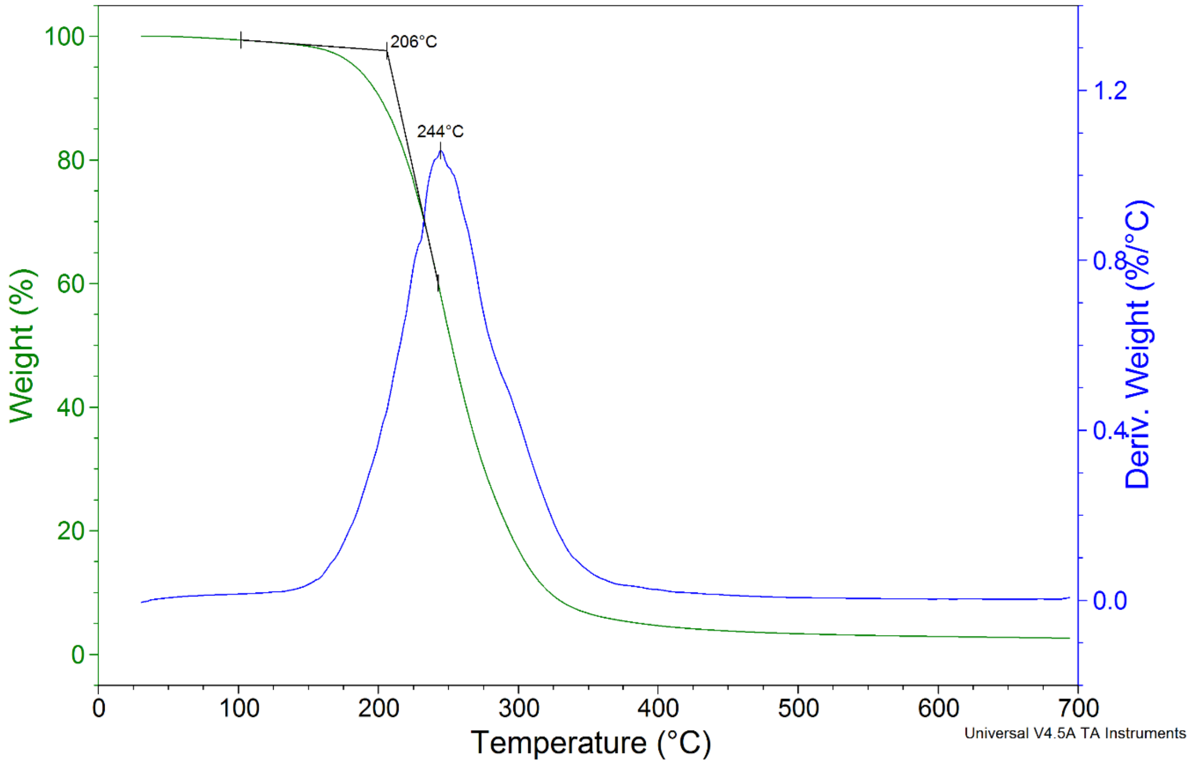

Figure S3. Thermal gravimetric analysis and derivative of IL **5**.

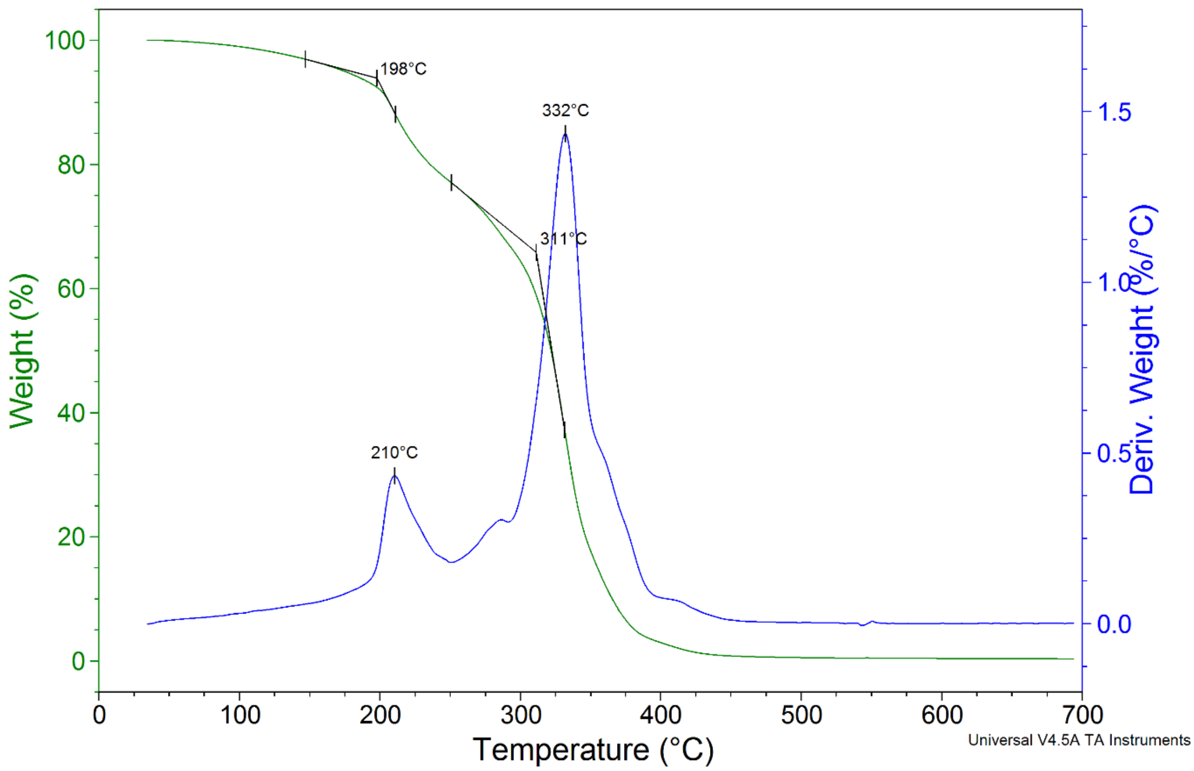

Figure S4. Thermal gravimetric analysis and derivative of IL **6**.

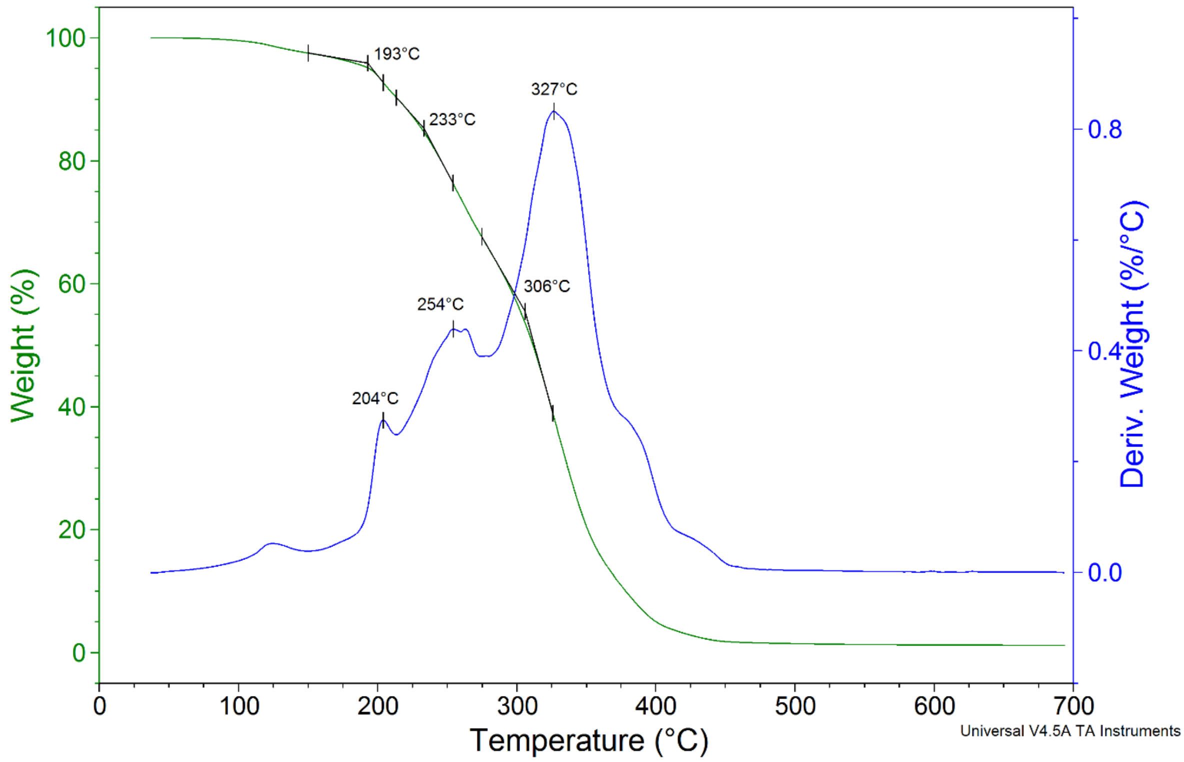

Figure S5. Thermal gravimetric analysis and derivative of IL **8**.

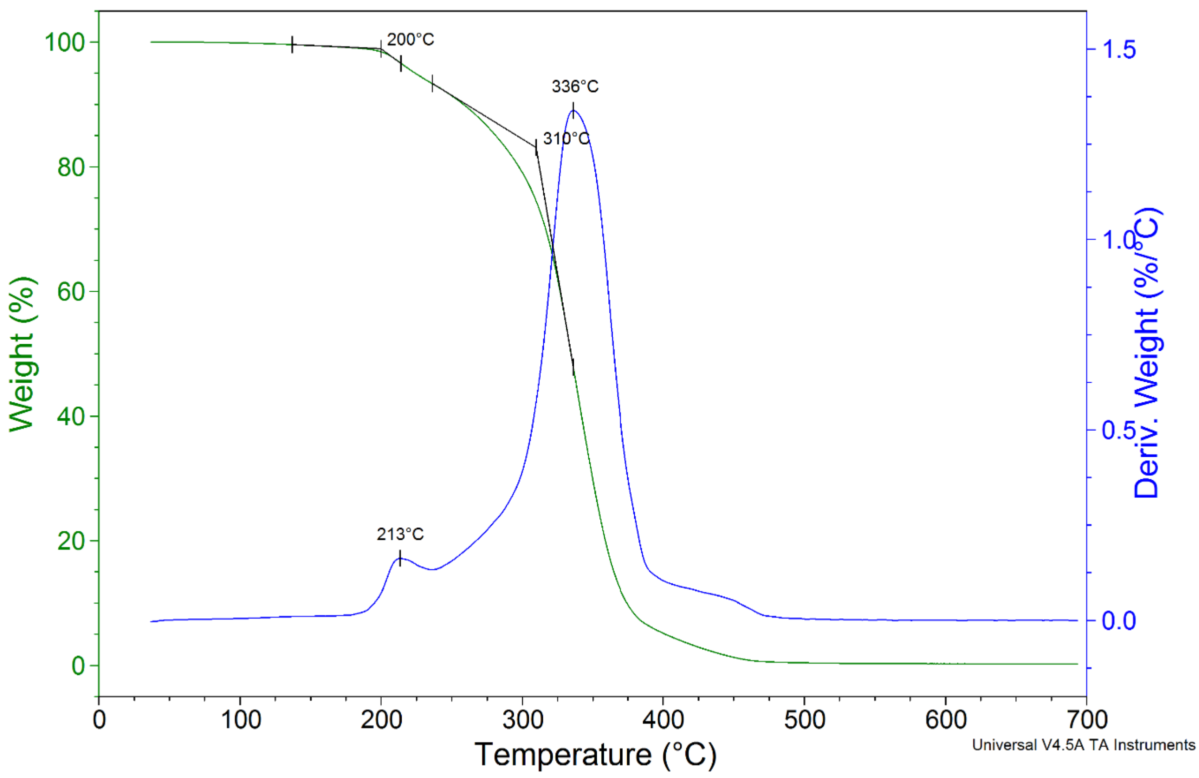

Figure S6. Thermal gravimetric analysis and derivative of IL **9**.

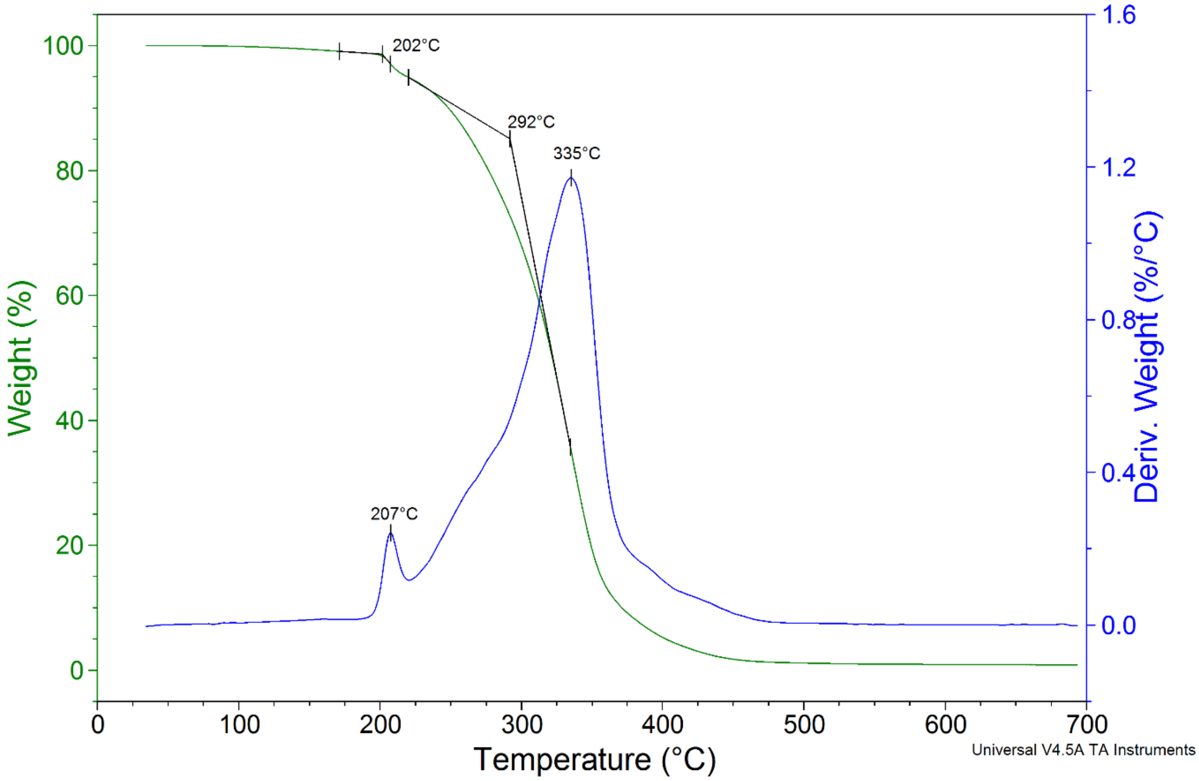

DFT optimized geometries

```
58
 Minimal cluster
 C 0.3978205972 -2.2335886883 0.1878869286
 H 0.9016566667 -1.3361358152 -0.1812534335
 H -0.3534245964 -1.8859663347 0.9099826437
 C 1.3781269957 -3.1348115751 0.9209571311
 H 1.6858665944 -2.6915688321 1.8699481193
 H 0.9213902167 -4.1080525801 1.1177689917
 C 3.53292519 -1.7686434567 -2.3547703956
 H 2.4595915872 -1.8326045874 -2.5286969909
 H 4.0254493193 -2.6499162406 -2.7687436415
 C -0.3701118655 -2.8837586052 -0.9800096428
 O -0.3728229764 -4.1271687824 -1.0803759692
 O -0.9468725651 -2.0731183771 -1.7748896754
 C 4.0943237067 -0.4813495494 -2.9655885697
 H 4.1582834248 -0.6307220868 -4.0502110477
 H 5.1041547592 -0.2766917995 -2.6031055763
 S 2.9227267022 -3.616640924 0.0201097036
 S 3.8336665398 -1.8037527206 -0.5233216231
 C 3.1937036297 0.7457803411 -2.7167801733
 O 1.9853168043 0.6335318194 -3.1032734568
 O 3.6894331625 1.7396755827 -2.1484126057
 C 0.0135214002 -3.2231457892 -4.2312080014
 H -0.5451125329 -2.8994473994 -3.3361491547
 C 1.165404031 -3.1374434633 -6.1198965524
 H 1.617832186 -2.6844688847 -6.9848467295
 C 1.1463254534 -4.4318078199 -5.6995439324
 H 1.5773812792 -5.3158532883 -6.1351690262
 N 0.4239910293 -4.4642080508 -4.5213998342
 N 0.4521890182 -2.4016757628 -5.193983344
 C 0.1540461832 -5.6604363404 -3.6895775359
 H -0.4216624204 -6.3574574218 -4.3041750207
 H -0.4497802197 -5.3101282569 -2.849892024
 C 1.4381584238 -6.2934420476 -3.1659830452
 H 1.1772932777 -7.1665220068 -2.563628645
 H 2.1009709704 -6.6261489752 -3.9700742922
 H 1.9721007874 -5.5924355814 -2.5221287836
 C 0.2632102821 -0.9415370186 -5.2019858554
 H -0.7587767206 -0.7243995756 -4.8950215011
 H 0.9671186189 -0.457344654 -4.5136949071
 H 0.4157179567 -0.5863306961 -6.2204912935
 C 0.5274698832 1.9109873616 -0.9471601325
 H 1.116824453 1.5689801754 -1.8178565724
 C -0.0662739106 2.7726216473 1.004971509
 H 0.0610175313 3.2843617413 1.9428016898
 C -1.1751317136 2.2282857766 0.4342024301
 H -2.1922035765 2.1740728052 0.7813467478
```

N -0.785129104 1.7001139383 -0.7805129587
N 0.9824086088 2.5676286839 0.1285601376
C -1.6431288972 0.9653343548 -1.7238671239
H -2.6809442935 1.1529774578 -1.4513912647
H -1.4613484751 1.3460850843 -2.7284055807
H -1.4195417434 -0.1082943975 -1.6783640067
C 2.3899717651 2.9736510548 0.3478406113
H 2.9019801549 2.8293009273 -0.607372979
H 2.3772367913 4.0365427373 0.6016150177
C 3.058831417 2.1429037663 1.4381325803
H 3.0886827741 1.0889752471 1.1546763476
H 4.089425084 2.4842593693 1.5581992656
H 2.5547262541 2.2388326833 2.4043078038

18
EMIM derived carbene
C -4.1687611668 3.634425134 0.2478490133
C -2.6585848032 1.9216326602 0.016146751
C -4.6382116008 2.5875836444 0.9687774881
H -5.5273539403 2.4936903883 1.570392165
H -4.5767030496 4.6210739816 0.1012889424
N -2.9704354544 3.2101105602 -0.3162244677
N -3.7097065622 1.5640417157 0.8125082797
C -2.1469387031 4.0202795484 -1.2116308809
H -2.0579224545 5.0246254511 -0.7858052035
H -1.1568157892 3.5649127202 -1.2053036769
C -2.700271204 4.0846403399 -2.6351208179
H -3.6978198978 4.5317009631 -2.6578284563
H -2.0442930311 4.6900360688 -3.2669530781
C -3.825592705 0.2540380633 1.4341267918
H -4.7389257871 -0.2504419719 1.1075231113
H -2.9616661184 -0.332318035 1.1286535809
H -3.8358571285 0.3408301017 2.5240115952
H -2.7641787542 3.0821222061 -3.0639077375

76
Minimal cluster + carbene
C 0.2620639053 1.3550208082 -1.3025669748
H 0.521422985 0.8704640217 -0.35766486
H 1.1509028583 1.2767309557 -1.9442777052
C -0.0483660897 2.8303887838 -1.0898739784
H 0.8205156405 3.3400352483 -0.6775967192
H -0.318936447 3.3047434705 -2.0339489467
C -0.1141502005 1.2723746424 2.8855183146
H -0.6708415167 0.803691235 2.0720162289
H -0.8094225346 1.6452886692 3.640270159
C -0.8564075772 0.5348183194 -1.9651657386
O -1.9009051816 1.1147419651 -2.3263488487
O -0.6439636513 -0.7204467499 -2.0481735349
C 0.8348136455 0.2477083651 3.5116096723

H 0.2257124278 -0.462936388 4.0873572139
H 1.5218578685 0.7214100352 4.2150486371
S -1.4841496886 3.1796021052 0.0189905412
S 0.7922006032 2.7282367631 2.2233117154
C 1.6467156707 -0.5862221337 2.5014361628
O 1.0159575739 -1.031064535 1.4879491026
O 2.8552371282 -0.7863267421 2.7484155688
C -2.9190451142 -1.430260884 -0.4572527663
H -2.0672849647 -1.2634091177 -1.1422015929
C -4.1275357329 -2.0637188794 1.2860935778
H -4.3275477691 -2.5216691368 2.2391546231
C -4.9477280183 -1.3968532116 0.4291234021
H -5.9953138013 -1.1636443047 0.5013825296
N -4.1753403619 -1.0109601133 -0.6504986767
N -2.8673748312 -2.0708930455 0.7183667079
C -4.6267704765 -0.22079375 -1.8200114537
H -5.3825524693 -0.8147830102 -2.3405952274
H -3.7455763697 -0.0883250228 -2.4506033992
C -5.1576436632 1.1485508255 -1.4128950337
H -5.4698314396 1.6839321301 -2.3122715256
H -6.0203575138 1.0835210717 -0.7434097366
H -4.3663196938 1.7306349465 -0.9382766773
C -1.6564823188 -2.6777909399 1.2963133876
H -1.4310788364 -3.609089554 0.7747624585
H -0.807285343 -1.9881670587 1.2319733074
H -1.8575462722 -2.8946175314 2.3444283195
C 3.0774868077 -1.334385107 -0.5548017286
H 2.3078957952 -1.2683420586 0.2401056734
C 4.9613006592 -1.1017643759 -1.6997387464
H 5.9666699708 -0.7721635354 -1.8960596331
C 4.1034680814 -1.8395940258 -2.4548856523
H 4.2191903708 -2.2750393517 -3.4319888085
N 2.9378368627 -1.9718968859 -1.7259692877
N 4.3075625148 -0.8024636849 -0.5199151971
C 1.7268635546 -2.6906071822 -2.1505465176
H 1.8611619569 -2.9894267382 -3.1891986021
H 1.5999813485 -3.5804769459 -1.5323829733
H 0.8618455374 -2.022255021 -2.0669241313
C 4.8579068274 0.0097948061 0.588950921
H 4.3478934236 -0.3236844421 1.4975060879
H 5.9188941693 -0.2411407212 0.6554542362
C 4.648763083 1.5046258557 0.3655172537
H 3.5846771284 1.7501316542 0.3591484623
H 5.1071686123 2.0527141539 1.1919809186
H 5.1024758467 1.8499621732 -0.5682983039
C -0.9039599482 5.9340458235 2.6527650852
C -0.7747425888 3.8658390415 1.6243922102
C -1.8207148718 5.1569033256 3.2443196415
H -2.4704662094 5.3582180669 4.0789315541
H -0.5915991248 6.937794767 2.8883329975
N -0.318433132 5.2221133629 1.6043254173

N -1.81520034 3.9008608661 2.6125883948
C 0.8168569326 5.715909435 0.8448147107
H 1.4156496925 6.3519260248 1.5059336492
H 1.4547082626 4.8675859625 0.5871315266
C 0.4249752627 6.4969440473 -0.4141099085
H -0.1743174877 7.3737213979 -0.1543118232
H 1.320761886 6.8408300853 -0.941268098
H -0.1643242934 5.8774624285 -1.0922539581
C -2.9984119158 3.0586856697 2.6345151079
H -3.8097156533 3.4632482802 2.0139582608
H -2.7599043666 2.0593881276 2.2806108771
H -3.3538728855 2.9851250975 3.6663629294

76

Minimal cluster + carbene (encounter complex)

C -2.3167477119 0.810606009 -2.2954939511
H -2.6417773507 0.3011805416 -1.3838967319
H -3.2195585169 1.2633986809 -2.7272792542
C -1.7616903383 -0.1877953794 -3.2987189838
H -2.5562870141 -0.8024971231 -3.7261359278
H -1.2481196566 0.3381130829 -4.1076265377
C -0.4207750911 -1.6129165859 0.3579075577
H -0.5844807807 -0.5365141798 0.335172032
H 0.6413080739 -1.8230427945 0.221913607
C -1.3762139919 1.9712772055 -1.9051108575
O -0.3467510405 2.162254453 -2.5852583751
O -1.7336086087 2.6465319219 -0.8891518113
C -0.9418184944 -2.2013742514 1.6726176432
H -0.2117377034 -1.9622438807 2.4550951461
H -1.021300008 -3.2895633176 1.6178864989
S -0.4442676949 -1.3450202307 -2.704607249
S -1.3027966952 -2.3740506136 -1.0874273754
C -2.2969509291 -1.6005527209 2.1013709353
O -2.3251803173 -0.3335291456 2.2183873966
O -3.2588996794 -2.3752383674 2.280704596
C 0.9078253261 2.4240408214 0.3392972214
H 0.0636895684 2.8225043789 -0.2302435607
C 2.0587989575 1.5085487365 1.990065496
H 2.2397976353 1.1371505534 2.983825957
C 2.8676510484 1.5446233086 0.8945182716
H 3.8844245188 1.1898973833 0.7598502424
N 2.1261024452 2.1197693231 -0.1241374757
N 0.8454717216 2.0637530061 1.6256332821
C 2.5746309376 2.3693308297 -1.5126202282
H 3.3340717437 3.1551054372 -1.474351241
H 1.6928852956 2.7272262937 -2.0467833837
C 3.1127369859 1.1069703945 -2.1750906581
H 3.4155531969 1.3503868828 -3.1965108819
H 3.9825542058 0.70607834 -1.6483459629
H 2.3315440198 0.3462803239 -2.2295422965

C -0.3578581124 2.1586301916 2.4640265815
H -0.9995416334 2.9294388492 2.0419720706
H -0.8994675942 1.2059529128 2.4681969827
H -0.057369548 2.4432224041 3.4728299437
C -4.8886766581 0.3489184888 1.0718117164
H -3.9956355553 0.0468288758 1.6503006496
C -6.7692931514 0.3119310733 -0.0973963401
H -7.644703091 -0.1153552892 -0.5542085891
C -6.3172770086 1.5952674579 -0.0736995034
H -6.7208524075 2.4930940369 -0.5083793206
N -5.1487219375 1.5972244061 0.6618694706
N -5.8676514381 -0.4484516142 0.6231001139
C -4.2760931402 2.7577089683 0.9018072522
H -4.8720731898 3.6611152733 0.7765183884
H -3.9113649332 2.7066922781 1.9268507111
H -3.4314176332 2.744841675 0.2001117205
C -5.9437668955 -1.910707491 0.8451726
H -5.1764764018 -2.1473693311 1.5875384153
H -6.933424604 -2.1180988075 1.2599518005
C -5.6966665597 -2.695615975 -0.439043488
H -4.6935346132 -2.4991130086 -0.8225481967
H -5.7650658317 -3.7630563467 -0.2175933265
H -6.4288232375 -2.4631232391 -1.2179951667
C 7.3811351869 -1.8647441036 0.6633805169
C 5.9905459843 -0.0459206438 0.6141332954
C 8.1597092773 -0.763209655 0.7892446288
H 9.2268766862 -0.6676984503 0.9029848806
H 7.6454775327 -2.9089868474 0.6440592869
N 6.0729418024 -1.4080184101 0.5607624052
N 7.2970864367 0.325797076 0.7557413458
C 4.9117843997 -2.2822639802 0.3770106811
H 4.9546416531 -3.07398886 1.1310871487
H 4.03445062 -1.6718311991 0.5882764275
C 4.8280966609 -2.8794537604 -1.026847356
H 5.7001379611 -3.4989916538 -1.2521465001
H 3.9373966976 -3.5067919205 -1.1130477367
H 4.764454811 -2.0905495092 -1.779256608
C 7.7317295416 1.7104651303 0.8646330508
H 8.4069534479 1.9691277369 0.0450595543
H 6.8485591243 2.3434883688 0.8136728306
H 8.2422822945 1.8819205751 1.8157255227

76
Minimal cluster + carbene (transition state)
C,0,1.3162626833,0.0934073398,-3.1846134112
H,0,0.5044339892,-0.5872197783,-2.924238324
H,0,1.4501158461,0.0435557626,-4.2737489589
C,0,2.6223596093,-0.333193788,-2.5182853145
H,0,2.8688745136,-1.3761346877,-2.7249095824
H,0,3.44105689,0.2985694186,-2.8618505309
C,0,1.1840900412,-0.2304337411,2.3441047655

H,0,0.8969557585,0.2482409098,1.4086461594
H,0,1.8150916256,0.470138175,2.8950114782
C,0,0.8989383532,1.5361557182,-2.8285655484
O,0,1.832742916,2.3863705506,-2.7479202535
O,0,-0.3196336401,1.7580493847,-2.6197014252
C,0,-0.0781712378,-0.5834166013,3.1495532687
H,0,-0.1995550388,0.1466721363,3.961050974
H,0,0.009923501,-1.5569918967,3.633692765
S,0,2.6015802665,-0.1369219769,-0.6769715639
S,0,2.2722395716,-1.6810780455,1.961179894
C,0,-1.4108969884,-0.5475227595,2.3735757326
O,0,-1.4898770932,0.2381762772,1.3732690869
O,0,-2.3430385061,-1.2634180395,2.8021298605
C,0,1.3460049978,3.6212135392,-0.2574502111
H,0,1.3614614999,3.2989350111,-1.3158005462
C,0,0.7355072208,4.0193469783,1.830647544
H,0,0.0674054552,4.0760403499,2.6723343556
C,0,2.0679850051,4.2765440335,1.733493398
H,0,2.7737575198,4.6070861842,2.475106435
N,0,2.429465993,4.0202357993,0.4222701763
N,0,0.3065094865,3.6158225259,0.5800406299
C,0,3.7808589204,4.104612782,-0.1638296407
H,0,4.1182333814,5.1407097408,-0.076365384
H,0,3.6603556167,3.8704563945,-1.2220023277
C,0,4.7617514922,3.1402294875,0.4962173407
H,0,5.7341799907,3.229241495,0.0059377478
H,0,4.901762623,3.3632596312,1.5570530123
H,0,4.4174999633,2.109248241,0.395451124
C,0,-1.0516439724,3.177174136,0.2232773516
H,0,-1.0439346599,2.9345732179,-0.8410049622
H,0,-1.2939972665,2.2774491889,0.795165555
H,0,-1.7514451196,3.9876078824,0.435544408
C,0,-3.8713113924,-0.3893460645,-0.1289751283
H,0,-2.9594514185,-0.1673188298,0.4722527998
C,0,-5.9086005848,-1.0466627775,-0.7075258156
H,0,-6.8875393019,-1.4735282662,-0.5769448055
C,0,-5.3139927412,-0.5151229618,-1.8088930977
H,0,-5.6758591706,-0.3912217212,-2.8150011047
N,0,-4.0468194891,-0.1129273618,-1.4291799061
N,0,-4.9957133442,-0.9595661191,0.3274757334
C,0,-3.0515238721,0.517385331,-2.3100103623
H,0,-2.8945715837,-0.1130867233,-3.1845272765
H,0,-3.4069363879,1.4978615813,-2.6267239265
H,0,-2.1009452526,0.6398694561,-1.7969644786
C,0,-5.2015224789,-1.4262658141,1.7184418022
H,0,-4.2870078532,-1.1670472233,2.2631384655
H,0,-6.0510319301,-0.8700671012,2.1231889802
C,0,-5.4337661661,-2.9315148469,1.7874368305
H,0,-4.5649937954,-3.4703717359,1.4042534383
H,0,-5.5666056364,-3.2184507769,2.8327584882
H,0,-6.3224349102,-3.2498982069,1.2345696185

```
C,0,1.8845289569,-3.9859870464,-0.7950434356
C,0,2.4637686011,-1.8794257351,-0.0896107738
C,0,3.2103085002,-3.9931203353,-0.5886077741
H,0,3.9231285095,-4.7952746316,-0.6814154231
H,0,1.2346152932,-4.7891902482,-1.093245975
N,0,1.4193951074,-2.6653701233,-0.6613632458
N,0,3.6186458534,-2.6832936284,-0.310990802
C,0,0.0211920436,-2.365028318,-0.3657220026
H,0,-0.2243366717,-2.714435654,0.64449984
H,0,-0.0842551192,-1.2798025373,-0.3588309131
C,0,-0.9361556634,-2.9747004226,-1.3870033347
H,0,-0.9060627051,-4.0674311527,-1.3834918422
H,0,-1.9598931642,-2.6815194783,-1.1395446635
H,0,-0.7110195848,-2.6305644676,-2.3997772938
C,0,4.932028084,-2.352710293,0.2050607774
H,0,5.1124350983,-1.2877417666,0.0586857124
H,0,5.0303932668,-2.5803275505,1.2717507105
H,0,5.6812336954,-2.9151224261,-0.3570008942
```
